# Synthesis of sequence-defined acrylate oligomers *via* photo-induced copper-mediated radical monomer insertions†
†Electronic supplementary information (ESI) available: Includes used materials, characterization techniques and experimental procedures, NMR and ESI-MS spectra for all synthesized compounds. See DOI: 10.1039/c5sc02035b


**DOI:** 10.1039/c5sc02035b

**Published:** 2015-07-03

**Authors:** Joke Vandenbergh, Gunter Reekmans, Peter Adriaensens, Thomas Junkers

**Affiliations:** a Polymer Reaction Design Group , Institute for Materials Research (IMO-IMOMEC) , Universiteit Hasselt , Martelarenlaan 42 , B-3500 Hasselt , Belgium . Email: thomas.junkers@uhasselt.be; b Nuclear Magnetic Resonance Spectroscopy Group , Institute for Materials Research (IMO-IMOMEC) , Universiteit Hasselt , Agoralaan Building D , B-3590 Diepenbeek , Belgium; c IMEC Associated Lab IMOMEC , Wetenschapspark 1 , B-3590 Diepenbeek , Belgium

## Abstract

Photo-induced copper-mediated radical polymerization is used to synthesize monodisperse sequence defined acrylate oligomers *via* consecutive single unit monomer insertion reactions and intermediate purification of the compounds by column chromatography or preparative recycling size exclusion chromatography. Monomer conversions are followed during reaction by means of infrared spectroscopy. When reaction conditions are chosen carefully and any residues from chlorinated solvents are avoided, 100% pure Br end capped sequence defined oligomers are obtained, demonstrating the convenience and power of photo-induced copper mediated radical insertion for establishing sequence control. Within this work, a library of sequence defined oligomers containing polar and apolar ester groups have been obtained, and for the first time, perfectly monodisperse acrylate pentamers became accessible from radical insertion reactions.

## Abstract 2

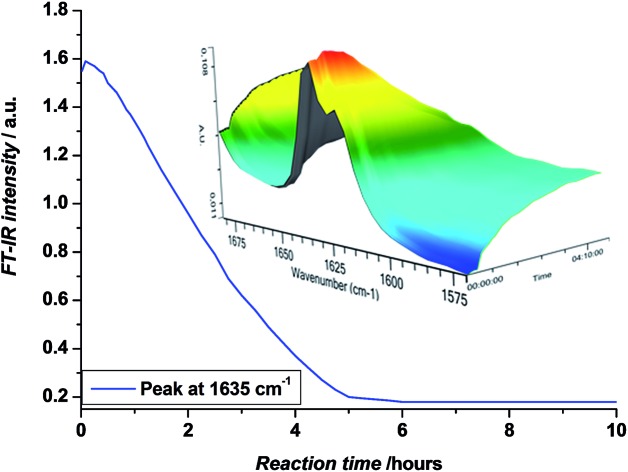
Photo-induced copper-mediated radical polymerization is used to synthesize monodisperse sequence defined acrylate oligomers *via* consecutive single unit monomer insertion reactions and intermediate purification of the compounds by column or preparative recycling size exclusion chromatography.

## Introduction

Ever since the development of controlled/living and earlier ionic polymerization techniques^1^,^2^ highly complex polymeric materials have become available. Furthermore, in combination with the implementation of a number of highly efficient orthogonal reactions (*click* chemistry),^3^ virtually any type of macromolecular architecture can be synthesized without many limitations in chain length, dispersity, topology or functionality.^4^,^5^ Question of accessibility of a material is not anymore if suitable synthetic handles are available, but only how much effort must be put into the synthesis routines. Yet, next to stereoregularity, one of the last major challenges in modern polymer chemistry is to reach a perfect ordering of monomers in a single chain, usually referred to as sequence-control.^6^–^9^ Polymeric materials in which the monomer sequence is controlled and defined, are the synthetic equivalent of natural peptide sequences, proteins or DNA. Sequence-controlled (SC) polymers may have various uses, but primarily allow for encoding information by the choice of the followed sequence. Like DNA, which uses a four-letter nucleotide code to store an enormous amount of information on its polymer chain, also synthetic polymers could be used for such purposes. This interesting application for sequence defined (SD) macromolecules has been presented in a recent paper of Colquhoun and Lutz.^10^ In principle, binary information can be stored on any co-polymer that uses 2 different monomers, represented as 0 and 1 bits.^11^ When considering such an oligomer with DP = 4, 16 (2^4^) different sequences can be prepared using a binary code. Off course, when for instance 4 different monomers are used to build up the tetramer, 256 (4^4^) different sequence possibilities exists. Using the family of acrylates – with in principle an unlimited number of different monomers – to build SD oligomeric structures hence opens vast possibilities to encode information in short sequences, in fact not unlike in peptide sequences. In addition, a number of techniques (*e.g.* NMR and mass spectrometry) have been described for reading such sequence-encoded information. In addition to molecular-level data storage applications, SC polymers, when being long enough, might fold into tertiary structures capable of performing a variety of advanced tasks such as selective transport, signal transduction or catalysis. Also, when attached to larger structures, SC oligomers can serve as molecular recognition elements or anchor points for chain folding.

Recently, a number of pathways towards controlling the monomer sequence in a polymer chain have been explored. Among them, some efficient synthetic procedures have been reported based on orthogonal reactions or step-growth polymerization techniques.^12^–^17^ By using these addition or condensation methods, dispersity of the final SC oligomer can be efficiently limited since only one monomer unit is added in each step. In this way, access to monodisperse materials is given, often by making use of solid support synthesis techniques. On the other hand, also chain growth polymerization mechanisms – thus methods which are inherently of statistical nature – have been investigated for the synthesis of SC polymers.^18^–^22^ Chain growth SC materials are generally more challenging in synthesis, yet feature the advantage that the –C–C– polymer backbone is much more stable compared to other products; also, by using vinyl addition reactions, functional groups in the SC material are very close to each other, which also distinguish them from many other materials, where significant spacers are built in the main chain with each monomer addition. A number of recent studies report on the synthesis of SC multiblock-copolymers, using reversible addition fragmentation chain transfer polymerization (RAFT),^23^–^25^ copper mediated radical polymerization (atom transfer radical polymerization, ATRP or single electron transfer living radical polymerization, SET-LRP),^26^,^27^ and most recently photo-induced copper mediated radical polymerization (photoCMP).^28^,^29^ With these techniques, mostly acrylate or acrylamide multiblock-copolymers can be synthesized with high efficiency and without the need for intermediate product purification (removal of monomer leftovers) in between the subsequent polymerization steps. Although an impressive number of blocks can be put in order this way, the resulting SC polymers are not monodisperse since the statistical nature of the applied radical chain growth technique cannot be avoided.

Lately, controlled radical polymerization techniques have also been applied for the synthesis of true monodisperse sequence defined (SD) oligomers by circumventing the statistical nature of the process. In a first study by Tsanakstidis and coworkers,^30^ the different reactivity of styrene and *N*-isopropyl acrylamide (NIPAM) was used to create single unit monomer insertion (SUMI) products by using the RAFT technique. This way, a monodisperse styrene–NIPAM dimer was successfully obtained. However, the concept was difficult to use for creation of larger oligomers due to the successively more similar radical reactivity of growing macro-RAFT oligomer chains.^31^ Using a similar approach, our group demonstrated the synthesis of SUMI oligomers in which up to 4 different acrylate monomers were built in a trithiocarbonate RAFT agent.^32^ The statistical nature of the radical insertions was thereby accepted, but after each insertion reaction the resulting product mixture was purified to isolate the desired SUMI product from its by-products (species in which either no monomer or more than one monomer was built in). In this case, the single unit concept has to be considered as inserting a single block with length unity and additionally a single unit monomer selection process is required to obtain the mono-disperse SUMI products. Of course, final reaction yields are limited due to reaction statistics and due to increasing difficulty of product isolation upon higher number of insertions.^33^ On the other hand, by using acrylate esters a broad range of functionalities can be introduced into a sequence, either by employing commercially available acrylates, or by using custom-designed acrylates obtained by simple esterification. Furthermore, in another recent study it was demonstrated that these SD oligomers can also be prepared using scalable continuous (micro)flow reactors and that their formation can be followed and optimized by *in situ* soft-ionization mass spectrometry (ESI-MS) analysis.^34^

**Scheme 1:**
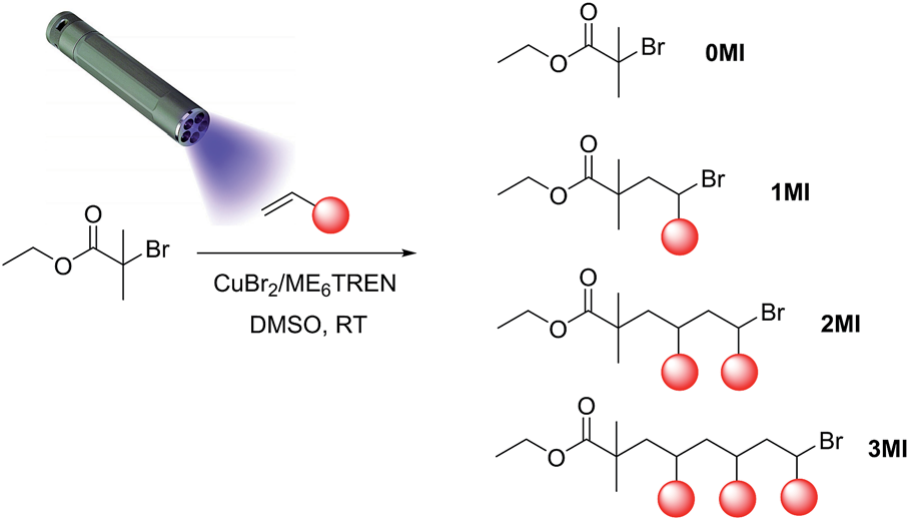
General reaction scheme for a photoCMP SUMI reaction, displaying the statistical nature of the radical insertion process.

While the RAFT approach to synthesize SC oligomers has proven to be very successful, it still displays some hurdles. The RAFT control group (trithiocarbonate or dithioester) is of considerable size and is prone to side reactions, especially at the higher temperatures that had been employed. Recently, a novel photo-induced CRP method was introduced, that makes use of specific copper dibromide/ligand pairs that are able to be photo-reduced.^35^,^36^ In consequence, a control equilibrium is established in the reaction by the interplay of copper reduction and subsequent activation of halogen-terminated species. To avoid mechanistic discussions – it is not fully clear if the polymerizations follow the SET-LRP or the ATRP reaction pathway – we had termed the synthesis technique photoCMP. In this particular study, we demonstrate the use of photoCMP as an alternative radical chain growth technique for the synthesis of SD oligomers *via* SUMI reactions (Scheme 1). PhotoCMP is a technique which provides high polymerization rates and mild reaction conditions, allowing to work in homogenous solution or in confined spaces, *i.e.* on surfaces.^37^ Polymerization can be carried out at room temperature, limiting undesirable side reactions such as backbiting in the case of acrylate polymerizations.^38^ For this reason, low-temperature polymerization or monomer insertion conditions are very desirable. Even though not often discussed, backbiting and other transfer to polymer reactions can severely damage the chain sequence by transferring the active radical centre to a remote chain position, which of course needs to be avoided as much as possible. Furthermore, photoCMP can be carried out to full monomer conversion, without loss of the Br end group, allowing to perform multiple SUMI reactions after each other. PhotoCMP has already been shown to be very effective in the synthesis of disperse multiblock copolymers,^39^,^40^ and its application towards SUMI reactions is a logical choice. Even though only very recently developed, photoCMP has demonstrated very high synthetic potential and its application to the synthesis of SD oligomers will not only advance the field of SUMI reactions, but also the photopolymerization technique by itself.

**Scheme 2:**
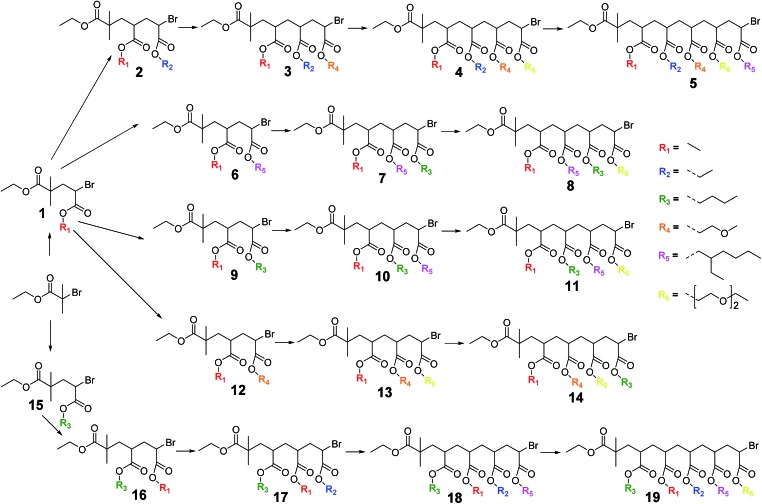
Library of sequence-controlled monodisperse oligomers prepared by photo-induced radical SUMI reactions.

PhotoCMP is indeed very capable in producing SUMI products of a variety of acrylate monomers and a library of SD oligomers was prepared (Scheme 2) consisting of specific sequences of 4 or 5 different acrylate units, marking the longest monodisperse SD oligomers made *via* CRP methods to date. Acrylates were deliberately chosen, since this type of monomers can be easily polymerized by means of photoCMP (unlike for instance styrenes, which cannot be controlled *via* photoCMP). Furthermore, acrylates with various ester side chains are easily available and exhibit very fast propagation rates, enabling fast reaction times and high conversion efficiencies. By limiting the monomer type to solely different acrylate units, reactivity differences between macro-initiators and monomers can be limited, and a higher number of insertions (up to pentamers) can easily be reached. This in contrast to the earlier SUMI study of Moad *et al.* where the use of a combination of styrene and NIPAM monomers (and hence employment of very different monomer reactivities), impeded the creation of larger oligomers than dimers.^30^ In this paper, we demonstrate the proof-of-concept of using photoCMP for the synthesis of SD acrylate oligomers. In principle, this approach can be extended to other monomers such as methacrylates and (meth)acrylamides as well, however this was not the intention of this concept study. We believe that when targeting information containing macromolecules, the type of applied monomer is not the most critical factor, but the ability to create a defined sequence *via* the ester side chains. The ability to establish sequence definition in the first place is crucial to allow molecular-level information encoding. Finally, optimizing the SUMI photoCMP for relatively simple (but yet representative) acrylates allows later to use virtually any functionality that is compatible with the chosen CRP method, such as *t*-butyl acrylate (which can be hydrolysed into pH-responsive acrylic acid) or pentafluorophenyl acrylate (which represents a “wild card” in a SUMI sequence due to the possibility of post-SUMI transesterification). In general, acrylates are among the most versatile monomer classes, since virtually any type of dedicated acrylate can be designed by transesterification of the ester bond. In the following, SUMI reactions *via* photoCMP are discussed in detail, with respect to time/yield correlation, purity of products and especially regarding purification of the desired products. Next to recycling SEC, we also demonstrate to which extend classical polar column chromatography can be used for purification, allowing for a significant acceleration of product isolation, while allowing concomitantly to increase yields compared to the previously described method.

## Results and discussion

In order to obtain the desired SD oligomers, a number of consecutive photo-induced SUMI reactions were carried out, and product mixtures were purified in between each subsequent insertion reaction. A number of different commercially available acrylate monomers was used for this purpose (Scheme 2). PhotoCMP is tolerant to a large number of functional groups and in principle, a vast variety of polar, apolar and ionic monomers could be employed. In here, we focused on a variety of polar and apolar acrylate esters, which are representative for the whole monomer family.

**Fig. 1:**
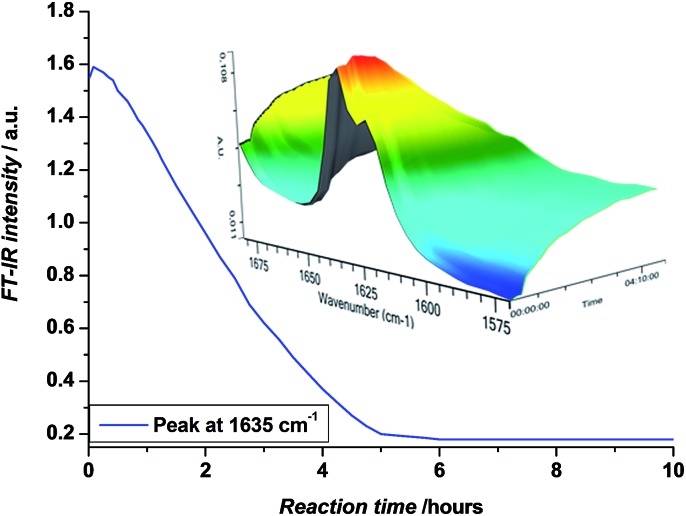
Decrease in absorbance at 1635 cm^–1^ and FT-IR waterfall profiles showing the disappearance of the 1640–1620 cm^–1^ vibrations, corresponding to the vinyl bond of the acrylate monomer, upon the photo-induced SUMI reaction towards **1**.

In a first step, methyl acrylate (MA) was inserted into a ethyl 2-bromoisobutyrate (EBiB) initiator. For this and all further SUMI reactions, the following ratios were applied: [M] : [I] : [CuBr_2_] : [ME6TREN] = 1 : 1 : 0.012 : 0.084. The monomer ratio was kept equal to the initiator ratio in order to preferentially built in only one single monomer unit (on average) during the radical insertion reaction. In a previous modelling study, we had shown that only little difference in the reaction yield must be expected when choosing equimolarity (and full conversion) over other monomer : initiator ratios.^33^ The copper/ligand ratio was before determined to be optimal for photoCMP processes.^35^ DMSO was used as a solvent. The mixture was purged with nitrogen in the dark to avoid any premature reaction and cooled in a water bath as soon as the UV light source was switched on to avoid heat-up of the reaction mixture by the reaction exotherm or by pure incidence of the UV-light. In this way, thermally induced side reactions (*e.g.* backbiting) are strictly limited. Furthermore, when photoCMP is applied, one needs to take care to stop the reaction before all monomer is depleted. When no monomer is available for reaction, bimolecular radical termination may eventually occur, leading to radical coupling and as a result loss of the bromine end group. Such scenario is best avoided to keep reaction yields as high as possible. Coupling products are – due to their quite different molar mass compared to the SUMI products – relatively easy to separate. Yet, only if maximum theoretical yields are achieved isolated yields can be optimized.^33^ Thus, the photo-induced SUMI reactions were *in situ* followed by FT-IR spectroscopy. The vinyl bond of the acrylate monomer has a vibration around 1640 cm^–1^, and the depletion of this signal during reaction can be followed in time (Fig. 1). In all cases, reactions were stopped (by switching off the light) when monomer conversions between 80–90% were obtained. Note that relative peak absorbance is directly proportional to monomer conversion.

Depending on the type of monomer and on the DMSO concentration, different reaction times were necessary to reach a high conversion (see Table 1). As can be seen from the table, insertion of the first monomer into the EBiB initiator was always slower compared to the subsequent SUMI reactions. This effect might be caused by several factors. Different reactivities are to be expected when comparing tertiary radicals generated from EBiB initiation in the first SUMI reaction with secondary radicals obtained from an acrylate macro-initiator in the following SUMI reactions. Furthermore, as described before, a chain length dependency of the monomer addition reaction might exist,^33^ giving rise to different reaction kinetics for first and subsequent SUMI reactions. Finally, the reaction solvent concentration may play a role. The first SUMI reactions (**1** and **15**) were carried out in 50 vol% of DMSO, while in the subsequent reactions higher dilutions (67–86 vol%) were used. At first glance, one would assume that higher dilutions lead to a decrease in reaction rate. However, in this case it is believed that this effect is overruled by the deeper UV light penetrating into the green coloured (Cu(ii)) reaction solutions. Mixtures with 50 vol% DMSO are rather opaque while diluted mixtures are more transparent, giving the incident light beam a better chance to also illuminate the center of the solution, which consequently speeds-up the reaction. Still, the reason that the first SUMI reactions are always significantly slower points to the relatively large difference in reactivity of the tertiary initiator radical compared to secondary acrylate-type radicals. In principle, SUMI reactions can be used to trace individual rate coefficients for each reaction step. The yield of reaction is – for the reasons stated above – not directly correlated to the specific monomer addition rate, but nevertheless can serve as a good indicator for the different reactivities. When taking chain-length dependencies into account, which predict faster insertions the shorter the chain, the overall reaction times that are required to reach full conversions can – at least in principle – be related to monomer reactivities. With increasing size of the ester side chain usually an increase in rate of polymerization is observed,^41^ which qualitatively holds true in the various SUMI reactions summarized in Table 1, even if it is sometimes counteracted by other influences, thus monomer concentration and SUMI product chain length.

**Table 1:** Reaction times, yields and end group quantification of SUMI products

Code	#SUMI	Reaction time, min	Isolated yield%	SUMI-Br%	SUMI-Cl%	SUMI-H%	Other SUMI%
1	SUMI-1	240	43	100	0	0	0
2	SUMI-2	60	47	91	9	0	0
3	SUMI-3	30	21	83	9	0	8
4	SUMI-4	80	18	93	7	0	0
5	SUMI-5	45	10	86	8	3	3
6	SUMI-2	155 + 210^*a*^	18	100	0	0	0
7	SUMI-3	45	27	69	31	0	0
8	SUMI-4	90	12	64	32	4	0
9	SUMI-2	60	43	100	0	0	0
10	SUMI-3	45	14	100	0	0	0
11	SUMI-4	50	8	94	6	0	0
12	SUMI-2	45	41	100	0	0	0
13	SUMI-3	45	22	100	0	0	0
14	SUMI-4	45	13	92	4	4	0
15	SUMI-1	225	40	100	0	0	0
16	SUMI-2	30	43	100	0	0	0
17	SUMI-3	65	24	58	37	0	5
18	SUMI-4	30	12	52	48	0	0
19	SUMI-5	45	2	58	42	0	0

^*a*^SUMI-2 has been obtained after 2 subsequent insertion reactions, without intermediate purification.

**Fig. 2:**
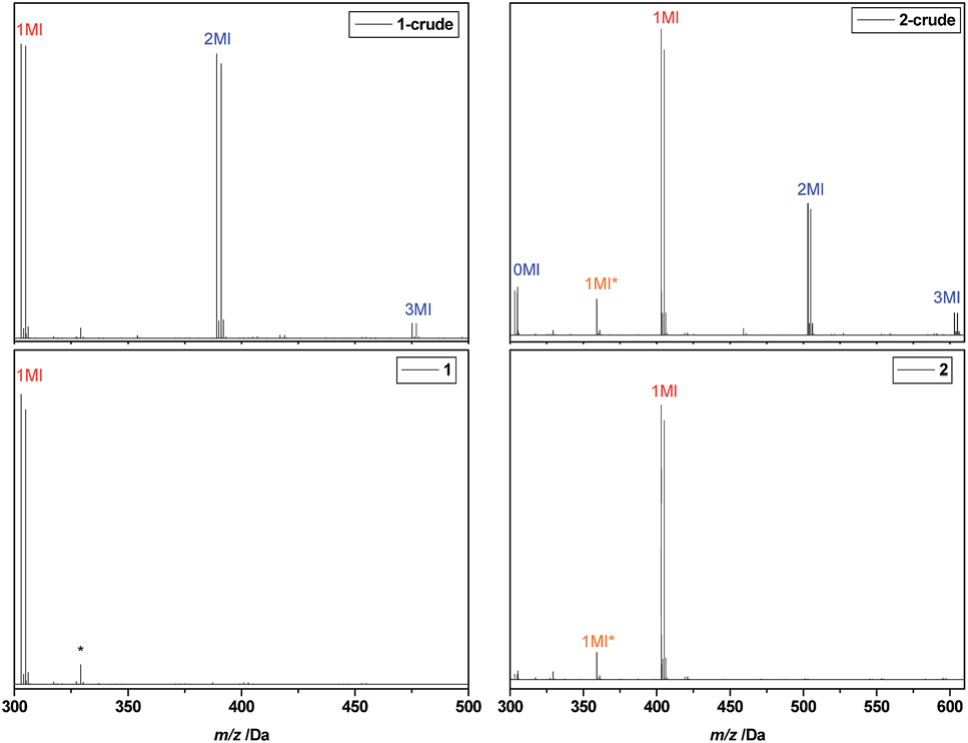
ESI-MS spectra of I-MA-Br **1** (left) and I-MA-EA-Br **2** (right), before (top) and after (bottom) purification. The MI* peak corresponds to a SUMI-Cl product resulting from halogen exchange. The peak marked with a sole asterisk results from a background signal.

After reaction, the product mixtures were extracted with chloroform to remove copper and DMSO and subsequently analysed *via* ESI-MS. The spectrum of the crude product **1** is depicted in Fig. 2 (top left). A number of different insertion products are observed, demonstrating the statistical nature of the radical insertion reaction. Next to the desired product, in which only one MA monomer unit was inserted (1MI), also side products containing either no (0MI) or more than one inserted monomer (2MI, 3MI) are well visible (compare Scheme 1 for notation of products). It must be noted that the ESI-MS spectra of the crude product mixtures can only interpreted qualitatively due to mass and ionization bias effects for different oligomers. In a previous study,^33^ we have designed proper calibration files to translate ionization potentials of the different SUMI oligomers into their true molar ratios in a mixture. In that study we demonstrated that an ionization bias exists for the first 2–3 monomer insertions (3MI > 2MI > 1MI > 0MI), but from the fourth insertion on, the bias effect is more of less levelled out. Anyhow, in order to isolate the desired SUMI product, the crude reaction mixtures need to be purified.

Depending on the type of monomer and the amount of crude material to purify, either classical column chromatography (SiO_2_, hexane : ethylacetate 4 : 1) or preparative recycling SEC was used for this purpose. When MA, EA, EGMEA or DEGEEA is used in a SUMI reaction, the resulting reaction mixture can be purified by column chromatography based on polarity. These monomers exhibit enough polarity differences so that an oligomer with one monomer insertion (1MI) elutes slower than the 0MI oligomer, but faster than the 2MI and 3MI oligomers. The opposite is true when EHA is inserted. In this case the apolar side chain overrules the polar ester functionality, overall giving this monomer an apolar character. Also in this case, column chromatography can be used whereby 2MI elutes first, followed by elution of 1MI and finally elution of 0MI. Only in the case when BuA is used for SUMI reaction, the final oligomer mixture cannot be purified by chromatography based on polarity. In this case, the apolar butyl aside chain and the polar ester function level each other out, and no different retention times are observed for the different insertion products. Therefore, product mixtures resulting from BuA insertion (**7**, **9**, **14** and **15**) always had to be purified *via* rec-SEC. With this automated technique, molecules are repeatedly recycled over a number of SEC columns, until molecules with different sizes (hydrodynamic volumes) are completely separated. The higher the number of inserted monomers, the more cycles are needed to purify the oligomers. Rec-SEC was also always used when the amount of material to purify was less than 600 mg. In this case less material loss was obtained compared to when a classical column was applied.

**Fig. 3:**
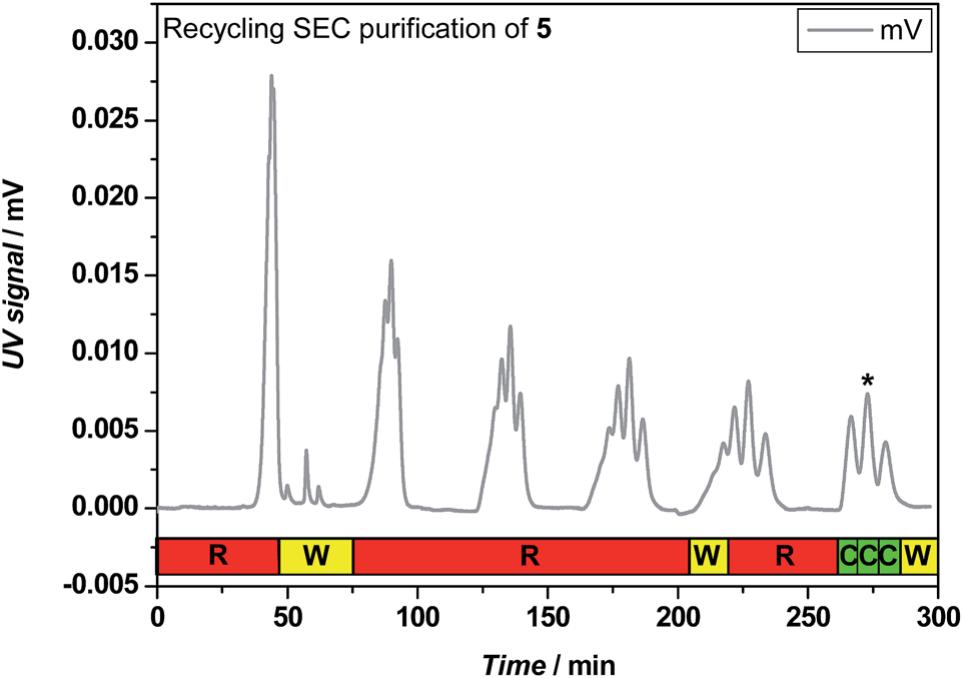
Recycling SEC trace recorded during consecutive purification cycles of **5**, with letters R, W and C standing for recycle, waste and collect. The peak of the desired oligomer is marked with an asterisk.

An example of the rec-SEC trace recorded during purification of I-MA-EA-EGMEA-DEGEEA-EHA **5** is depicted in Fig. 3. Only after 6 cycles, the oligomers consisting of 4 (0MI), 5 (1MI), 6 (2MI) and 7 (3MI) monomer units are separated well enough from each other to fractionate them and obtain the pure SD pentamer **5**. Of course, due to the statistical nature of the radical insertion process (which exhibits also a chain length dependent propagation),^33^ after each SUMI step, a variety of SUMI products are formed. Although the aim of this research project is to synthesize the desired ABCDE-type pentamers, also the other oligomers formed in each SUMI step can perfectly be isolated and used in additional SUMI reactions as well. In order to encode certain information by tuning the monomer sequence, an isolated ABCCD oligomer for instance may be equally important as an ABCDE sequence. In that respect, when evaluating the efficiency of a SUMI process, one has to take in to account the overall yield of a SUMI reaction as well as the individual yields of all other isolated acrylate oligomers. Full information on the reaction yields can be found in Table S1, in the ESI.† Nevertheless, higher SUMI products are more difficult to separate, since the hydrodynamic volume changes become smaller with increasing chain length. The isolated yields of the desired SUMIs therefore decrease after each insertion step (see Table 1) and for the final ABCDE-type SD pentamers **5** and **19**, only a few mg of purified material could be retained. Still, synthesis of gram-scale of these materials is feasible by translating the batch into a continuous flow process. We had demonstrated previously that SUMI reactions can be scaled up by a factor 100 by employing a mesoscale flow reactor, without any efficiency penalty with regards to the obtained SUMI oligomers.^33^ The current photo-induced SUMI CMP reactions can likewise be translated to a flow process.^36^ When performed in flow,^34^ the reaction can then also be followed by online ESI-MS characterization to further optimize reaction conditions. This will be subject of a follow-up study.

After purification, the SD oligomers are again fully characterized by means of ESI-MS and NMR (see ESI†). For I-MA-Br **1** and I-MA-EA-Br **2**, the ESI-MS spectra before and after purification are depicted in Fig. 2. For oligomer **1**, purification yields a 100% pure I-MA-Br structure, which is then used for synthesis of oligomer **2**. The ESI-MS spectrum of the crude mixture **2** gives in this case not only the Br-end capped 0MI, 1MI, 2MI and 3MI oligomers, but also a second peak series denoted with MI*. Closer analysis of the *m*/*z* values revealed that these peak series correspond to Cl-end capped oligomers. Apparently an undesired halogen exchange side reaction takes place during the second SUMI reaction, resulting in a certain percentage of Cl-end capped oligomers. Purification of the desired bromine oligomer from the chlorine side product fails due to differences in polarity or molar mass being too small. Furthermore, these chlorine by-products are formed to various degrees in some other SUMI reactions as well (see Table 1). Further evidence of the halogen exchange was found in the ^13^C APT NMR spectra of the purified compounds, where the CH–Cl gives rise to negative resonances at 55 and 56 ppm. At first, no explanation for the origin of this halogen exchange could be found since in none of the cases any chloride ions were present in the reactions. However, careful investigation revealed that after purification of the first SUMI product, oligomer **1** still contained a few percent of residual chloroform (solvent used for rec-SEC). It is known from literature that when chloroform is exposed to UV light, it photo-catalytically degrades into CHCl_2_ radicals and free chlorine radicals.^42^ We believe that these Cl radicals during the photoCMP interfere and exchange some of the Br radicals that are normally generated in the proposed photoCMP mechanism.^43^ In order to avoid this halogen exchange as much as possible, all purified SD oligomers were carefully dried from residual chloroform traces by evaporation on a high vacuum pump overnight. After this procedure, formation of Cl-end capped oligomers during the following SUMI reaction could be avoided or limited to a minor amount. Although the chlorine-capped products are not easily separated, their presence is largely irrelevant for the further insertion reactions, as chlorine-terminated materials are inactive in the subsequent photoCMP reactions and hence do not insert any further monomer unit. However, due to this effect, maximum achievable product yields of subsequent reactions are limited and hence it is desirable to avoid the formation of Cl-end capped products in general.

**Fig. 4:**
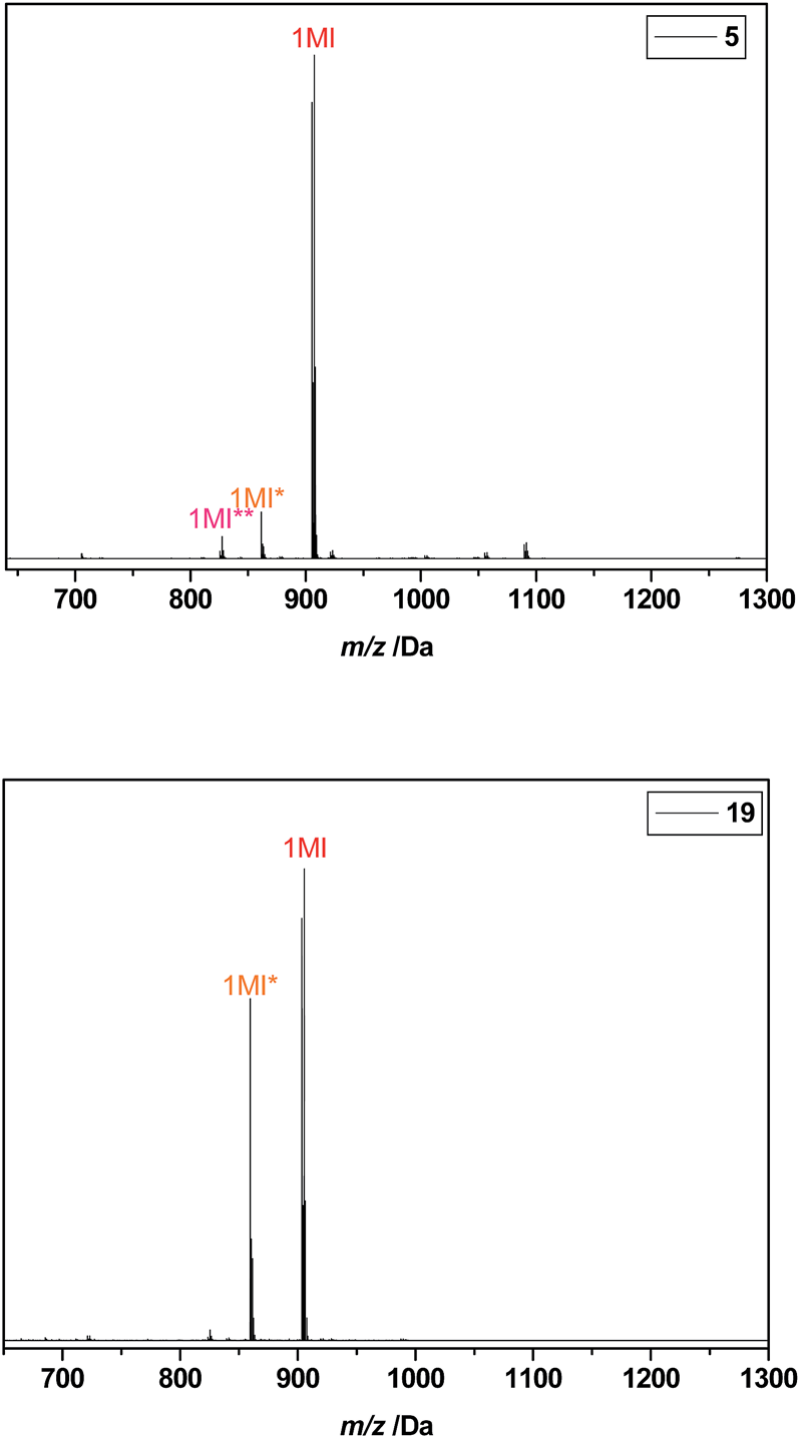
ESI-MS spectra of purified pentamers **5** (top) and **19** (bottom). The MI* peak correspond to a SUMI-Cl product resulting from halogen exchange, and the MI** peak results from disproportionation (dead chain).

To display the difference in product outcome with and without optimization of the reaction protocol by intermediate evaporation of chloroform leftovers, the ESI-MS spectra of the final purified pentamers I-MA-EA-EGMEA-DEGEEA-EHA **5** (with optimization) and I-BuA-MA-EA-EHA-DEGEEA **19** (without optimization) are depicted in Fig. 4. In here, considerable amounts of Cl-end capped product are observed for pentamer **19**, which synthesis was not optimized by removing chlorinated solvent leftovers in each intermediate SUMI reaction. On the other hand, the ESI-MS of the optimized and purified SD pentamer **5** mainly displays the mono-disperse Br-end capped oligomer and only contains a few percentages of Cl-end capped by-product together with a very small amount of H-terminated (dead) oligomers. This result clearly proves that intermediate evaporation of chloroform leftovers between each subsequent SUMI reaction, substantially improves the final product outcome.

To assign the main peak in all ESI-MS spectra, a comparison between the theoretical mass-to-charge (*m*/*z*) value and the experimentally observed *m*/*z* value is listed in Table 2. In all cases, an almost perfect match between the 2 values was observed, and the main peak always showed the typical isotopic pattern of a Br-substituted compound. This proves unambiguously that the peaks can be assignment to their corresponding Br-end capped SD oligomers.

**Table 2:** Table of masses for purified SUMI-Br oligomers **1–19**, observed in the mass spectra

SUMI product, single Na^+^ charged	*m*/*z*^theor.^/Da	*m*/*z*^exp.^/Da	Δ*m*/*z*/Da
I-MA-Br (**1**)	303.02	303.02	0.00
I-MA-EA-Br (**2**)	403.07	403.07	0.00
I-MA-EA-EGMEA-Br (**3**)	533.13	533.14	0.01
I-MA-EA-EGMEA-DEGEEA-Br (**4**)	721.24	721.24	0.00
I-MA-EA-EGMEA-DEGEEA-EHA-Br (**5**)	905.38	905.39	0.01
I-MA-EHA-Br (**6**)	487.17	487.17	0.00
I-MA-EHA-BuA-Br (**7**)	615.25	615.25	0.00
I-MA-EHA-BuA-DEGEEA-Br (**8**)	803.35	803.36	0.00
I-MA-BuA-Br (**9**)	431.10	431.10	0.00
I-MA-BuA-EHA-Br (**10**)	615.25	615.25	0.00
I-MA-BuA-EHA-DEGEEA-Br (**11**)	803.35	803.36	0.01
I-MA-EGMEA-Br (**12**)	433.08	433.08	0.00
I-MA-EGMEA-DEGEEA-Br (**13**)	621.19	621.19	0.00
I-MA-EGMEA-DEGEEA-BuA-Br (**14**)	749.27	749.27	0.00
I-BuA-Br (**15**)	345.07	345.07	0.00
I-BuA-MA-Br (**16**)	431.10	431.10	0.00
I-BuA-MA-EA-Br (**17**)	531.16	531.16	0.00
I-BuA-MA-EA-EHA-Br (**18**)	715.30	715.30	0.00
I-BuA-MA-EA-EHA-DEGEEA-Br (**19**)	903.41	903.41	0.00

**Fig. 5:**
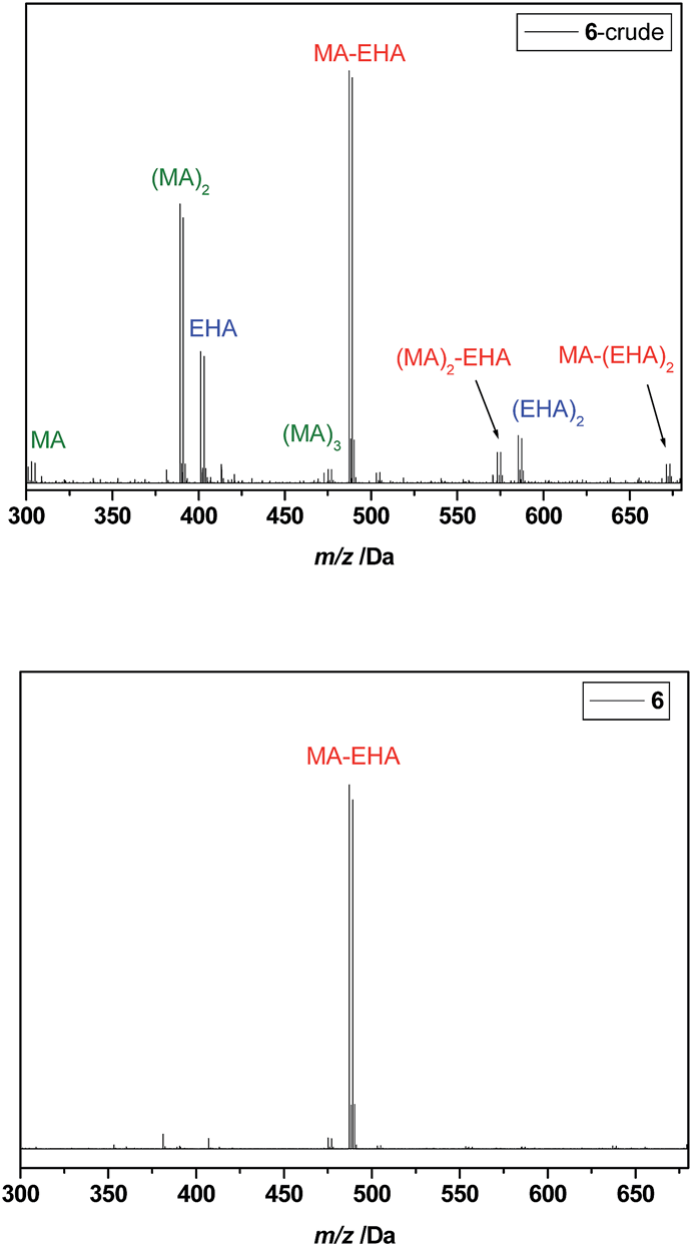
ESI-MS spectra of I-MA-EHA-Br **6**, before and after purification. For synthesis of **6**, MA and EHA were inserted subsequently, without intermediate purification, so both homo-oligomers as well as MA-EHA *block-co*-oligomers are observed in the crude product.

Since product separation poses the largest hurdle with respect to reaction yields, further tests were carried out to reduce the number of necessary product isolations. Oligomer **6** was prepared by inserting first MA into the initiator (SUMI-1) in a first radical insertion reaction, directly followed by insertion of EHA in a second reaction without any product isolation in between. Conversion of MA, followed by *in situ* FT-IR, was close to 100% before the second monomer EHA was added. This synthetic strategy is used preferentially to obtain multiblock-copolymers, as described in literature.^28^,^29^ The ESI-MS spectrum of crude oligomer **6** is depicted in Fig. 5, top. A number of different peaks is observed, corresponding to very short MA-EHA *block*-oligomers, but also to both MA and EHA homo-oligomers. This clearly demonstrates that besides the desired SC *block*-oligomer structure, also certain amounts of homo-oligomers are formed in which no sequence is present. Rec-SEC purification allows still to isolate the desired SUMI product I-MA-EHA-Br **6**, as shown in Fig. 5, bottom. Yet, the yield after the comparatively tedious separation is 18%, which is no improvement compared to the combined yield of 20% when both SUMIs are carried out independently with intermediate isolation. It can thus be concluded that intermediate purifications are indeed highly recommended in order to obtain true monodisperse SD oligomers in the most efficient way as the desired product content in the crude mixture must be maximized in order to allow for efficient separation.

## Conclusions

We have used photoCMP as a useful technique to synthesize a library of monodisperse sequence defined acrylate oligomers *via* sequential single monomer unit insertion reactions. Oligomers with a sequence of up to 5 monomer units were obtained this way, which marks the current record in SD oligomers made *via* any CRP technology. In order to avoid radical coupling, monomer conversions were followed *in situ* by on-line FT-IR analysis and reactions were stopped at reaching 80–90% conversion. To overcome the statistical nature of the radical insertion process, reaction mixtures had to be purified in between each SUMI step by means of column chromatography or rec-SEC. The chosen purification method depends on the type of inserted monomer and the amount of material to be purified. As a result of reaction statistics and elaborate purification, isolated reaction yields for the desired SUMI oligomer decrease for each additional SUMI step. However, it must be noted that after every SUMI reaction, also the other oligomers (with 0 or more than 1 monomers inserted) can be isolated and used for further SUMI reactions, if desirable. Generally, product separation is the main limiting factor for any SUMI process. After purification, all SD oligomers were carefully characterized by means of NMR and ESI-MS. Besides the desired Br end capped SD oligomers, in some cases also Cl-end capped species were observed, which resulted from a halogen exchange reaction occurring during photo-induced SUMI when residual chloroform was present in the reaction mixtures. Careful evaporation of all residual solvent before the next SUMI reaction was started, avoided this undesired halogen exchange reaction or reduced it to a minimum. Compared to RAFT-based SUMI reactions, photoCMP yields comparable reaction yields and is equally efficient in synthesis. Both methods seem hence equally capable for SUMI synthesis and no recommendation to use one over the other should be easily made. Yet, photoCMP is conveniently carried out at room temperature, and hence avoids radical transfer-related side product formation. In principle also with RAFT milder reaction conditions can be achieved, however, only with significant efforts. In this respect photoCMP is superior, which further underpins the very high potential of this type of reactions in the field of controlled polymerization. Nevertheless, RAFT and photoCMP should be seen as complimentary, as different endgroups can also be understood as additional functions in the chain sequence. Hence a choice between thioesters and halogen terminated chains only increases the synthetic possibilities for further endeavours into the synthesis of higher SUMI products with chain lengths beyond 5.

## Supplementary Material

Supplementary information
