# Supplementary material for: Synthesis of sequence-defined acrylate oligomers via photo-induced copper-mediated radical monomer insertions
Source: Chem Sci. 2015 Jul 3;6(10):5753–61. doi: 10.1039/c5sc02035b (PMC5947509; doi:10.1039/c5sc02035b)
Supplement: Supplementary file 1 [file SC-006-C5SC02035B-s001.pdf]

Electronic Supplementary Material (ESI) for Chemical Science.
This journal is © The Royal Society of Chemistry 2015

Supporting Information

Synthesis of Sequence-Defined Acrylate Oligomers via Photoinduced Copper-Mediated Radical Monomer Insertions

*Joke Vandenbergh,[a] Gunther Reekmans,[b] Peter Adriaensens[b,c] and Thomas Junkers[a,c]**

[a]Polymer Reaction Design Group, Institute for Materials Research (IMO-IMOMEC), Universiteit Hasselt, Agoralaan Building D, B-3590 Diepenbeek, Belgium

[b]Nuclear Magnetic Resonance Spectroscopy Group, Institute for Materials Research (IMO-IMOMEC), Universiteit Hasselt, Agoralaan Building D, B-3590 Diepenbeek, Belgium

[c]IMEC associated lab IMOMEC, Wetenschapspark 1, B-3590 Diepenbeek, Belgium

Experimental Section: Table of Contents

1. Materials
2. Characterization
3. Synthetic Procedures
4. Spectroscopic Data

1. Materials

The monomers methyl acrylate (MA, Acros, 99%), ethyl acrylate (EA, Acros, 99.5%), ethylene glycol methyl ether acrylate (EGMEA, Acros, 98%), *n*-butyl acrylate (BuA, Acros, 99%), 2-ethylhexyl acrylate (EHA, Acros, 99%) and di(ethylene glycol) ethyl ether acrylate (DEGEEA, Sigma-Aldrich, 90%) were deinhibited over a column of activated basic alumina, prior to use. Ethyl 2-bromoisobutyrate (EBiB) and Copper (II) bromide (CuBr2) were used as received. Tris[2-(dimethylamino)ethyl]amine (ME6TREN) was synthesized according to a previously reported literature.[1] All solvents used are obtained from commercial sources (Acros and Sigma-Aldrich) and used without further purification.

2. Characterization

An OMNICURE Series 1000 system was used as UV-light source. The OMNICURE system was equipped with a 100 W high pressure mercury vapor short arc lamp (320–500 nm) at an iris setting of 100 %. In-Situ FTIR Spectroscopy (TM15, Mettler Toledo) was used to monitor monomer conversion by measuring the area of acrylate peaks. Purification of products was performed either via classical column chromatography (SiO_2, Hexane:EtOAc 4:1) or on a recycling preparative HPLC LC-9210 NEXT system in the manual injection mode (3 mL) comprising a JAIGEL-1H and JAIGEL-2H column and a NEXT series UV detector using $CHCl_3$ as the eluent with a flow rate of 3.5 $mL \cdot min^{-1}$. Fractions were collected manually. NMR spectra were recorded in deuterated chloroform with a Varian Inova 300 or 400 spectrometer at 300 or 400 MHz for ^1H NMR and at 75 or 100 MHz for ^{13}C NMR using a Varian probe (5 mm-4-nucleus AutoSWPFG) and a pulse delay of 12 s for the proton spectra and 20 s for the carbon spectra. Chemical shifts (δ) are reported in ppm relative to the residual $CHCl_3$ proton signal at δ = 7.24 ppm for ^1H NMR or $CDCl_3$ carbon signal at δ = 77.7 for ^{13}C NMR. A higher number of chemical shifts than carbon atoms present were often reported for

the ^{13}C NMR spectra, since the products comprise a mixture of different diastereomers. ESI-MS was performed using an LCQ Fleet mass spectrometer (ThermoFischer Scientific) equipped with an atmospheric pressure ionization source operating in the nebulizer assisted electro spray mode. The instrument was calibrated in the m/z range 220-2000 using a standard solution containing caffeine, MRFA and Ultramark 1621. A constant spray voltage of 5 kV was used and nitrogen at a dimensionless auxiliary gas flow-rate of 3 and a dimensionless sheath gas flow-rate of 3 were applied. The capillary voltage, the tube lens offset voltage and the capillary temperature were set to 25 V, 120 V, and 275°C respectively. A 250 µL aliquot of a polymer solution with concentration of 10 µg mL^{-1} was injected. A mixture of THF and methanol (THF:MeOH = 3:2), all HPLC grade, was used as solvent.

3. Synthetic Procedures

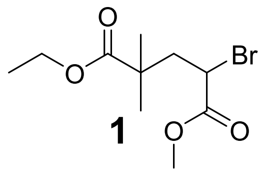

I-MA-Br (1). In a typical procedure, 23.231 mmol (2.000 g, 1 equiv) of the monomer MA, 23.231 mmol (4.540 g, 1 equiv) of EBiB initiator, 0.325 mmol (0.072 g, 0.014 equiv) of $CuBr_2$, 1.951 mmol (0.450 g, 0.084 equiv) of ME6TREN and 6 mL of DMSO (50 vol%) were added into a sealed 3-neck flask. The flask was connected to a UV source, an IR probe and sealed with a septum (see Figure S1). The flask was kept in the dark and purged with nitrogen for 15 min after which the UV source was switched on. The flask was cooled in a cold water bath to keep the temperature at RT. Monomer insertion was conducted under UV and the monomer conversion was monitored by online FT-IR spectroscopy. When the monomer conversion was ≥90% (based on the FT-IR signal), the reaction was stopped by switching of the UV light. The flask was opened and an NMR sample was taken to determine conversion.

The solution was diluted with deionized water and extracted with chloroform. The organic phase was evaporated to yield the crude SUMI product which was purified with column chromatography (SiO_2, hexane/EtOAc 4/1) to yield 2.8 g (43%) of pure **I-MA-Br (1)**. ^1H NMR (400 MHz, $CDCl_3$) δ 4.27 (dd, *J* = 8.8, 4.9 Hz, 1H), 4.07 (q, *J* = 7.1 Hz, 2H), 3.72 (s, 3H), 2.50 (dd, *J* = 14.7, 8.8 Hz, 1H), 2.27 (dd, *J* = 14.7, 4.9 Hz, 1H), 1.21 (t, *J* = 7.1 Hz, 3H), 1.19 (s, 3H), 1.12 (s, 3H). ^{13}C NMR (100 MHz, $CDCl_3$) δ 176.98, 171.22, 61.49, 53.60, 45.40, 42.89, 41.74, 26.44, 25.06, 14.68. ESI-MS (m/z): 303.02 ($M+Na^+$).

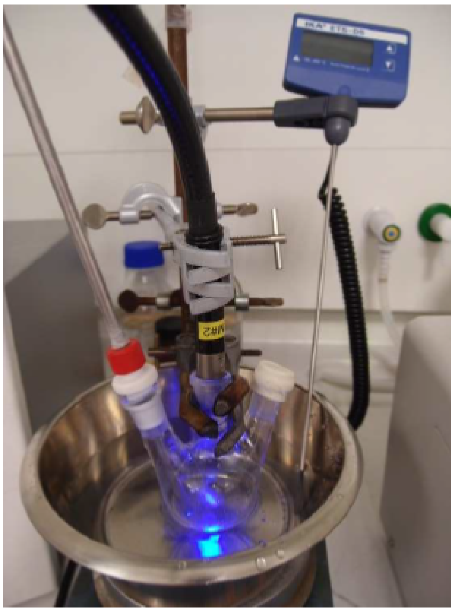

Figure S1. Reaction set-up used for the photo-induced copper-mediated radical SUMI reaction.

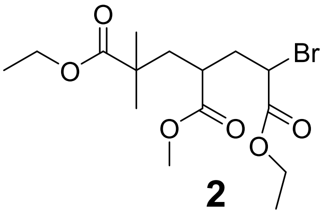

2

I-MA-EA-Br (2). The same procedure as for synthesis of (**1**) was applied, using 9.988 mmol (1.000 g, 1 equiv) of the monomer EA, 9.988 mmol (2.800 g, 1 equiv) of macroinitiator **1**, 0.140 mmol (0.031 g, 0.014 equiv) of $CuBr_2$, 0.837 mmol (0.193 g, 0.084 equiv) of ME6TREN and 4 mL of DMSO (50 vol%). After reaction, the crude SUMI product was

purified with column chromatography (SiO_2, hexane/EtOAc 4/1) to yield 1.795 g (47%) of pure **I-MA-EA-Br (2)**. ^{1}H NMR (400 MHz, $CDCl_3$) δ 4.21 – 3.98 (m, 5H), 3.60 (ds, *J* = 4.0 Hz, 3H), 2.71 – 2.40 (m, 1H), 2.32 – 1.93 (m, 3H), 1.69 – 1.55 (m, 1H), 1.27 – 1.15 (m, 6H), 1.12 (ds, *J* = 7.8 Hz, 3H), 1.07 (ds, *J* = 9.4 Hz, 3H), mixture of isomers 55%/45%. ^{13}C NMR (100 MHz, $CDCl_3$) δ 177.53, 177.50, 175.93, 169.78, 169.70, 62.76, 62.66, 61.26, 61.21, 52.49, 44.46, 42.97, 42.89, 42.51, 42.46, 42.39, 40.88, 40.65, 39.37, 38.85, 26.35, 26.30, 25.39, 25.23, 14.69, 14.66, 14.55, 14.46. ESI-MS (m/z): 403.07 ($M+Na^+$).

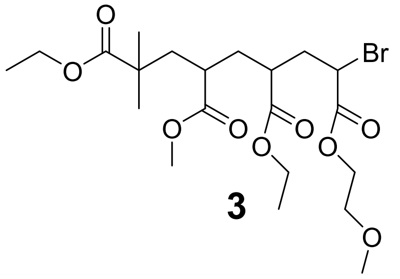

3

I-MA-EA-EGMEA-Br (3). The same procedure as for synthesis of (**1**) was applied, using 4.710 mmol (0.613 g, 1 equiv) of the monomer EGMEA, 4.710 mmol (1.795 g, 1 equiv) of macroinitiator **2**, 0.066 mmol (0.015 g, 0.014 equiv) of $CuBr_2$, 0.395 mmol (0.091 g, 0.084 equiv) of ME6TREN and 3 mL of DMSO (67 vol%). After reaction, the crude SUMI product was purified with column chromatography (SiO_2, hexane/EtOAc 4/1) to yield 0.500 g (21%) of pure **I-MA-EA-EGMEA-Br (3)**. ^{1}H NMR (400 MHz, $CDCl_3$) δ 4.30 – 4.18 (m, 3H), 4.16 – 3.99 (m, 4H), 3.63 – 3.58 (m, 3H), 3.58 – 3.54 (m, 2H), 3.33 (s, 3H), 2.66 – 1.83 (m, 5H), 1.76 – 1.41 (m, 3H), 1.26 – 1.16 (m, 6H), 1.12 – 1.05 (m, 6H). ^{13}C NMR (100 MHz, $CDCl_3$) δ 177.63, 176.55, 176.52, 176.49, 176.39, 174.47, 170.00, 169.93, 169.83, 169.80, 70.59, 65.61, 65.57, 61.58, 61.51, 61.14, 59.63, 59.59, 52.37, 52.34, 52.27, 44.49, 44.15, 43.52, 43.43, 43.02, 42.88, 42.74, 42.67, 42.45, 41.98, 41.90, 41.69, 41.56, 40.71, 40.64, 40.42, 40.37, 37.98, 37.55, 37.38, 37.21, 37.12, 36.57, 36.47, 36.41, 26.25, 26.20, 26.07, 26.05, 25.65, 25.61, 25.42, 14.77, 14.73, 14.70. ESI-MS (m/z): 533.13 ($M+Na^+$).

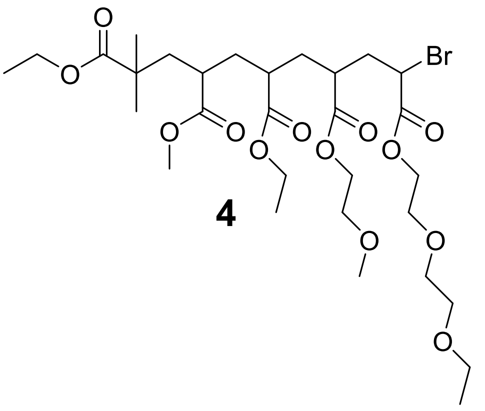

4

I-MA-EA-EGMEA-DEGEEA-Br (4). The same procedure as for synthesis of (**1**) was applied, using 0.978 mmol (0.184 g, 1 equiv) of the monomer DEGEEA, 0.978 mmol (0.500 g, 1 equiv) of macroinitiator **3**, 0.014 mmol (0.003 g, 0.014 equiv) of $CuBr_2$, 0.084 mmol (0.019 g, 0.084 equiv) of ME6TREN and 0.6 mL of DMSO (50 vol%). After reaction, the crude SUMI product was purified via recycling SEC to yield 0.125 g (18%) of pure **I-MA-EA-EGMEA-DEGEEA-Br (4)**. ^{1}H NMR (300 MHz, $CDCl_3$) δ 4.36 – 3.93 (m, 9H), 3.64 (t, *J* = 4.7 Hz, 2H), 3.60 – 3.54 (m, 5H), 3.44 (q, *J* = 7.1 Hz, 2H), 3.29 (s, 3H), 2.74 – 2.39 (m, 1H), 2.39 – 1.78 (m, 6H), 1.78 – 1.32 (m, 4H), 1.23 – 1.01 (m, 15H). ^{13}C NMR (75 MHz, $CDCl_3$) δ 177.59, 176.56, 176.50, 175.03, 174.95, 174.45, 174.35, 174.24, 169.98, 169.92, 169.77, 71.25, 70.76, 70.34, 69.22, 67.20, 65.62, 64.12, 61.33, 61.20, 61.04, 59.36, 52.23, 52.14, 44.44, 44.28, 43.63, 43.45, 42.81, 42.61, 42.52, 42.35, 41.96, 41.87, 41.69, 41.54, 41.42, 40.65, 40.58, 40.38, 38.01, 37.86, 37.60, 37.47, 37.31, 37.12, 36.88, 36.66, 36.38, 35.78, 35.70, 35.19, 35.03, 34.67, 34.40, 34.21, 26.00, 25.41, 15.70, 14.72, 14.64. ESI-MS (m/z): 721.24 (M+Na^+).

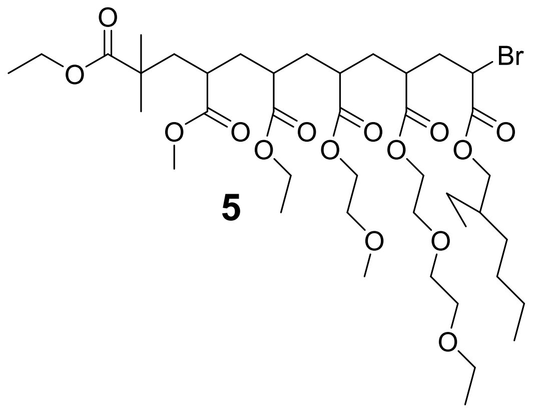

5

I-MA-EA-EGMEA-DEGEEA-EHA-Br (5). The same procedure as for synthesis of (**1**) was applied, using 0.179 mmol (0.033 g, 1 equiv) of the monomer EHA, 0.179 mmol (0.125 g, 1 equiv) of macroinitiator **4**, 0.0025 mmol (0.600 mg, 0.014 equiv) of $CuBr_2$, 0.015 mmol (3.500 mg, 0.084 equiv) of ME6TREN and 0.6 mL of DMSO (80 vol%). After reaction, the crude SUMI product was purified via recycling SEC to yield 0.016 g (10%) of pure **I-MA-EA-EGMEA-DEGEEA-EHA-Br (5)**. ^{1}H NMR (300 MHz, $CDCl_3$) δ 4.38 – 3.91 (m, 11H), 3.74 – 3.44 (m, 13H), 3.35 (s, 3H), 2.80 – 1.43 (m, 15H), 1.43 – 1.04 (m, 23H), 0.87 (t, J = 7.3 Hz, 6H). ^{13}C NMR (75 MHz, $CDCl_3$) δ 177.66, 176.57, 175.04, 174.41, 169.96, 71.19, 70.83, 70.41, 69.48, 68.88, 67.27, 64.33, 64.08, 61.10, 59.43, 52.27, 52.16, 44.91, 44.72, 43.70, 43.50, 42.98, 42.79, 42.38, 41.70, 40.59, 40.39, 39.25, 37.66, 37.38, 37.17, 36.67, 35.45, 35.10, 30.72, 29.40, 25.97, 25.56, 24.15, 23.52, 15.77, 14.66, 11.49. ESI-MS (m/z): 905.38 (M+Na$^+$).

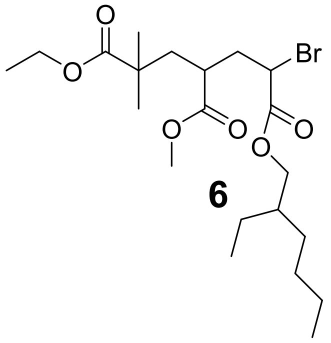

6

I-MA-EHA-Br (6). In this specific in-situ procedure, first 11.615 mmol (1.000 g, 1 equiv) of the first monomer MA, 11.615 mmol (2.270 g, 1 equiv) of EBiB initiator, 0.163 mmol (0.036 g, 0.014 equiv) of $CuBr_2$, 0.976 mmol (0.255 g, 0.084 equiv) of ME6TREN and 3 mL of DMSO (50 vol%) were added into the sealed 3-neck flask and after purging the reaction was executed. When the monomer conversion was ≥90% (based on the FT-IR signal), the reaction was pauzed by switching of the UV light. Subsequently, 11.615 mmol (2.140 g, 1 equiv) of the second monomer EHA and 2.4 mL of DMSO were added with a purged syringe and the 2nd insertion reaction was started by switching the light back on. After reaction, the crude SUMI product was purified via recycling SEC to yield 1.000 g (18%) of pure **I-MA-EHA-Br (6)**. ^{1}H NMR (400 MHz, $CDCl_3$) δ 4.19 – 3.94 (m, 5H), 3.64 (ds, *J* = 5.4 Hz, 3H), 2.76 – 2.45 (m, 1H), 2.37 – 1.96 (m, 3H), 1.71 – 1.52 (m, 2H), 1.40 – 1.19 (m, 11H), 1.16 (ds, *J* = 8.6 Hz, 3H), 1.11 (ds, *J* = 9.3 Hz, 3H), 0.87 (dt, *J* = 7.4, 1.8 Hz, 6H), mixture of isomers 51%/49%. ^{13}C NMR (100 MHz, $CDCl_3$) δ 177.64, 176.04, 170.05, 69.01, 61.36, 61.31, 52.55, 44.65, 43.10, 43.01, 42.63, 42.54, 41.02, 40.77, 39.54, 39.38, 39.02, 30.87, 29.51, 26.41, 26.32, 25.54, 25.41, 24.28, 23.59, 14.79, 14.75, 14.70, 11.59. ESI-MS (m/z): 487.17 ($M+Na^+$).

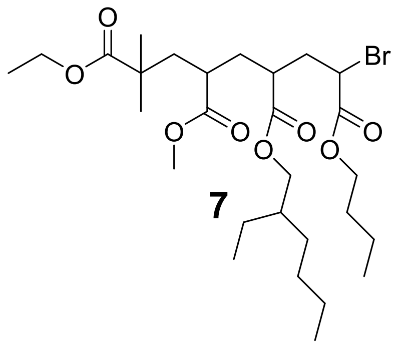

7

I-MA-EHA-BuA-Br (7). The same procedure as for synthesis of (**1**) was applied, using 1.611 mmol (0.207 g, 1 equiv) of the monomer BuA, 1.611 mmol (0.75 g, 1 equiv) of macroinitiator **6**, 0.023 mmol (0.005 g, 0.014 equiv) of $CuBr_2$, 0.135 mmol (0.032 g, 0.084 equiv) of ME6TREN and 3.4 mL of DMSO (80 vol%). After reaction, the crude SUMI product was purified via recycling SEC to yield 0.250 g (27%) of pure **I-MA-EHA-BuA-Br (7)**. ^{1}H NMR (400 MHz, $CDCl_3$) δ 4.26 – 3.87 (m, 7H), 3.62 – 3.58 (m, 3H), 2.68 – 1.85 (m, 6H), 1.79 – 1.43 (m, 5H), 1.43 – 1.17 (m, 13H), 1.12 (s, 3H), 1.09 (s, 3H), 0.94 – 0.80 (m, 9H). ^{13}C NMR (100 MHz, $CDCl_3$) δ 177.65, 176.53, 176.42, 174.77, 174.71, 174.58, 170.04, 169.95, 169.86, 67.98, 67.95, 67.89, 67.75, 66.65, 66.55, 61.17, 52.38, 52.35, 52.26, 44.95, 44.63, 43.57, 43.44, 43.17, 43.06, 42.91, 42.76, 42.48, 42.41, 42.30, 42.19, 41.80, 41.69, 40.77, 40.71, 40.43, 39.31, 38.24, 37.68, 37.55, 37.31, 37.12, 36.79, 36.56, 36.50, 30.99, 30.90, 29.52, 26.23, 26.14, 25.96, 25.93, 25.77, 25.74, 25.55, 25.47, 24.37, 24.30, 23.58, 19.61, 14.69, 14.29, 11.58. ESI-MS (m/z): 615.25 (M+Na^{+}).

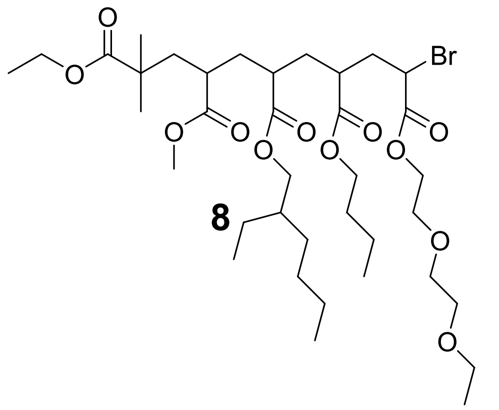

8

I-MA-EHA-BuA-DEGEEA-Br (8). The same procedure as for synthesis of (**1**) was applied, using 0.420 mmol (0.079 g, 1 equiv) of the monomer DEGEEA, 0.420 mmol (0.250 g, 1 equiv) of macroinitiator **7**, 0.006 mmol (1.320 mg, 0.014 equiv) of $CuBr_2$, 0.035 mmol (8.150 mg, 0.084 equiv) of ME6TREN and 0.6 mL of DMSO (70 vol%). After reaction, the crude SUMI product was purified via recycling SEC to yield 0.040 g (12%) of pure **I-MA-EHA-BuA-DEGEEA-Br (8)**. ^{1}H NMR (300 MHz, $CDCl_3$) δ 4.38 – 4.15 (m, 3H), 4.15 – 3.85 (m, 6H), 3.69 (t, J = 4.9 Hz, 2H), 3.65 – 3.53 (m, 7H), 3.49 (q, J = 7.0 Hz, 2H), 2.73 – 1.86 (m, 7H), 1.84 – 1.45 (m, 7H), 1.42 – 1.14 (m, 16H), 1.12 (s, 3H), 1.09 (s, 3H), 0.95 – 0.81 (m, 9H). ^{13}C NMR (75 MHz, $CDCl_3$) δ 177.69, 176.66, 176.56, 175.45, 175.29, 175.22, 175.03, 174.66, 169.96, 169.81, 71.40, 70.46, 69.34, 67.88, 67.73, 67.34, 65.78, 65.48, 61.16, 52.38, 52.32, 52.18, 44.43, 43.77, 43.65, 43.01, 42.89, 42.70, 42.44, 42.05, 41.84, 41.57, 40.84, 40.75, 40.48, 39.33, 37.61, 37.36, 31.21, 30.94, 29.56, 25.99, 25.84, 25.77, 25.57, 24.28, 23.61, 19.75, 15.81, 14.74, 14.37, 11.57. ESI-MS (m/z): 803.35 ($M+Na^+$).

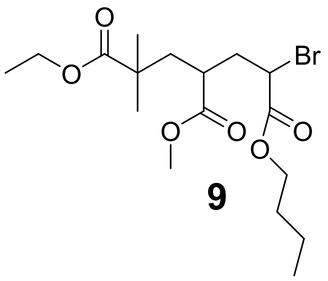

9

I-MA-BuA-Br (9). The same procedure as for synthesis of (**1**) was applied, using 2.840 mmol (0.365 g, 1 equiv) of the monomer BuA, 2.840 mmol (0.800 g, 1 equiv) of macroinitiator **1**, 0.040 mmol (0.009 g, 0.014 equiv) of $CuBr_2$, 0.240 mmol (0.055 g, 0.084 equiv) of ME6TREN and 4.3 mL of DMSO (80 vol%). After reaction, the crude SUMI product was purified via recycling SEC to yield 0.5 g (43%) of pure **I-MA-BuA-Br (9)**. ^{1}H NMR (400 MHz, $CDCl_3$) δ 4.20 – 3.98 (m, 5H), 3.63 (ds, J = 4.6 Hz, 3H), 2.72 – 2.43 (m, 1H), 2.35 – 1.97 (m, 3H), 1.72 – 1.56 (m, 3H), 1.43 – 1.30 (m, 2H), 1.22 (t, J = 7.1 Hz, 3H), 1.15 (ds, J = 8.2 Hz, 3H), 1.11 (ds, J = 9.3 Hz, 3H), 0.90 (dt, J = 7.4, 2.1 Hz, 3H), mixture of isomers 51%/49%. ^{13}C NMR (100 MHz, $CDCl_3$) δ 177.65, 177.62, 176.05, 169.98, 169.93, 66.58, 61.36, 61.31, 52.58, 44.60, 43.08, 43.00, 42.61, 42.54, 42.49, 40.98, 40.74, 39.48, 38.97, 31.02, 26.43, 26.36, 25.49, 25.35, 19.63, 14.78, 14.74, 14.31. ESI-MS (m/z): 431.10 ($M+Na^+$).

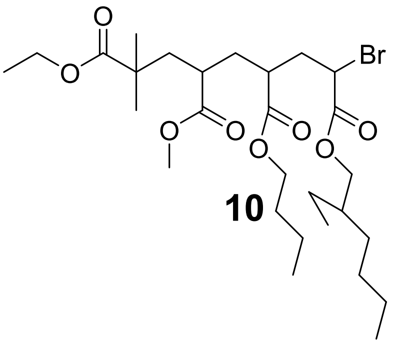

10

I-MA-BuA-EHA-Br (10). The same procedure as for synthesis of (**1**) was applied, using 1.220 mmol (0.225 g, 1 equiv) of the monomer EHA, 1.220 mmol (0.500 g, 1 equiv) of macroinitiator **9**, 0.017 mmol (3.800 mg, 0.014 equiv) of $CuBr_2$, 0.103 mmol (0.024 g, 0.084

equiv) of ME6TREN and 4 mL of DMSO (86 vol%). After reaction, the crude SUMI product was purified via recycling SEC to yield 0.100 g (14%) of pure **I-MA-BuA-EHA-Br (10)**. ^{1}H NMR (400 MHz, $CDCl_3$) δ 4.26 – 4.13 (m, 1H), 4.13 – 3.93 (m, 6H), 3.64 – 3.56 (m, 3H), 2.66 – 1.87 (m, 5H), 1.85 – 1.43 (m, 6H), 1.40 – 1.22 (m, 10H), 1.20 (t, J = 7.1 Hz, 3H), 1.12 (s, J = 2.7 Hz, 3H), 1.08 (s, J = 2.5 Hz, 3H), 0.94 – 0.79 (m, 9H). ^{13}C NMR (100 MHz, $CDCl_3$) δ 177.65, 176.59, 176.55, 176.52, 176.41, 174.63, 174.53, 170.11, 170.03, 169.96, 68.94, 65.50, 65.47, 65.43, 61.17, 52.39, 52.36, 52.28, 44.95, 44.63, 43.58, 43.45, 43.20, 43.06, 42.93, 42.74, 42.45, 42.17, 42.07, 41.75, 41.63, 40.74, 40.68, 40.45, 40.40, 39.31, 38.14, 37.67, 37.52, 37.26, 37.14, 36.66, 36.48, 31.20, 30.80, 29.46, 26.26, 26.19, 26.03, 25.73, 25.67, 25.50, 25.44, 24.25, 23.56, 19.73, 14.72, 14.68, 14.32, 11.56. ESI-MS (m/z): 615.25 ($M+Na^+$).

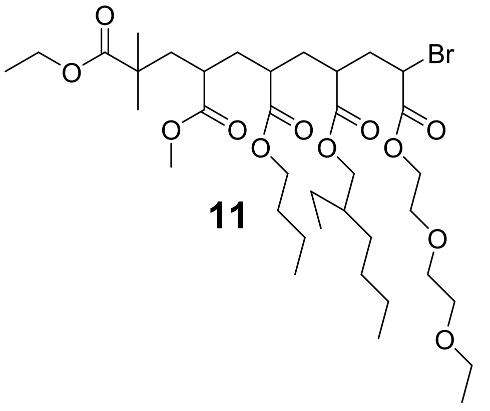

11

I-MA-BuA-EHA-DEGEEA-Br (11). The same procedure as for synthesis of (**1**) was applied, using 0.168 mmol (0.032 g, 1 equiv) of the monomer DEGEEA, 0.168 mmol (0.100 g, 1 equiv) of macroinitiator **10**, 0.0024 mmol (0.526 mg, 0.014 equiv) of $CuBr_2$, 0.014 mmol (3.260 mg, 0.084 equiv) of ME6TREN and 0.6 mL of DMSO (86 vol%). After reaction, the crude SUMI product was purified via recycling SEC to yield 0.010 g (8%) of pure **I-MA-BuA-EHA-DEGEEA-Br (11)**. ^{1}H NMR (300 MHz, $CDCl_3$) δ 4.39 – 4.17 (m, 3H), 4.16 – 3.88 (m, 6H), 3.70 (t, J = 4.9 Hz, 2H), 3.66 – 3.54 (m, 7H), 3.51 (q, J = 7.0 Hz, 2H), 2.76 –

1.85 (m, 6H), 1.85 – 1.44 (m, 8H), 1.44 – 1.15 (m, 16H), 1.13 (s, 3H), 1.10 (s, 3H), 0.97 – 0.81 (m, 9H). ^{13}C NMR (75 MHz, $CDCl_3$) δ 177.67, 176.55, 175.08, 174.76, 169.99, 169.76, 71.36, 70.42, 69.30, 67.92, 67.31, 65.74, 65.21, 61.12, 52.30, 52.20, 44.48, 43.70, 43.01, 42.82, 42.39, 41.94, 41.56, 40.71, 40.42, 39.22, 37.41, 37.01, 35.42, 31.21, 30.87, 29.47, 25.99, 25.63, 24.27, 23.55, 19.71, 15.79, 14.67, 14.32, 11.51. ESI-MS (m/z): 803.35 ($M+Na^+$).

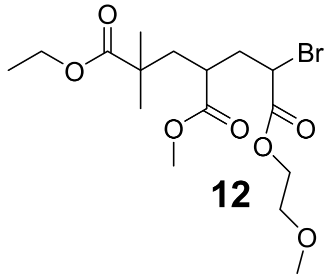

12

I-MA-EGMEA-Br (12). The same procedure as for synthesis of (**1**) was applied, using 7.110 mmol (0.926 g, 1 equiv) of the monomer EGMEA, 7.110 mmol (2.000 g, 1 equiv) of macroinitiator **1**, 0.100 mmol (0.022 g, 0.014 equiv) of $CuBr_2$, 0.600 mmol (0.138 g, 0.084 equiv) of ME6TREN and 2.6 mL of DMSO (50 vol%). After reaction, the crude SUMI product was purified with column chromatography (SiO_2, hexane/EtOAc 4/1) to yield 1.190 g (41%) of pure **I-MA-EGMEA-Br (12)**. ^1H NMR (300 MHz, $CDCl_3$) δ 4.31 – 4.22 (m, 2H), 4.22 – 4.12 (m, 1H), 4.12 – 4.00 (m, 2H), 3.62 (ds, J = 2.9 Hz, 3H), 3.60 – 3.53 (m, 2H), 3.34 (s, 3H), 2.76 – 2.41 (m, 1H), 2.36 – 1.95 (m, 3H), 1.62 (ddd, J = 14.3, 12.2, 3.3 Hz, 1H), 1.21 (t, J = 7.1 Hz, 3H), 1.14 (ds, J = 5.5 Hz, 3H), 1.09 (ds, J = 6.5 Hz, 3H), mixture of isomers 51%/49%. ^{13}C NMR (75 MHz, $cdcl_3$) δ 177.59, 175.97, 169.94, 169.86, 70.64, 65.62, 61.32, 61.27, 59.64, 52.55, 44.20, 43.06, 42.70, 42.58, 42.50, 42.39, 40.85, 40.66, 39.33, 38.86, 26.36, 26.30, 25.48, 25.33, 14.75. ESI-MS (m/z): 433.08 ($M+Na^+$).

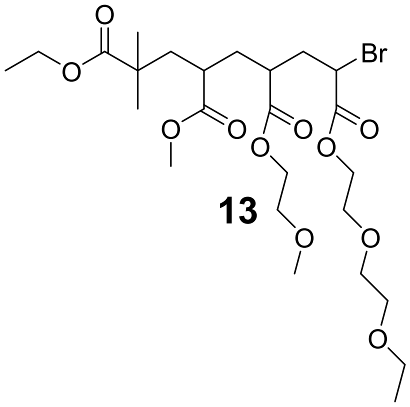

13

I-MA-EGMEA-DEGEEA-Br (13). The same procedure as for synthesis of (**1**) was applied, using 2.890 mmol (0.545 g, 1 equiv) of the monomer DEGEEA, 2.890 mmol (1.190 g, 1 equiv) of macroinitiator **12**, 0.040 mmol (0.009 g, 0.014 equiv) of $CuBr_2$, 0.243 mmol (0.056 g, 0.084 equiv) of ME6TREN and 3.5 mL of DMSO (70 vol%). After reaction, the crude SUMI product was purified with column chromatography (SiO_2, hexane/EtOAc 4/1) to yield 0.380 g (22%) of pure **I-MA-EGMEA-DEGEEA-Br (13)**. ^{1}H NMR (300 MHz, $CDCl_3$) δ 4.35 – 4.10 (m, 5H), 4.09 – 3.96 (m, 2H), 3.67 (t, J = 6.2, 3.3 Hz, 2H), 3.63 – 3.50 (m, 9H), 3.47 (q, J = 7.1 Hz, 2H), 3.31 (s, 3H), 2.70 – 2.33 (m, 2H), 2.30 – 1.92 (m, 4H), 1.79 – 1.43 (m, 2H), 1.24 – 1.12 (m, 6H), 1.11 (s, 3H), 1.07 (s, 3H). ^{13}C NMR (75 MHz, $CDCl_3$) δ 177.62, 176.48, 176.35, 174.39, 174.32, 170.00, 169.95, 169.80, 71.28, 71.03, 70.79, 70.36, 69.25, 67.23, 65.65, 64.18, 61.10, 59.40, 52.30, 52.24, 44.43, 44.17, 43.44, 43.33, 42.94, 42.62, 42.39, 41.99, 41.88, 41.57, 41.51, 40.53, 40.30, 37.95, 37.57, 37.36, 37.09, 36.97, 36.47, 36.33, 26.09, 25.93, 25.61, 25.39, 15.72, 14.66. ESI-MS (m/z): 621.19 ($M+Na^+$).

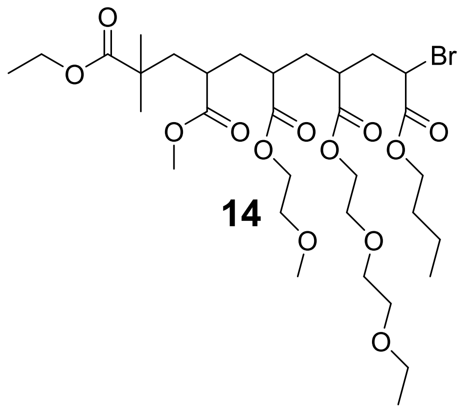

14

I-MA-EGMEA-DEGEEA-BuA-Br (14). The same procedure as for synthesis of (**1**) was applied, using 0.617 mmol (0.079 g, 1 equiv) of the monomer BuA, 0.617 mmol (0.370 g, 1 equiv) of macroinitiator **13**, 0.0086 mmol (1.900 mg, 0.014 equiv) of $CuBr_2$, 0.052 mmol (0.012 g, 0.084 equiv) of ME6TREN and 0.45 mL of DMSO (50 vol%). After reaction, the crude SUMI product was purified via recycling SEC to yield 0.060 g (13%) of pure **I-MA-EGMEA-DEGEEA-BuA-Br (14)**. ^{1}H NMR (300 MHz, $CDCl_3$) δ 4.35 – 3.96 (m, 9H), 3.71 – 3.41 (m, 13H), 3.31 (s, 3H), 2.76 – 1.83 (m, 7H), 1.83 – 1.45 (m, 6H), 1.45 – 1.26 (m, 2H), 1.22 – 1.12 (m, 6H), 1.10 (s, 3H), 1.07 (s, 3H), 0.89 (t, J = 7.0 Hz, 3H). ^{13}C NMR (75 MHz, $CDCl_3$) δ 177.71, 176.62, 174.99, 174.92, 174.53, 174.43, 174.37, 170.04, 169.91, 71.22, 70.90, 70.43, 69.52, 67.29, 66.52, 64.36, 64.25, 64.14, 61.14, 59.45, 52.36, 52.23, 44.91, 44.75, 43.66, 43.54, 42.93, 42.78, 42.43, 42.08, 41.96, 41.76, 41.58, 41.47, 40.63, 40.40, 37.60, 37.39, 37.20, 36.93, 36.82, 35.67, 35.18, 31.00, 26.03, 25.55, 19.60, 15.80, 14.73, 14.29.ESI-MS (m/z): 749.27 ($M+Na^{+}$).

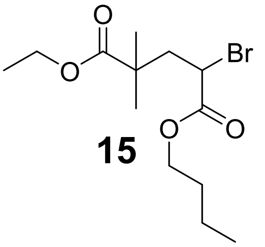

15

I-BuA-Br (15). The same procedure as for synthesis of (**1**) was applied, using 11.615 mmol (1.490 g, 1 equiv) of the monomer BuA, 11.615 mmol (2.270 g, 1 equiv) of EBiB initiator, 0.163 mmol (0.036 g, 0.014 equiv) of $CuBr_2$, 0.975 mmol (0.225 g, 0.084 equiv) of ME6TREN and 3.6 mL of DMSO (50 vol%). After reaction, the crude SUMI product was purified via recycling SEC to yield 1.5 g (40%) of pure **I-BuA-Br (15)**. ^{1}H NMR (400 MHz, $CDCl_3$) δ 4.23 (dd, *J* = 8.7, 4.9 Hz, 1H), 4.15 – 4.02 (m, 4H), 2.48 (dd, *J* = 14.7, 8.7 Hz, 1H), 2.28 (dd, *J* = 14.7, 4.9 Hz, 1H), 1.65 – 1.54 (m, 2H), 1.42 – 1.29 (m, 2H), 1.21 (t, *J* = 7.1 Hz, 3H), 1.18 (s, 3H), 1.11 (s, 3H), 0.89 (t, *J* = 7.4 Hz, 3H). ^{13}C NMR (100 MHz, $CDCl_3$) δ 176.98, 170.80, 66.43, 61.43, 45.25, 42.89, 42.18, 30.91, 26.57, 24.88, 19.61, 14.67, 14.27. ESI-MS (m/z): 345.07 ($M+Na^+$).

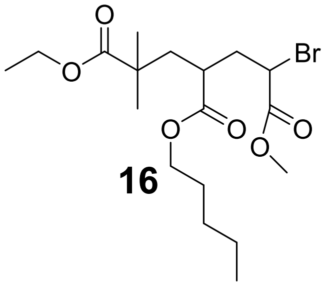

16

I-BuA-MA-Br (16). The same procedure as for synthesis of (**1**) was applied, using 4.640 mmol (0.400 g, 1 equiv) of the monomer MA, 4.640 mmol (1.500 g, 1 equiv) of macroinitiator **15**, 0.065 mmol (0.015 g, 0.014 equiv) of $CuBr_2$, 0.389 mmol (0.090 g, 0.084 equiv) of ME6TREN and 3 mL of DMSO (60 vol%). After reaction, the crude SUMI product was purified with column chromatography (SiO_2, hexane/EtOAc 4/1) to yield 0.810 g (43%) of pure **I-BuA-MA-Br (16)**. ^{1}H NMR (400 MHz, $CDCl_3$) δ 4.18 – 3.94 (m, 5H), 3.73 (ds, *J* =

4.9 Hz, 3H), 2.69 – 2.37 (m, 1H), 2.34 – 1.95 (m, 3H), 1.76 – 1.51 (m, 3H), 1.41 – 1.27 (m, 2H), 1.22 (dt, J = 7.1, 1.2 Hz, 3H), 1.14 (ds, J = 8.1 Hz, 3H), 1.10 (ds, J = 11.6 Hz, 3H), 0.89 (dt, J = 7.3, 0.8 Hz, 3H), mixture of isomers 51%/49%. ^{13}C NMR (100 MHz, $CDCl_3$) δ 177.64, 177.61, 175.64, 170.37, 170.31, 65.44, 65.40, 61.32, 61.27, 53.64, 53.62, 44.17, 42.91, 42.66, 42.61, 42.41, 42.38, 41.20, 40.86, 39.61, 39.01, 31.11, 31.07, 26.71, 26.65, 25.12, 24.94, 19.75, 19.72, 14.75, 14.72, 14.31. ESI-MS (m/z): 431.10 ($M+Na^+$).

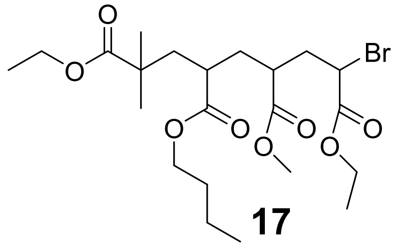

17

I-BuA-MA-EA-Br (17). The same procedure as for synthesis of (**1**) was applied, using 1.980 mmol (0.198 g, 1 equiv) of the monomer EA, 1.980 mmol (0.81 g, 1 equiv) of macroinitiator **16**, 0.028 mmol (0.006 g, 0.014 equiv) of $CuBr_2$, 0.166 mmol (0.038 g, 0.084 equiv) of ME6TREN and 4 mL of DMSO (80 vol%). After reaction, the crude SUMI product was purified with column chromatography (SiO_2, hexane/EtOAc 4/1) to yield 0.240 g (24%) of pure **I-BuA-MA-EA-Br (17)**. ^{1}H NMR (400 MHz, $CDCl_3$) δ 4.23 – 4.09 (m, 3H), 4.09 – 3.90 (m, 4H), 3.68 – 3.59 (m, 3H), 2.69 – 1.82 (m, 5H), 1.79 – 1.41 (m, 5H), 1.39 – 1.26 (m, 2H), 1.26 – 1.15 (m, 6H), 1.10 (s, 3H), 1.06 (s, 3H), 0.87 (td, J = 7.4, 2.4 Hz, 3H). ^{13}C NMR (100 MHz, $CDCl_3$) δ 177.64, 176.18, 176.13, 175.06, 174.99, 174.96, 174.89, 169.87, 169.80, 169.66, 65.23, 65.13, 62.79, 62.70, 61.11, 52.57, 52.55, 52.49, 44.76, 44.40, 43.42, 43.29, 43.08, 42.95, 42.58, 42.52, 41.88, 41.82, 41.63, 41.48, 40.88, 40.76, 40.72, 40.63, 38.12, 37.72, 37.37, 37.31, 37.17, 36.67, 36.52, 36.45, 31.07, 26.55, 26.39, 25.21, 25.15, 25.03, 19.71, 14.68, 14.55, 14.47, 14.30. ESI-MS (m/z): 531.16 ($M+Na^+$).

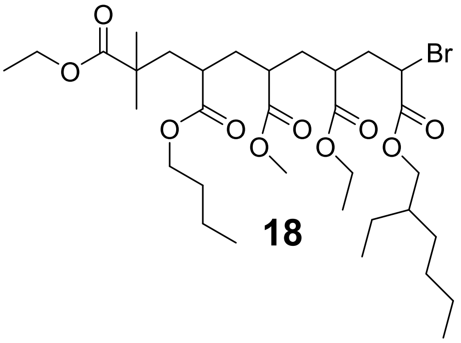

18

I-BuA-MA-EA-EHA-Br (18). The same procedure as for synthesis of (**1**) was applied, using 0.471 mmol (0.087 g, 1 equiv) of the monomer EHA, 0.471 mmol (0.240 g, 1 equiv) of macroinitiator **17**, 0.0066 mmol (1.470 mg, 0.014 equiv) of $CuBr_2$, 0.040 mmol (0.009 g, 0.084 equiv) of ME6TREN and 0.6 mL of DMSO (69 vol%). After reaction, the crude SUMI product was purified via recycling SEC to yield 0.040 g (12%) of pure **I-BuA-MA-EA-EHA-Br (18)**. ^{1}H NMR (300 MHz, $CDCl_3$) δ 4.29 – 3.89 (m, 9H), 3.71 – 3.58 (m, 3H), 2.70 – 1.83 (m, 7H), 1.83 – 1.44 (m, 7H), 1.44 – 1.16 (m, 16H), 1.12 (s, 3H), 1.08 (s, 3H), 0.98 – 0.76 (m, 9H). ^{13}C NMR (75 MHz, $CDCl_3$) δ 177.77, 176.33, 176.24, 175.54, 174.55, 174.48, 170.07, 169.98, 69.12, 68.98, 65.27, 65.11, 61.62, 61.53, 61.16, 52.34, 44.95, 44.69, 43.58, 43.49, 43.17, 42.75, 42.57, 42.07, 41.75, 41.62, 41.49, 40.92, 40.85, 39.32, 38.02, 37.78, 37.64, 37.40, 37.14, 36.09, 35.37, 31.14, 30.82, 29.48, 26.48, 25.17, 24.26, 23.57, 19.78, 14.76, 14.69, 14.37, 11.57. ESI-MS (m/z): 715.30 ($M+Na^{+}$).

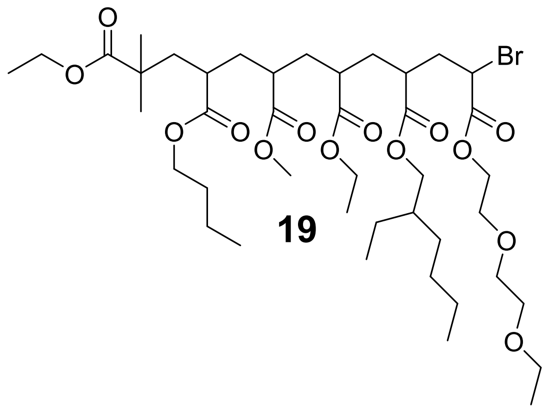

19

I-BuA-MA-EA-EHA-DEGEEA-Br (19). The same procedure as for synthesis of (**1**) was applied, using 0.058 mmol (0.011 g, 1 equiv) of the monomer DEGEEA, 0.058 mmol (0.040 g, 1 equiv) of macroinitiator **18**, 0.0008 mmol (0.180 mg, 0.014 equiv) of $CuBr_2$, 0.0048 mmol (1.100 mg, 0.084 equiv) of ME6TREN and 0.2 mL of DMSO (80 vol%). After reaction, the crude SUMI product was purified via recycling SEC to yield 0.001 g (2%) of pure **I-BuA-MA-EA-EHA-DEGEEA-Br (19)**. The amount of sample was in this case so small that only ^{1}H NMR and ESI-MS analysis could be carried out. ^{1}H NMR (300 MHz, $CDCl_3$) δ 4.40 – 4.19 (m, 3H), 4.19 – 3.88 (m, 8H), 3.70 (t, J = 4.8 Hz, 2H), 3.67 – 3.54 (m, 7H), 3.51 (q, J = 7.1 Hz, 2H), 2.77 – 1.83 (m, 8H), 1.83 – 1.46 (m, 9H), 1.44 – 1.16 (m, 19H), 1.13 (s, 3H), 1.09 (s, 3H), 0.99 – 0.80 (m, 9H). ESI-MS (m/z): 903.41 (M+Na^+).

Table S1. Individual yields for all isolated SUMI products and overall yields for each SUMI reaction*.

Reaction	SUMI product	Isolated Yield /%	Overall Isolated Yield /%
1	I-Br	n. i.	67
	I-MA-Br (**1**)	43	
	I-(MA)$_2$-Br	24	
2	I-MA-Br	9	64
	I-MA-EA-Br (**2**)	47	
	I-MA-(EA)$_2$-Br	8	
3	I-MA-EA-Br	26	64
	I-MA-EA-EGMEA-Br (**3**)	21	
	I-MA-EA-(EGMEA)$_2$-Br	17	
4	I-MA-EA-EGMEA-Br	19	37
	I-MA-EA-EGMEA-DEGEEA-Br (**4**)	18	
	I-MA-EA-EGMEA-(DEGEEA)$_2$-Br	n. i.	
5	I-MA-EA-EGMEA-DEGEEA-Br	5	26
	I-MA-EA-EGMEA-DEGEEA-EHA-Br (**5**)	10	
	I-MA-EA-EGMEA-DEGEEA-(EHA)$_2$-Br	11	
9	I-MA-Br	5	68
	I-MA-BuA-Br (**9**)	43	
	I-MA-(BuA)$_2$-Br	16	
	I-MA-(BuA)$_3$-Br	4	
10	I-MA-BuA-Br	7	34
	I-MA-BuA-EHA-Br (**10**)	14	
	I-MA-BuA-(EHA)$_2$-Br	9	
	I-MA-BuA-(EHA)$_3$-Br	4	
11	I-MA-BuA-EHA-Br	15	30
	I-MA-BuA-EHA-DEGEEA-Br (**11**)	8	
	I-MA-BuA-EHA-(DEGEEA)$_2$-Br	7	
12	I-MA-Br	11	52
	I-MA-EGMEA-Br (**12**)	41	
	I-MA-(EGMEA)$_2$-Br	n.i.	
13	I-MA-EGMEA-Br	41	63
	I-MA-EGMEA-DEGEEA-Br (**13**)	22	
	I-MA-EGMEA-(DEGEEA)$_2$-Br	n. i.	
14	I-MA-EGMEA-DEGEEA-Br	7	31
	I-MA-EGMEA-DEGEEA-BuA-Br (**14**)	13	
	I-MA-EGMEA-DEGEEA-(BuA)$_2$-Br	11	
15	I-Br	n. i.	62
	I-BuA-Br (**15**)	40	
	I-(BuA)$_2$-Br	22	
16	I-BuA-Br	16	78
	I-BuA-MA-Br (**16**)	43	
	I-BuA-(MA)$_2$-Br	16	
	I-BuA-(MA)$_3$-Br	3	

* Only the optimized SUMI reactions are displayed, where no or only very limited amount of Cl-end capped byproducts were formed.

4. Spectroscopic Data

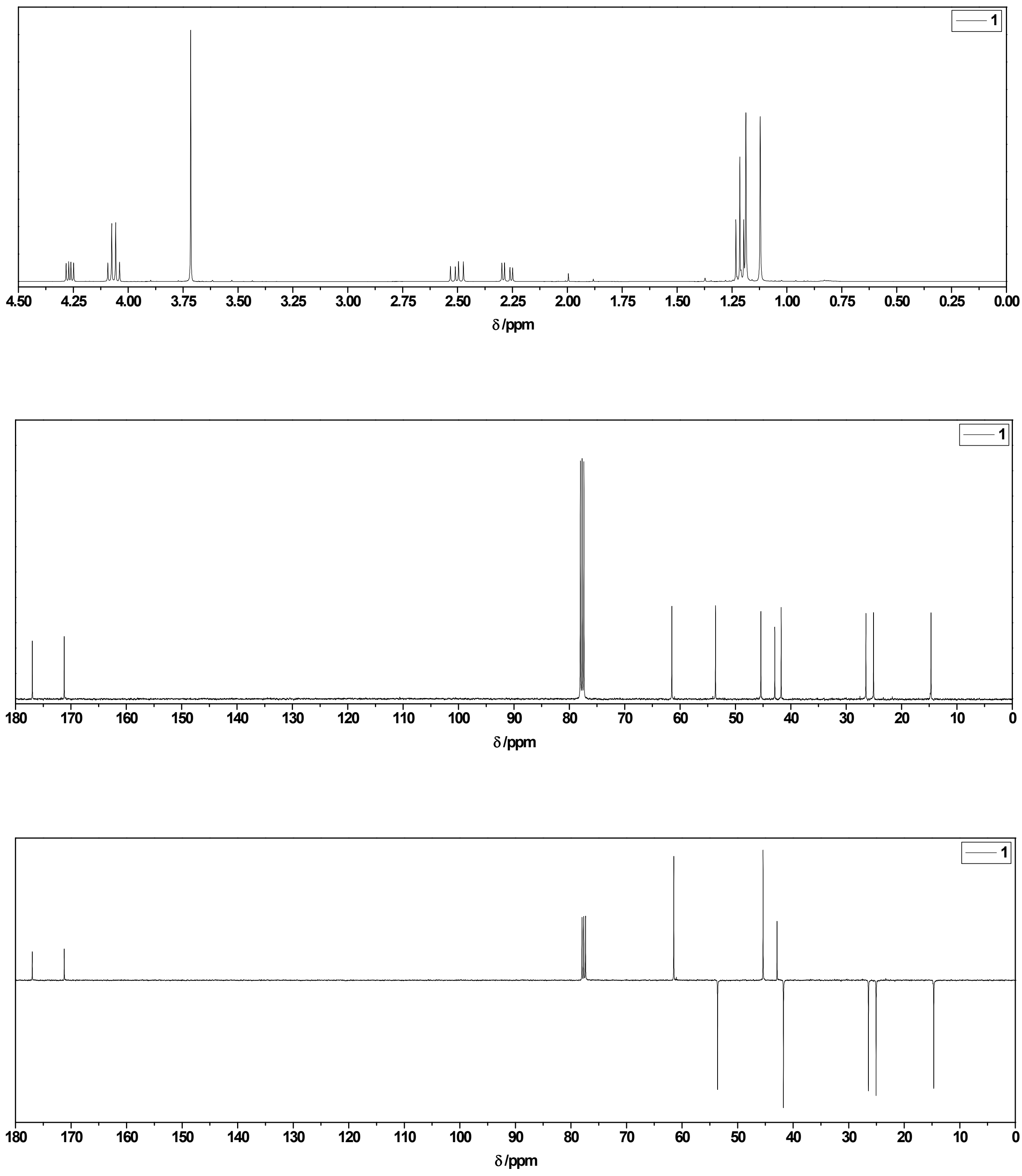

Figure S2. ^{1}H NMR, ^{13}C NMR and APT ^{13}C NMR spectrum of **I-MA-Br 1**.

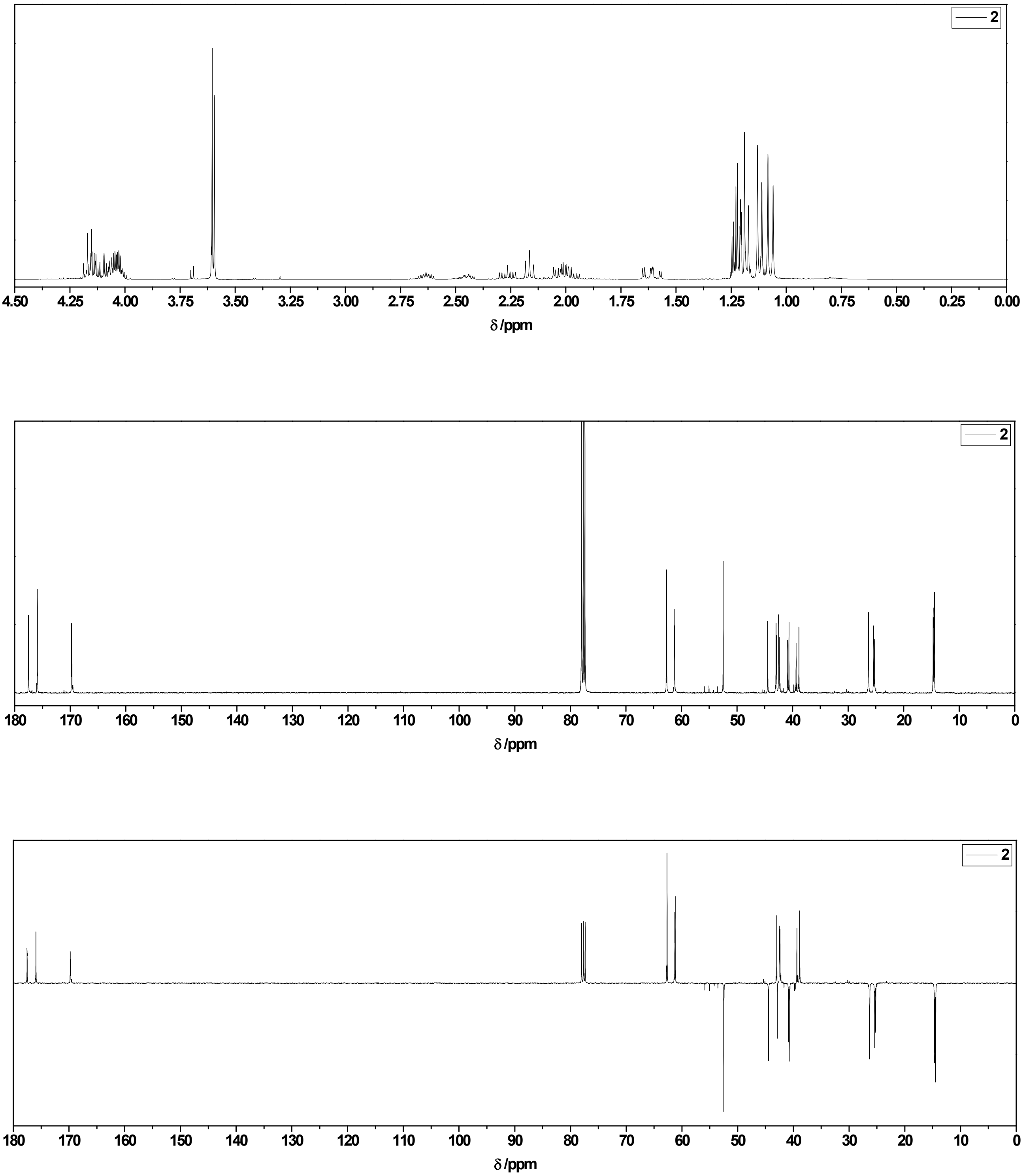

Figure S3. ^{1}H NMR, ^{13}C NMR and APT ^{13}C NMR spectrum of **I-MA-EA-Br 2**.

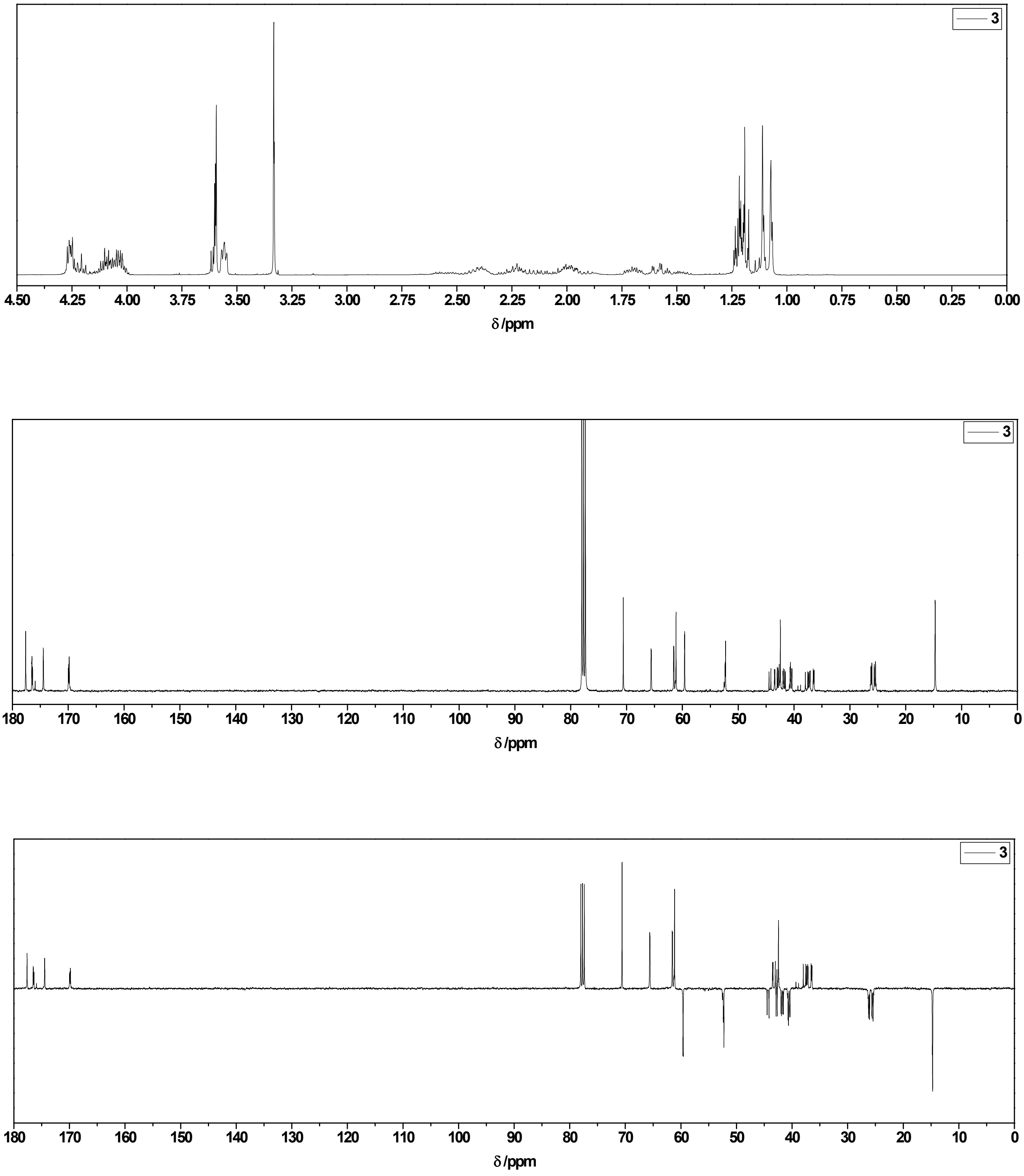

Figure S4. ^{1}H NMR, ^{13}C NMR and APT ^{13}C NMR spectrum of **I-MA-EA-EGMEA-Br 3**.

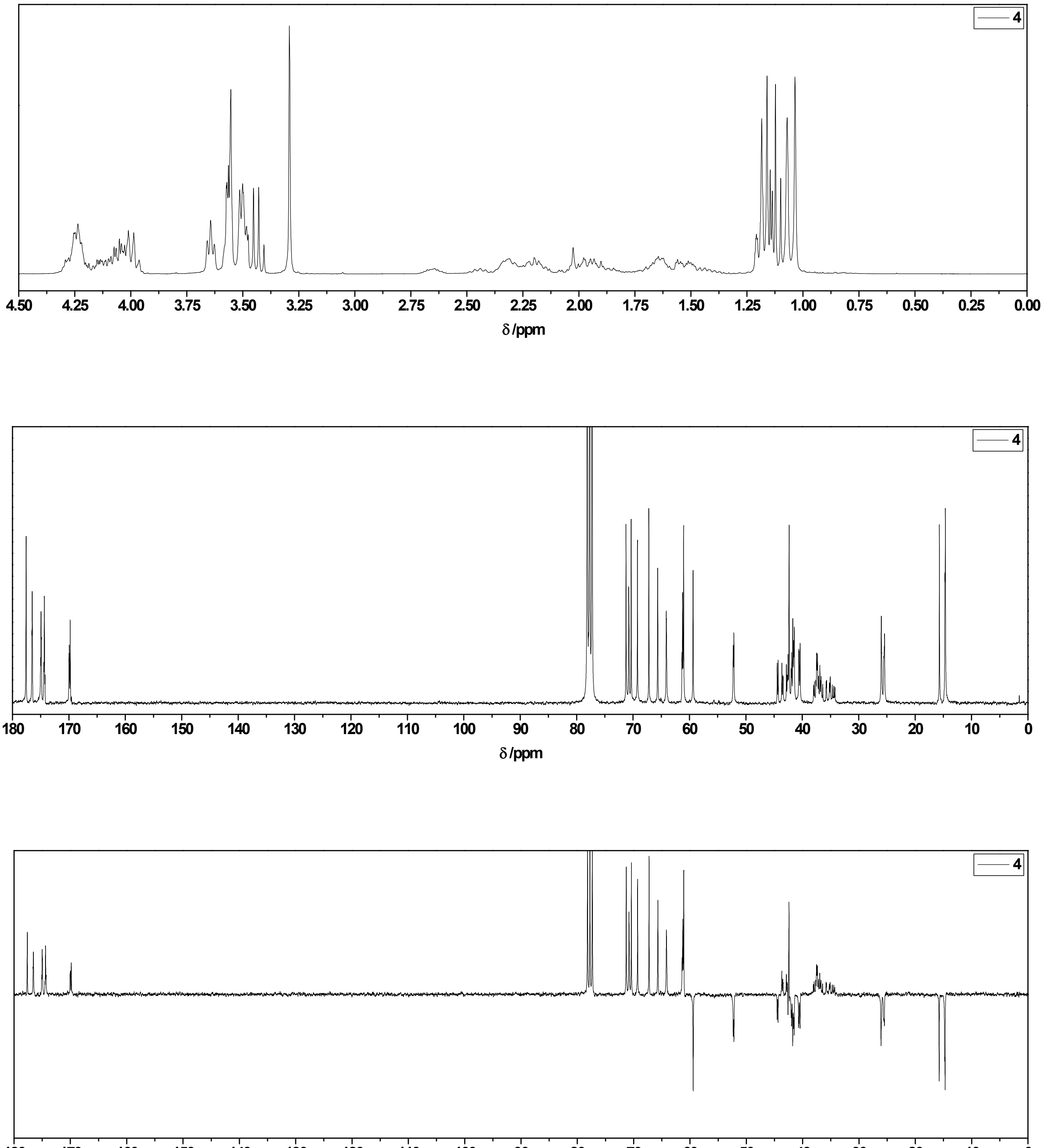

Figure S5. ^{1}H NMR, ^{13}C NMR and APT ^{13}C NMR spectrum of **I-MA-EA-EGMEA-DEGEEA-Br 4**.

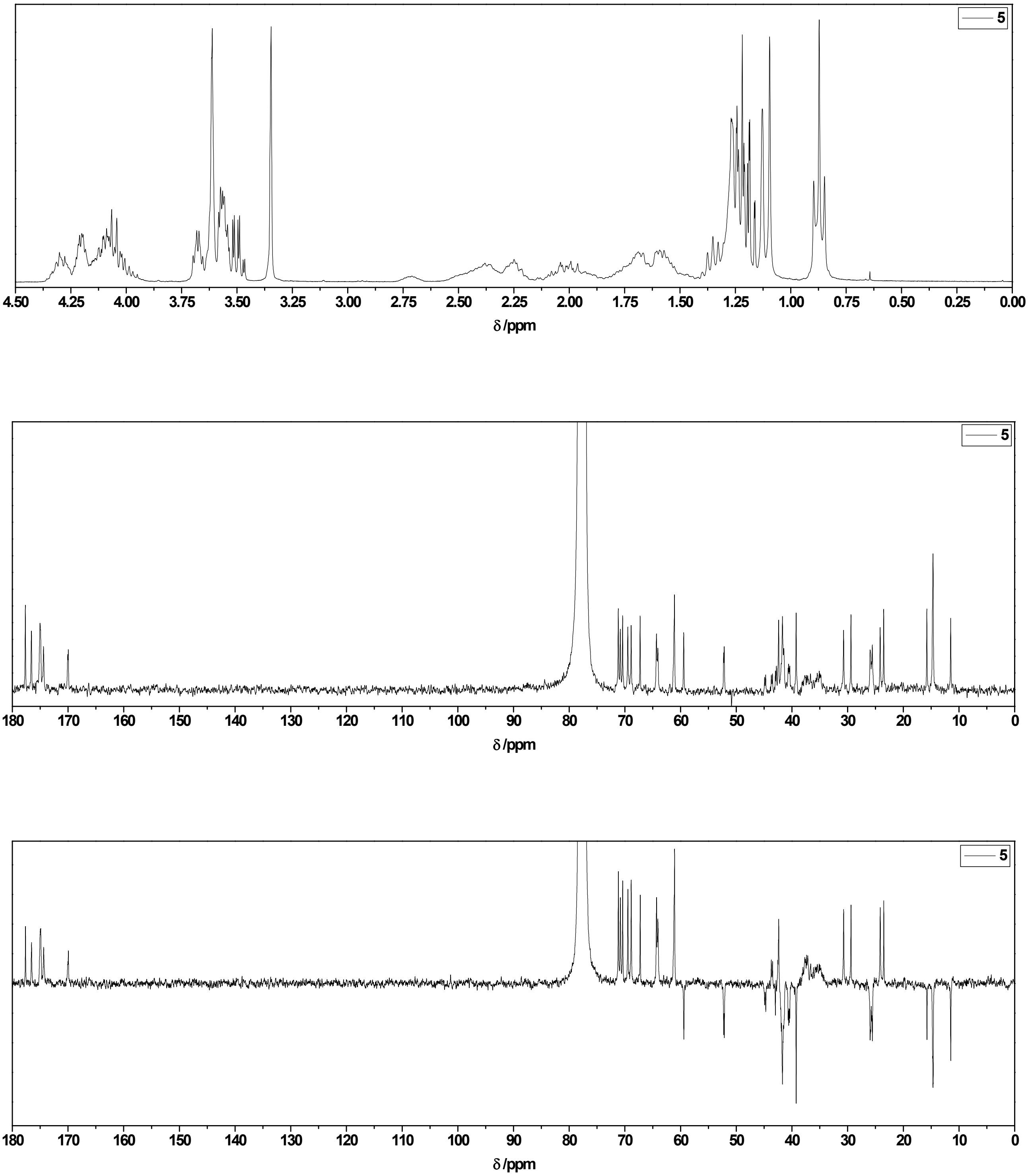

Figure S6. ^{1}H NMR, ^{13}C NMR and APT ^{13}C NMR spectrum of **I-MA-EA-EGMEA-DEGEEA-EHA-Br 5**.

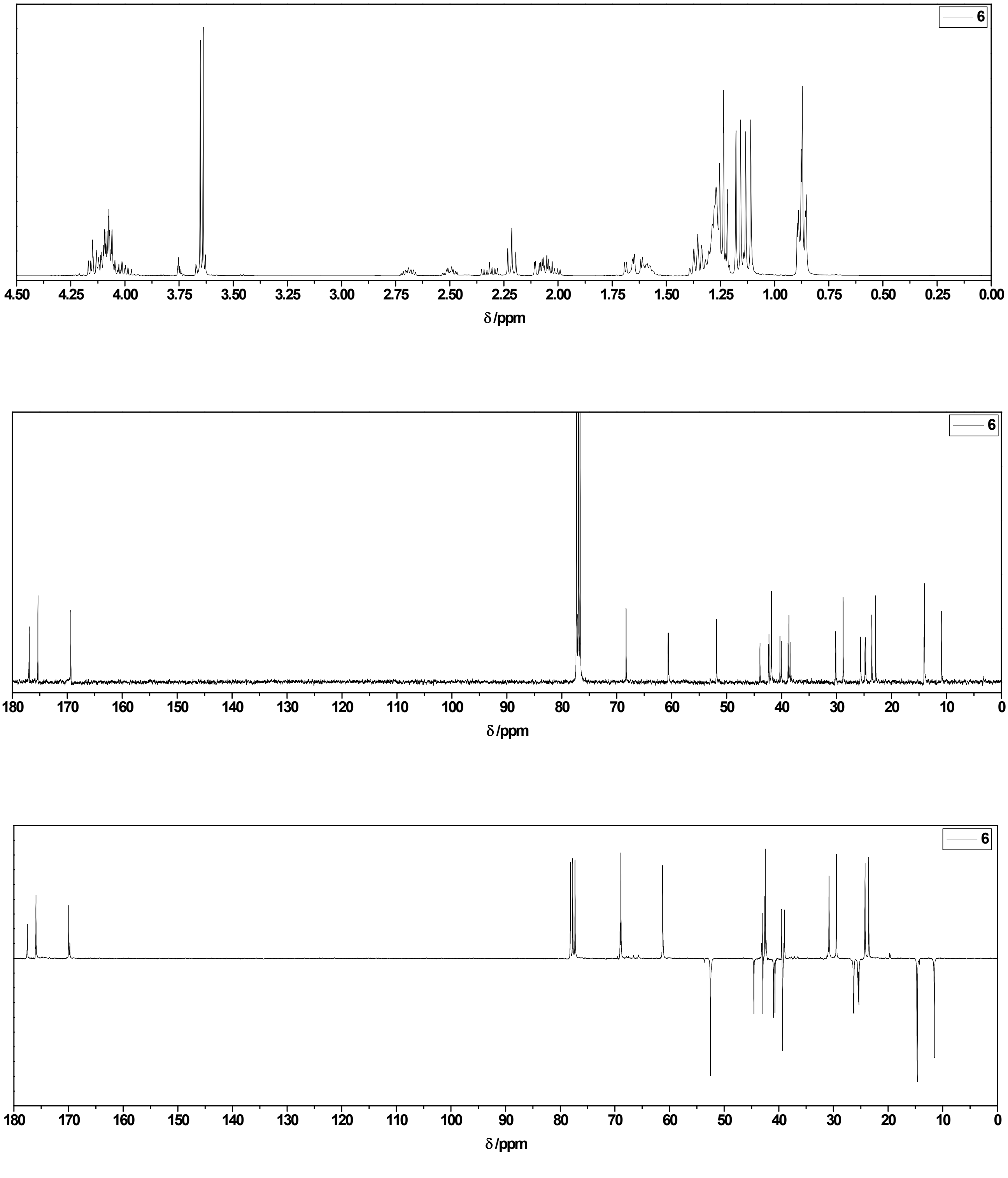

Figure S7. ^{1}H NMR, ^{13}C NMR and APT ^{13}C NMR spectrum of **I-MA-EHA-Br 6**.

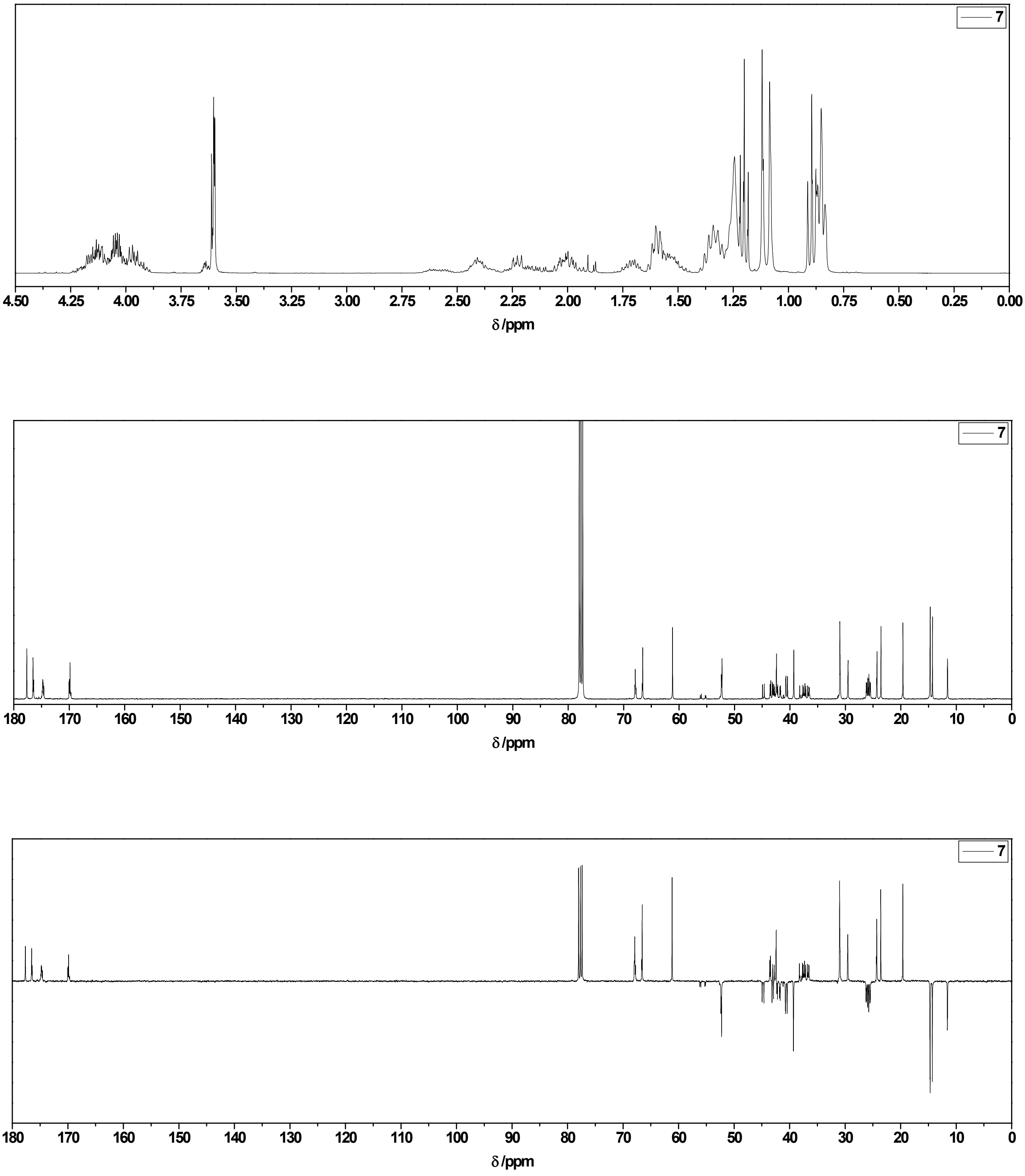

Figure S8. ^{1}H NMR, ^{13}C NMR and APT ^{13}C NMR spectrum of **I-MA-EHA-BuA-Br 7**.

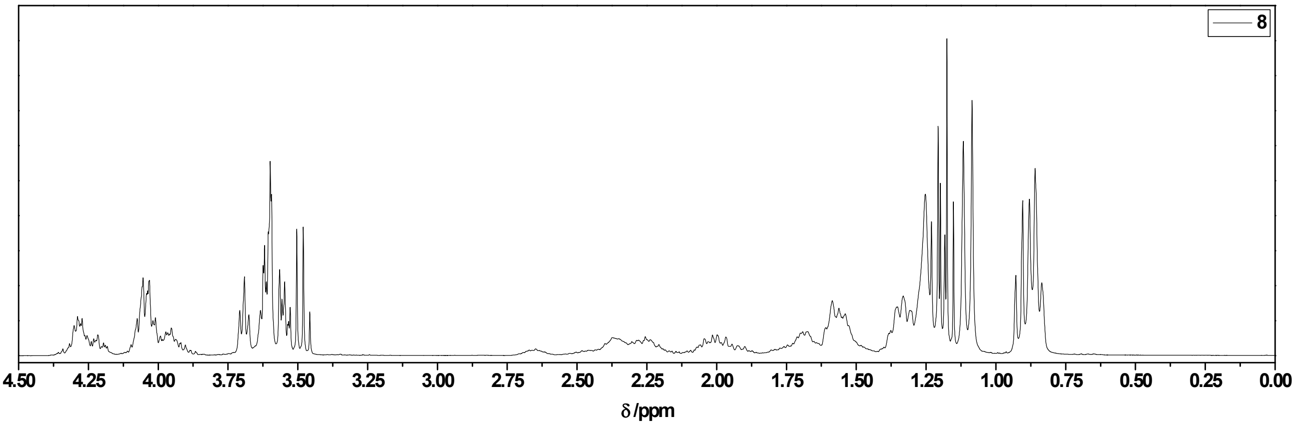

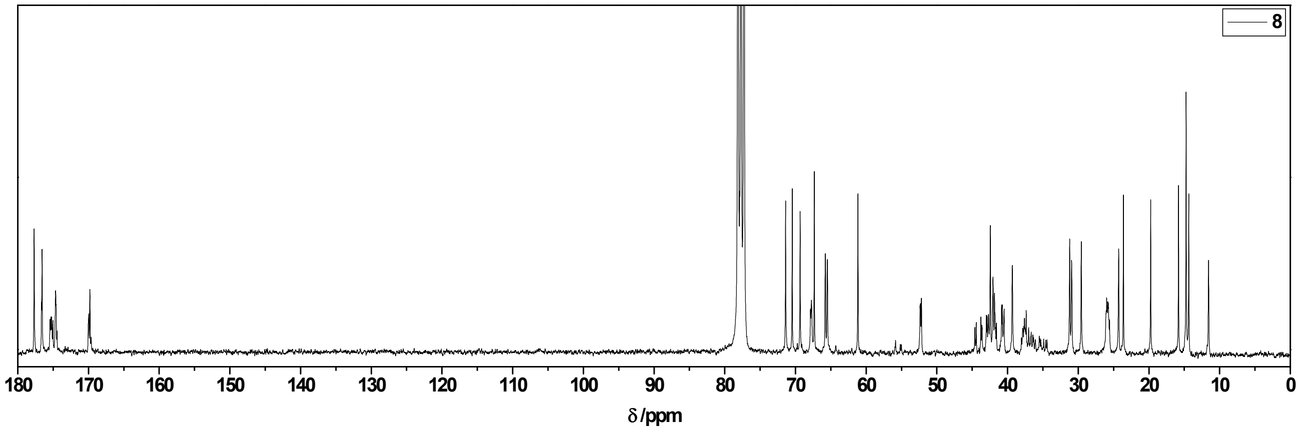

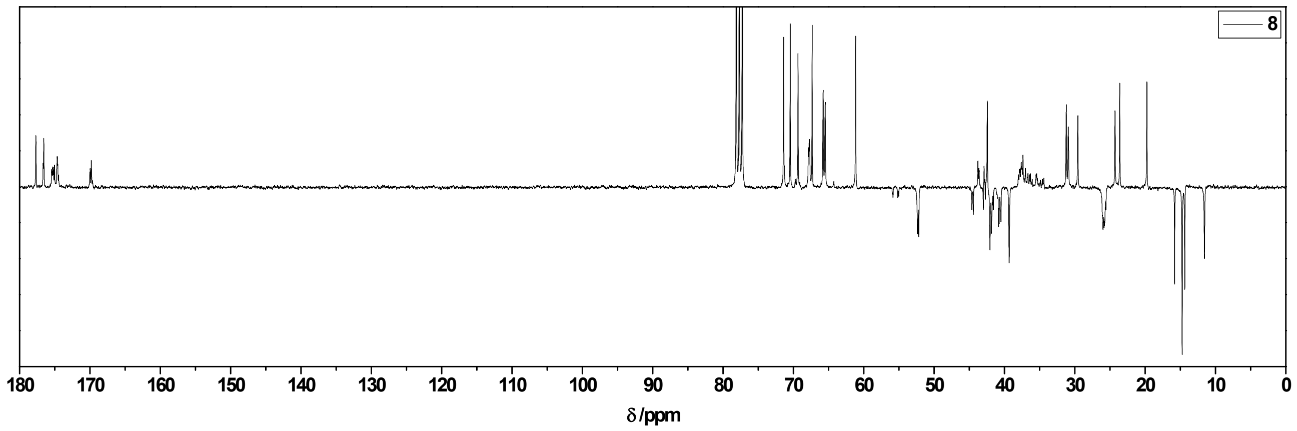

Figure S9. ^{1}H NMR, ^{13}C NMR and APT ^{13}C NMR spectrum of **I-MA-EHA-BuA-DEGEEA-Br 8**.

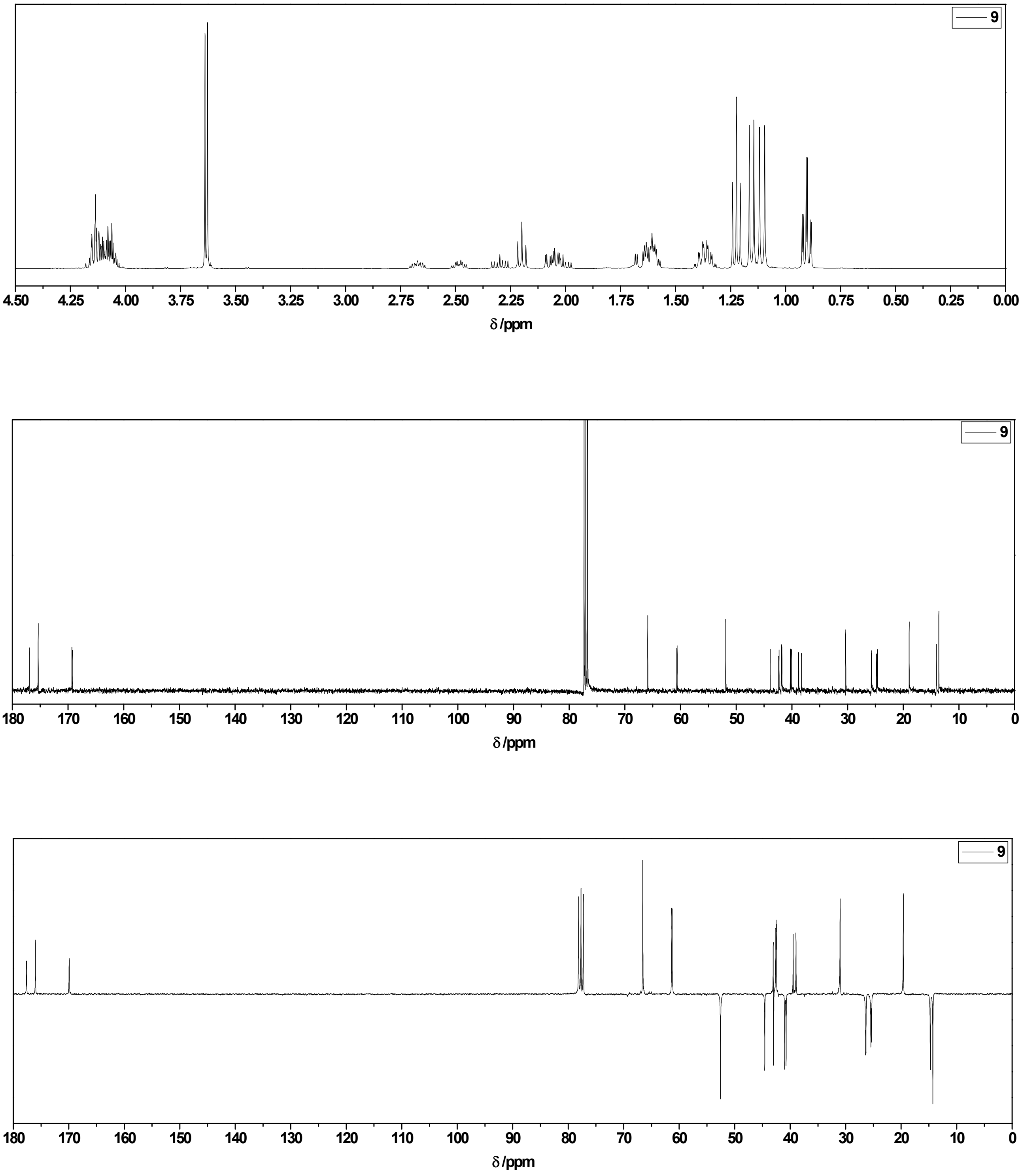

Figure S10. ^{1}H NMR, ^{13}C NMR and APT ^{13}C NMR spectrum of **I-MA-BuA-Br 9**.

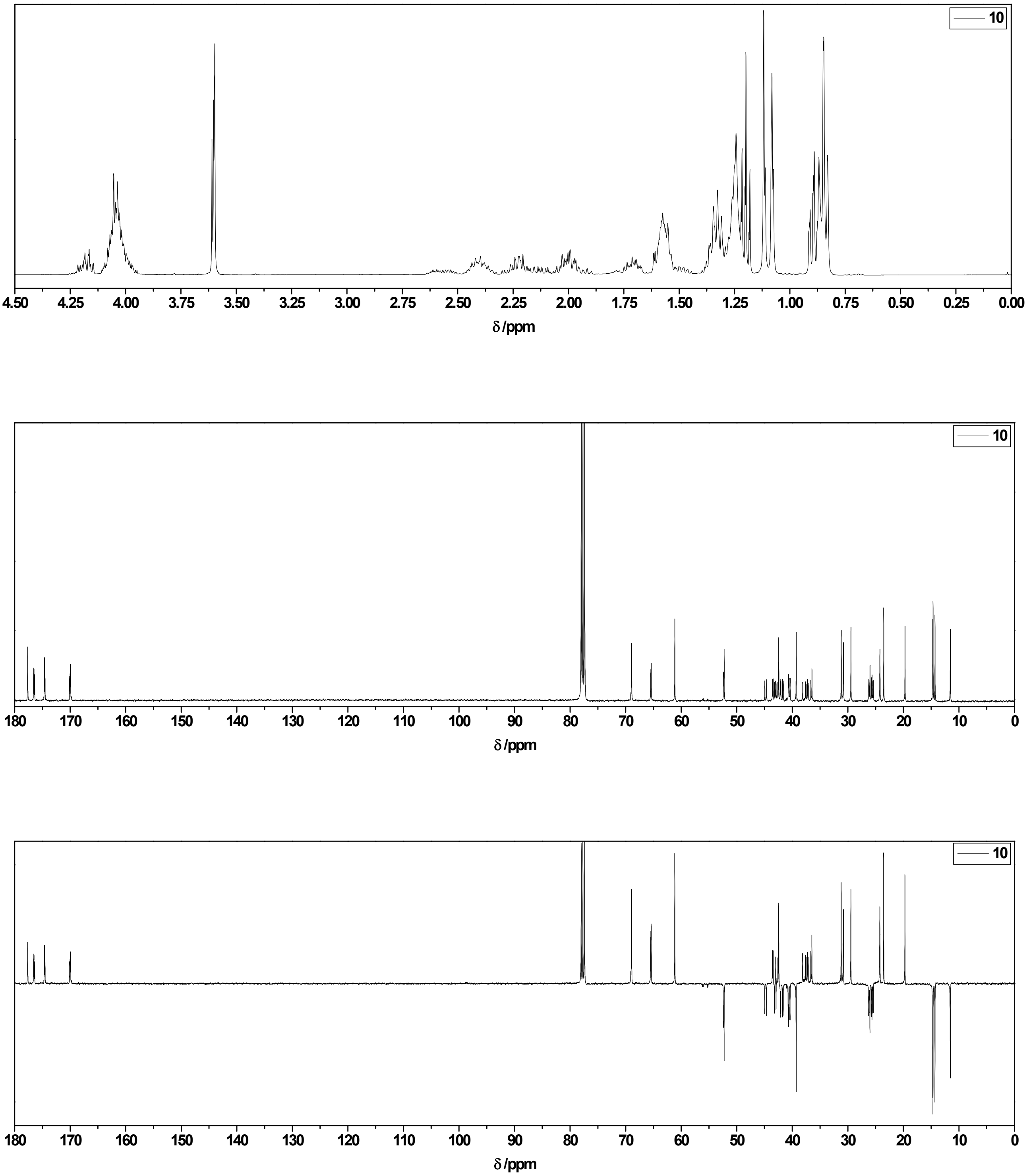

Figure S11. ^{1}H NMR, ^{13}C NMR and APT ^{13}C NMR spectrum of **I-MA-BuA-EHA-Br 10**.

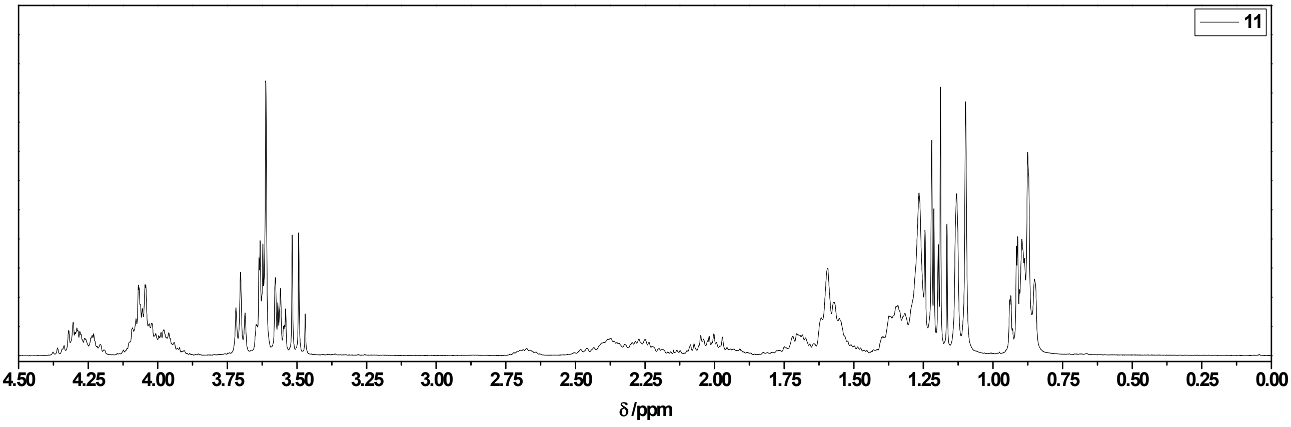

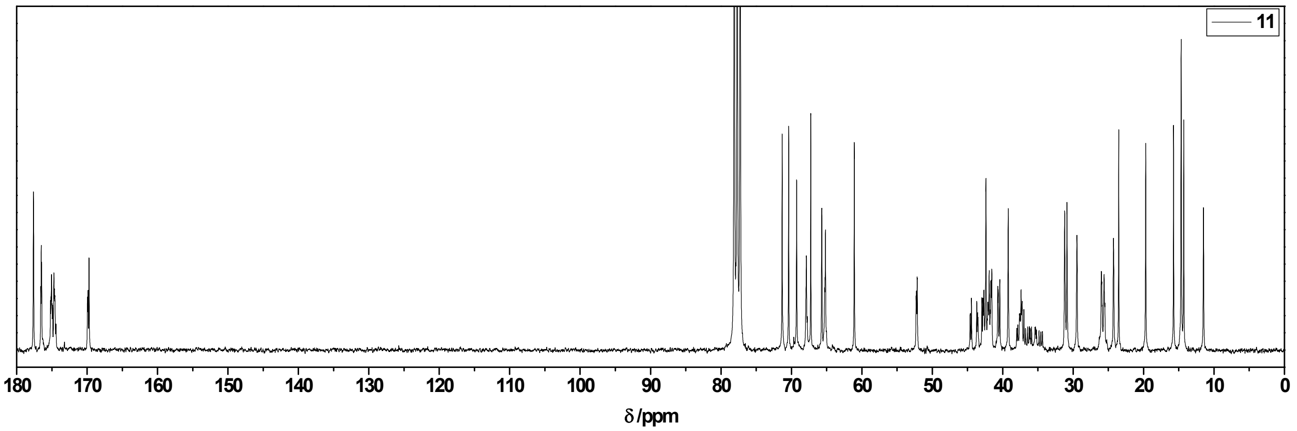

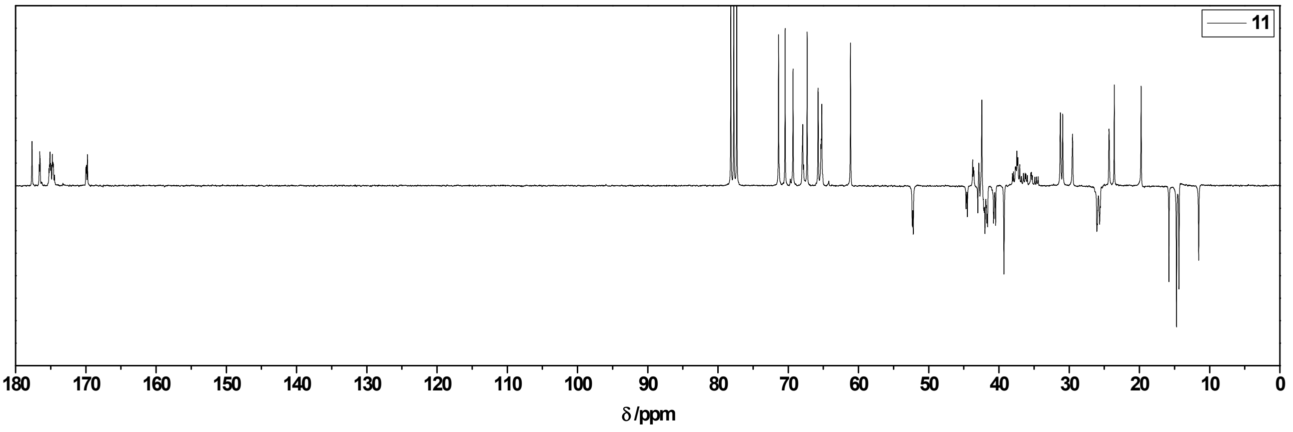

Figure S12. ^{1}H NMR, ^{13}C NMR and APT ^{13}C NMR spectrum of **I-MA-BuA-EHA-DEGEEA-Br 11**.

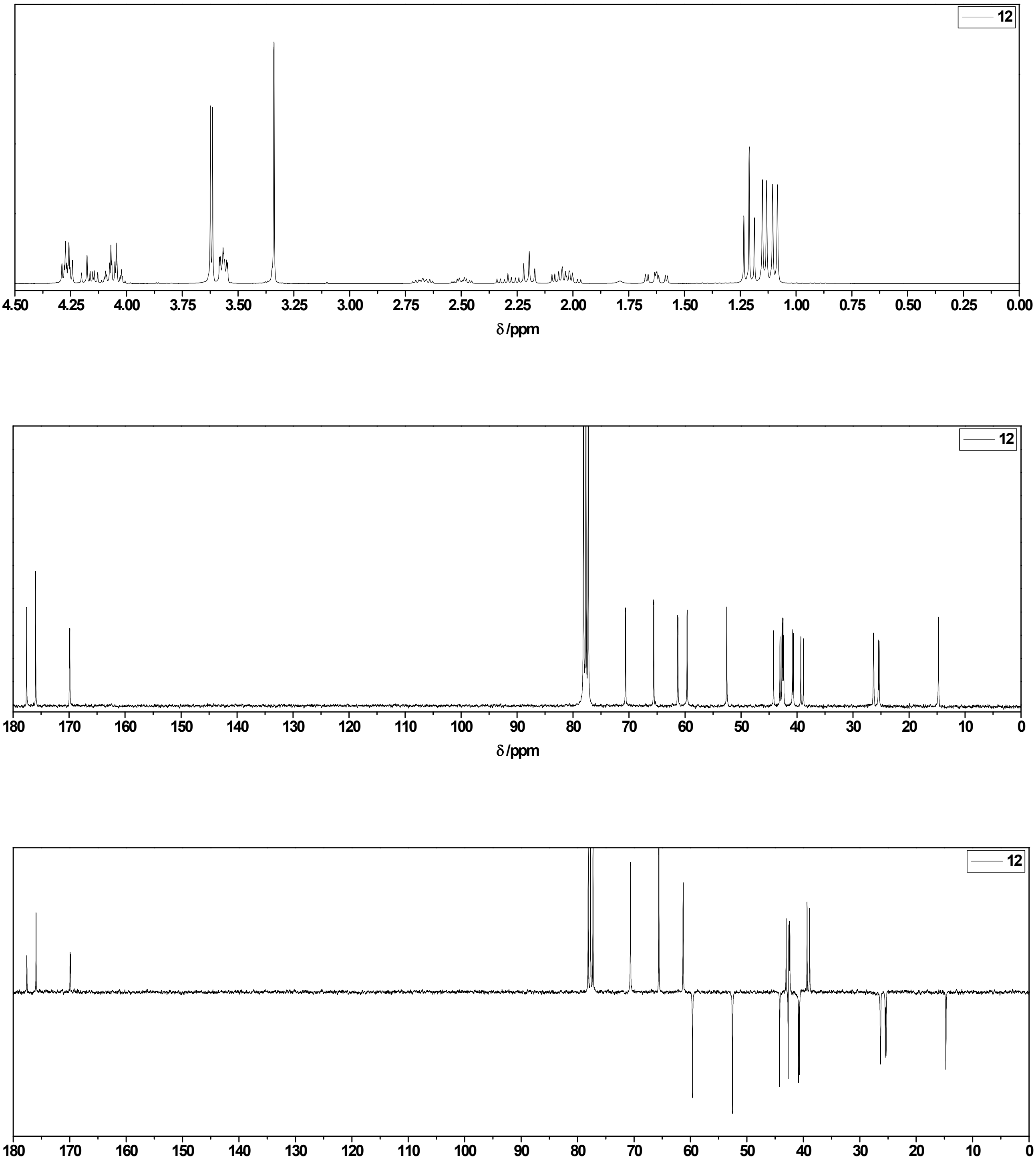

Figure S13. ^{1}H NMR, ^{13}C NMR and APT ^{13}C NMR spectrum of **I-MA-EGMEA-Br 12**.

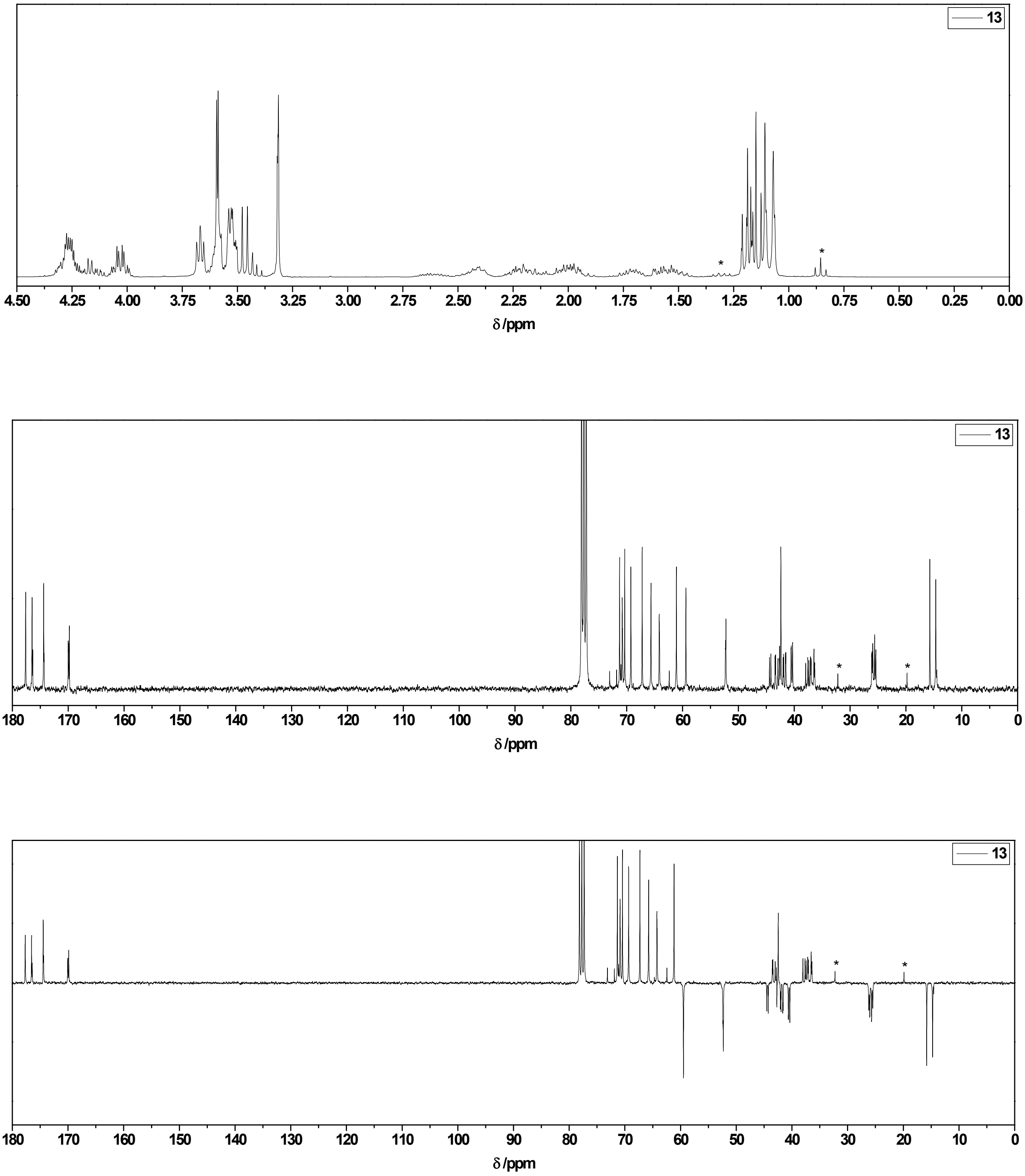

Figure S14. ^{1}H NMR, ^{13}C NMR and APT ^{13}C NMR spectrum of **I-MA-EGMEA-DEGEEA-Br 13**. The resonances marked with an asterisk result from residual leftovers of hexane solvent.

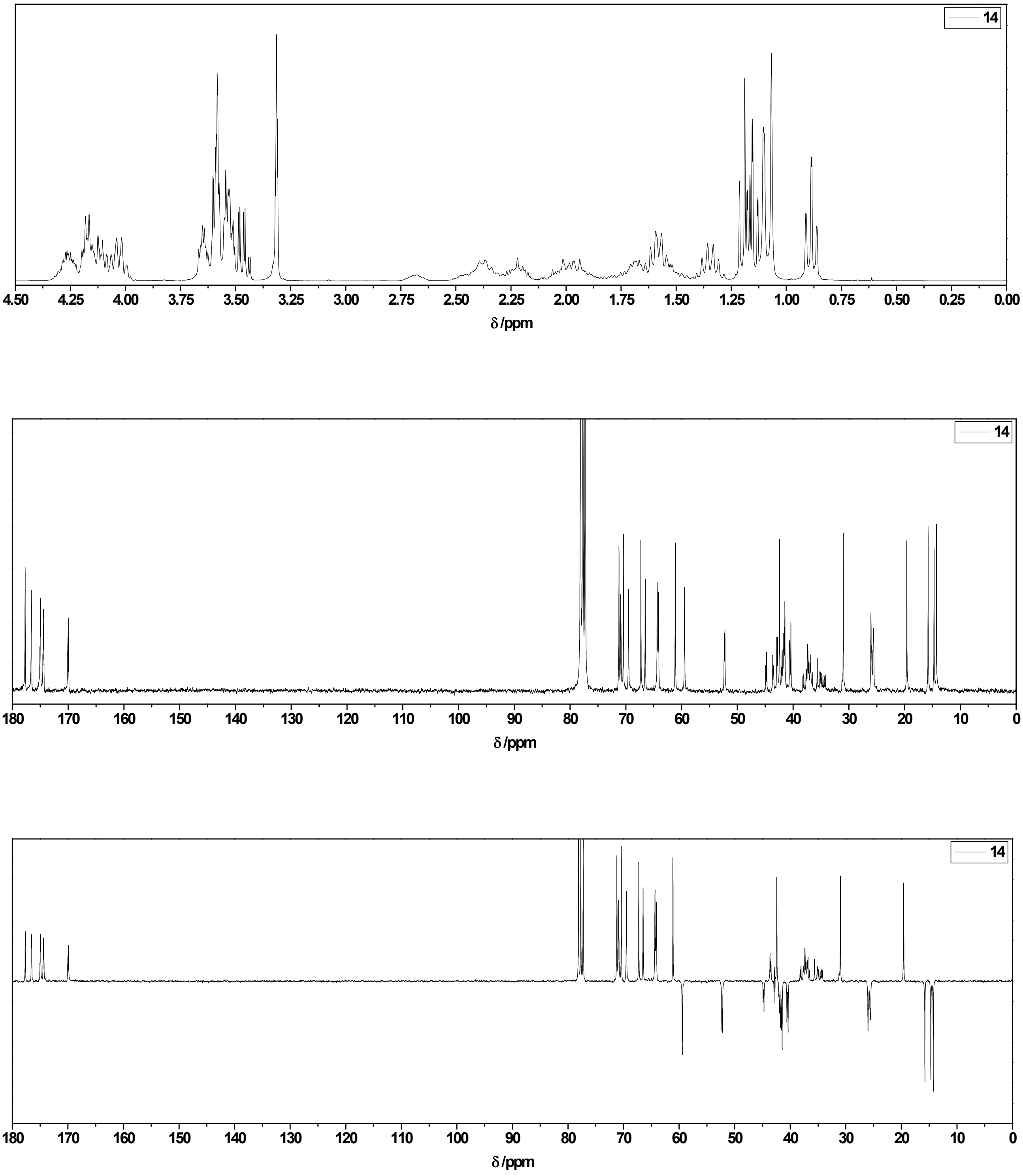

Figure S15. ^{1}H NMR, ^{13}C NMR and APT ^{13}C NMR spectrum of **I-MA-EGMEA-DEGEEA-BuA-Br 14**.

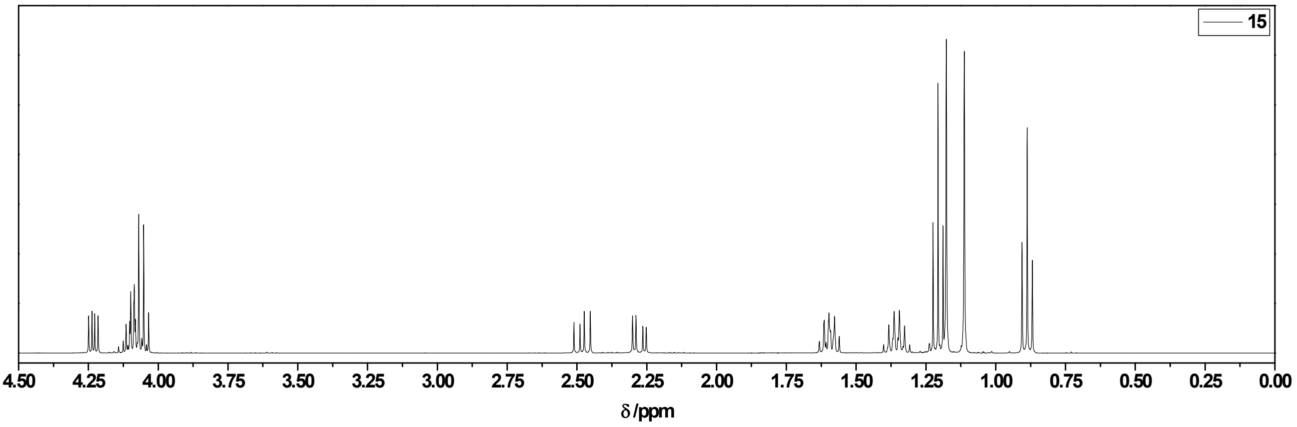

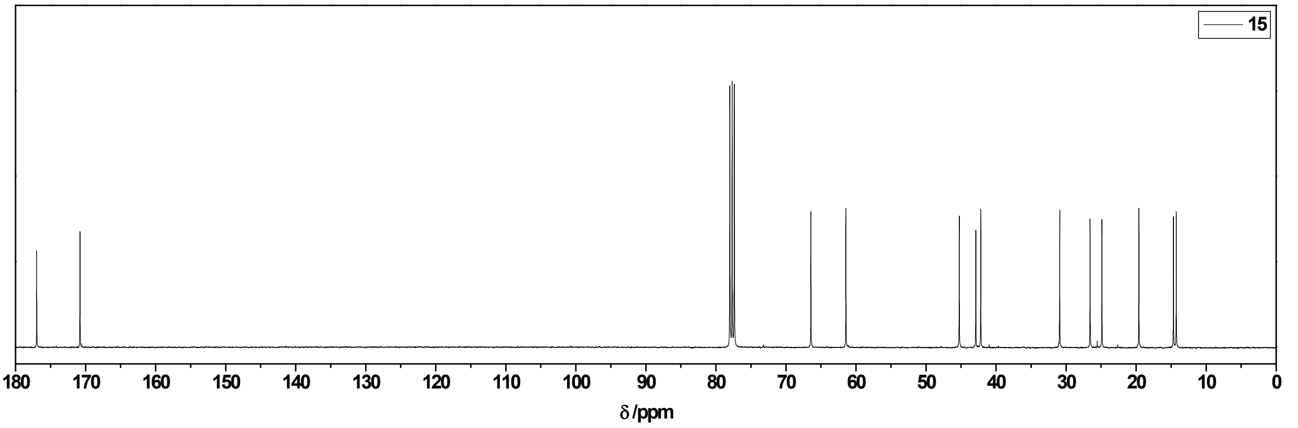

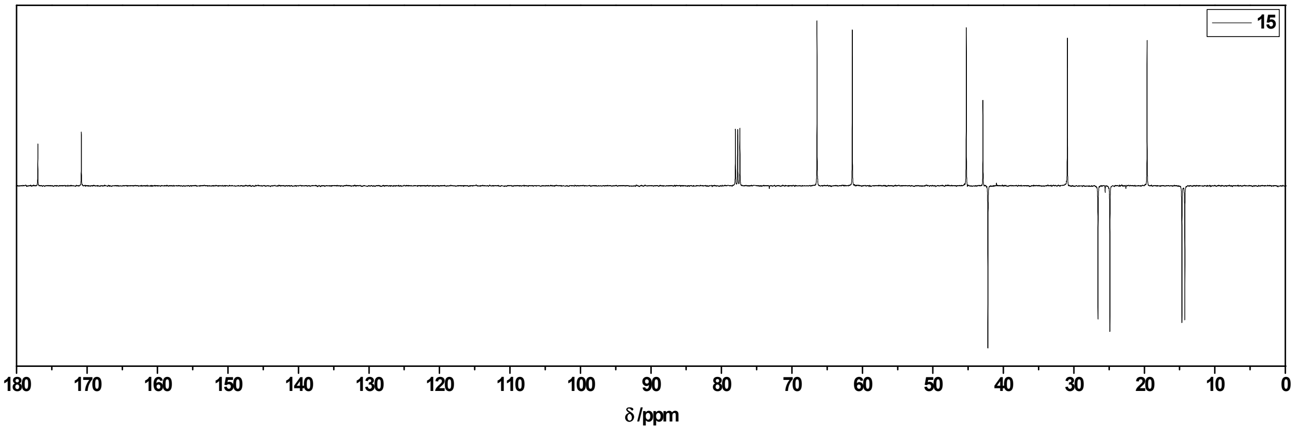

Figure S16. ^{1}H NMR, ^{13}C NMR and APT ^{13}C NMR spectrum of **I-BuA-Br 15**.

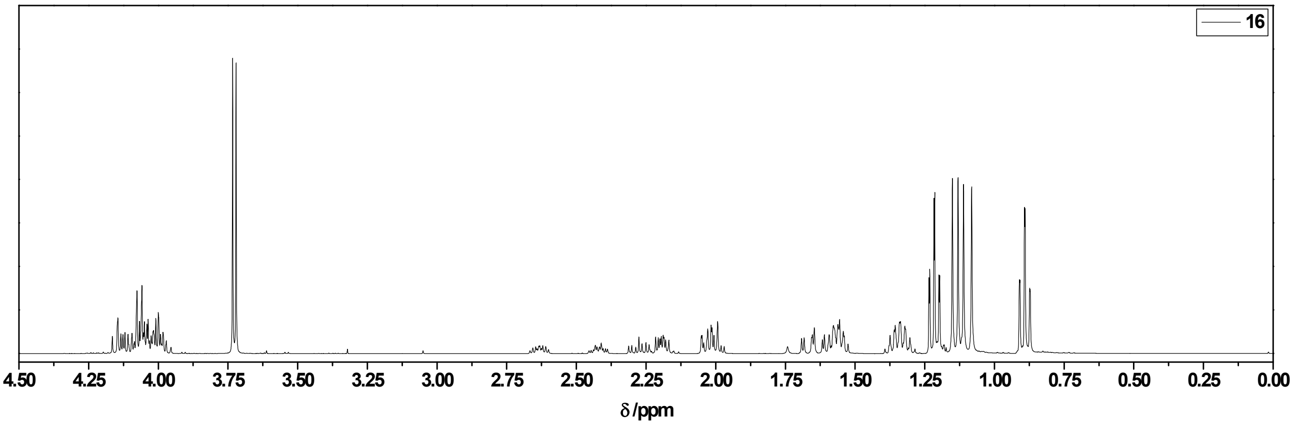

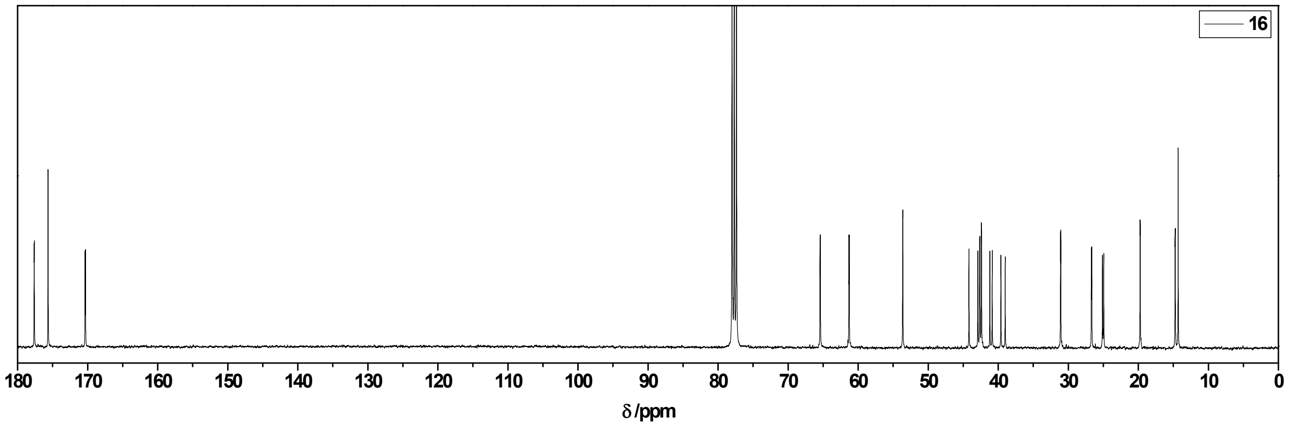

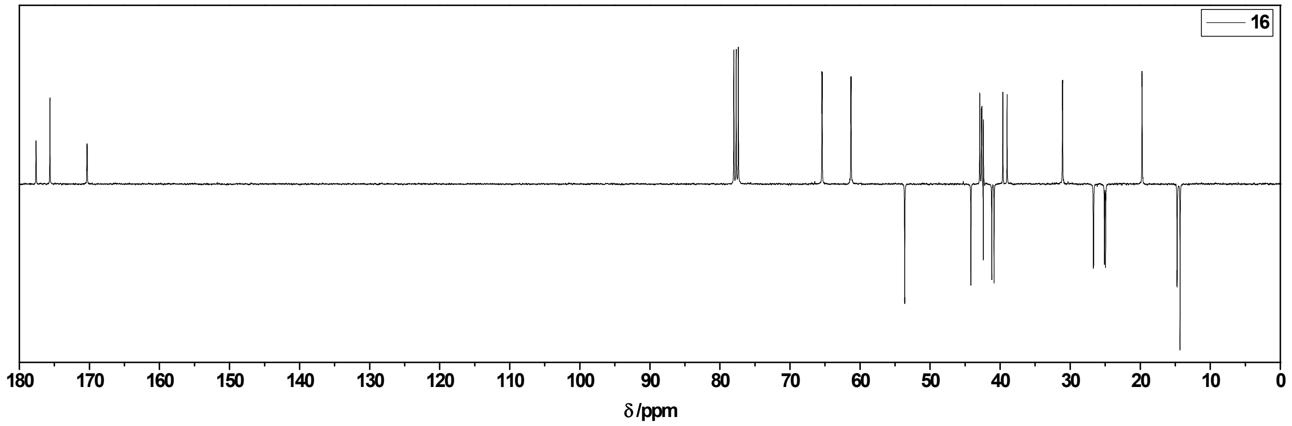

Figure S17. ^{1}H NMR, ^{13}C NMR and APT ^{13}C NMR spectrum of **I-BuA-MA-Br 16**.

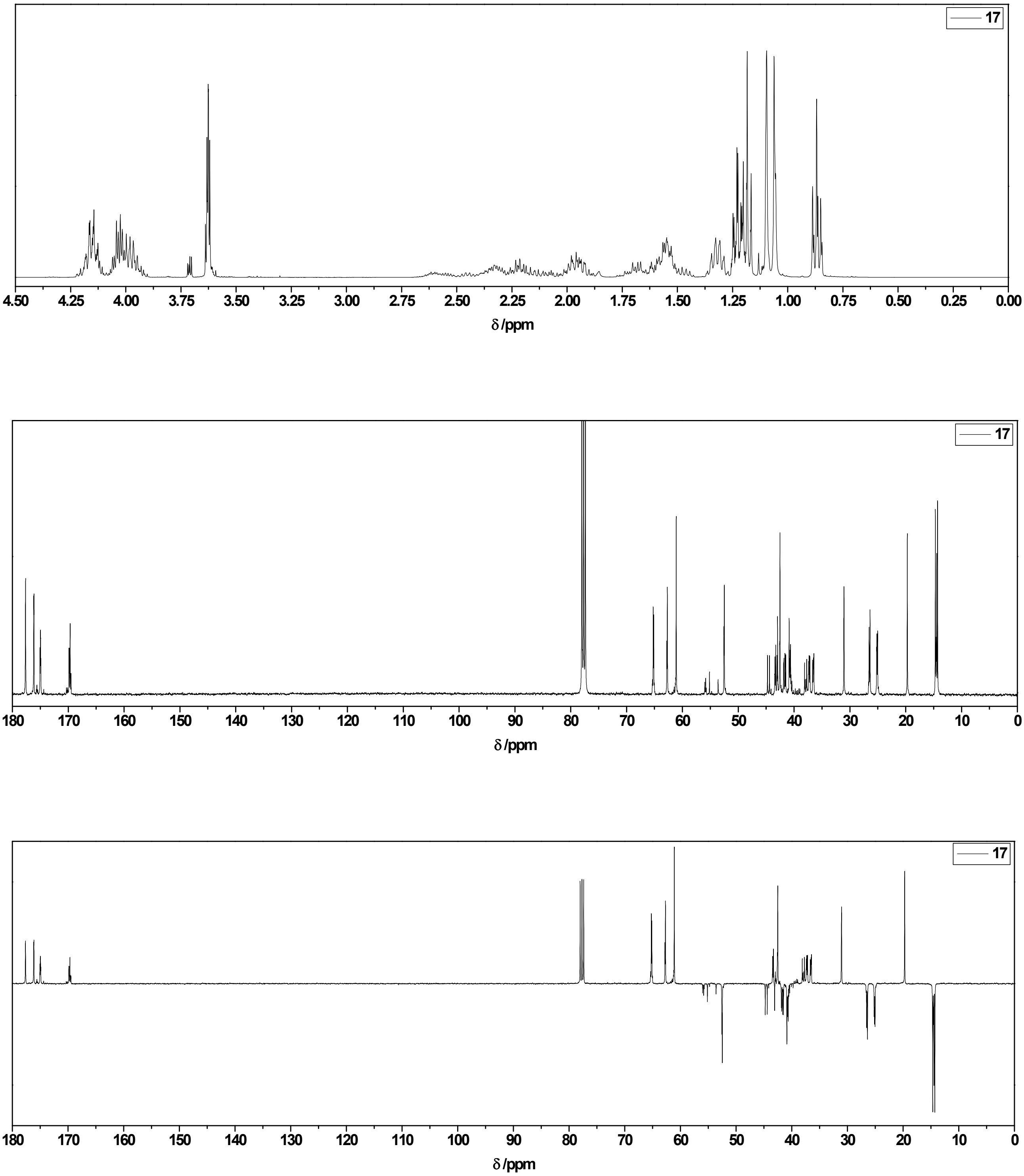

Figure S18. ^{1}H NMR, ^{13}C NMR and APT ^{13}C NMR spectrum of **I-BuA-MA-EA-Br 17**.

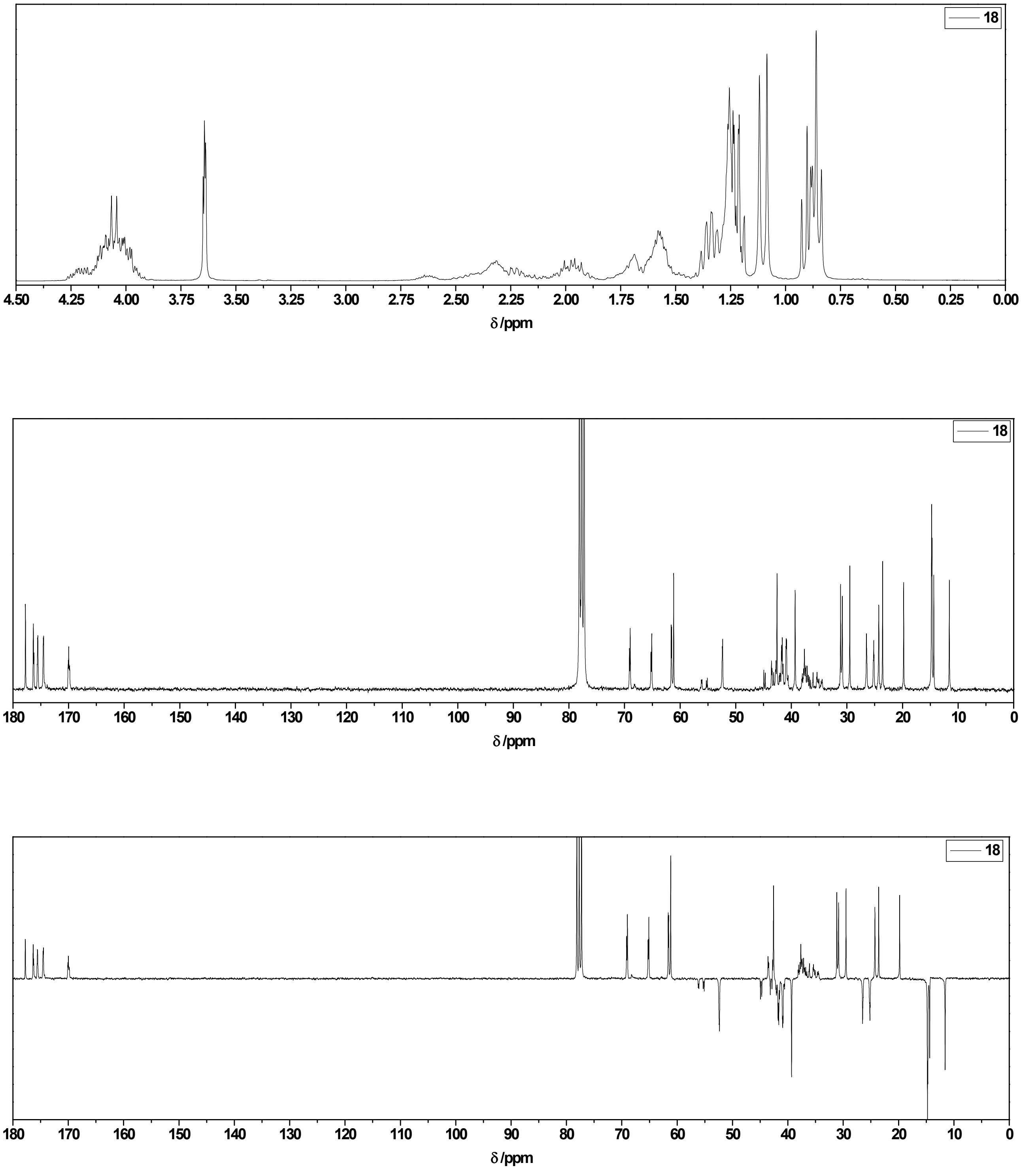

Figure S19. ^{1}H NMR, ^{13}C NMR and APT ^{13}C NMR spectrum of **I-BuA-MA-EA-EHA-Br 18**.

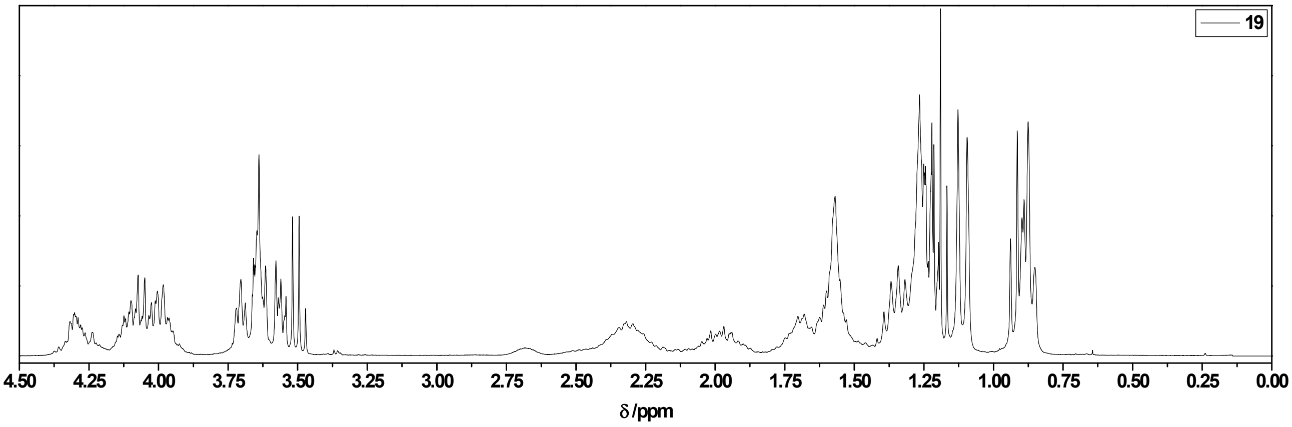

Figure S20. ^{1}H NMR spectrum of **I-BuA-MA-EA-EHA-DEGEEA-Br 19**.

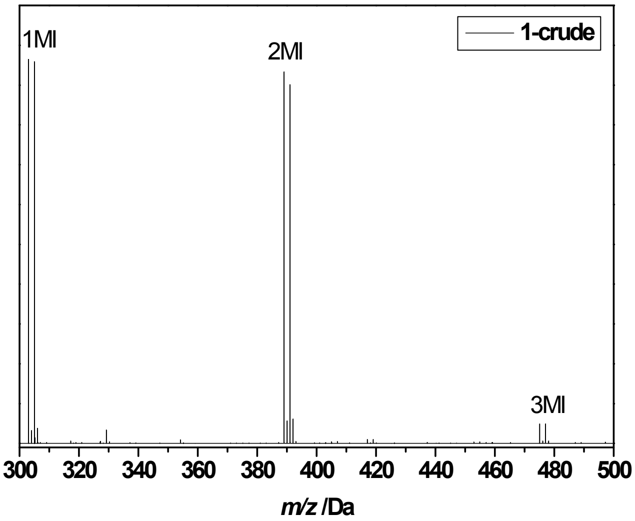

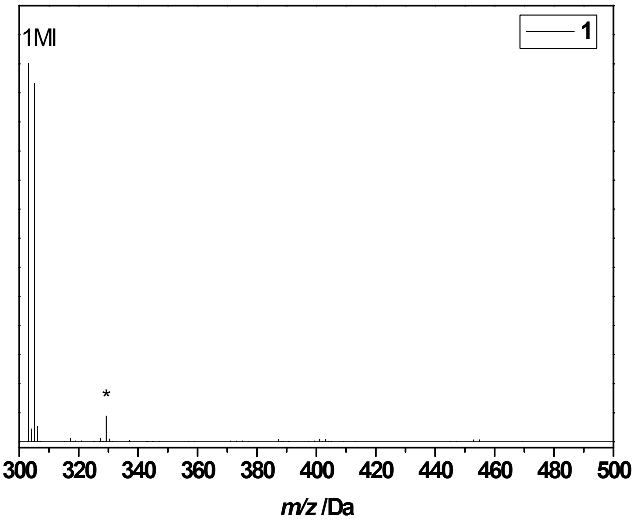

Figure S21. ESI-MS spectra of **I-MA-Br 1**, before and after purification. The peak marked with an asterisk results from a background signal.

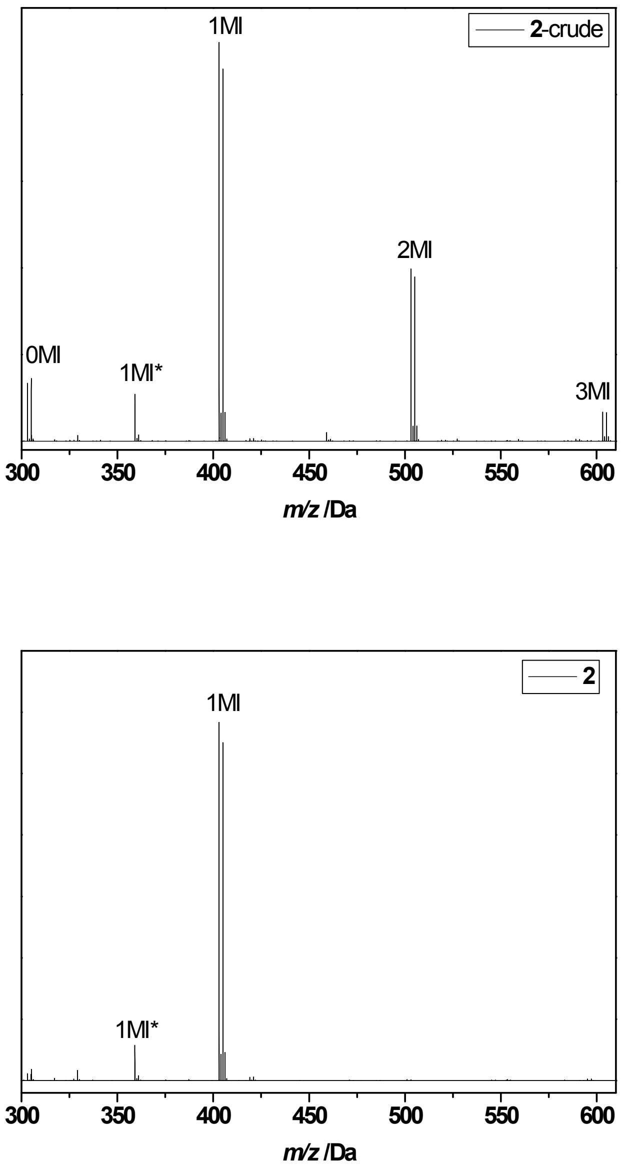

Figure S22. ESI-MS spectra of **I-MA-EA-Br 2**, before and after purification. The MI* peaks correspond to SUMI-Cl products resulting from halogen exchange.

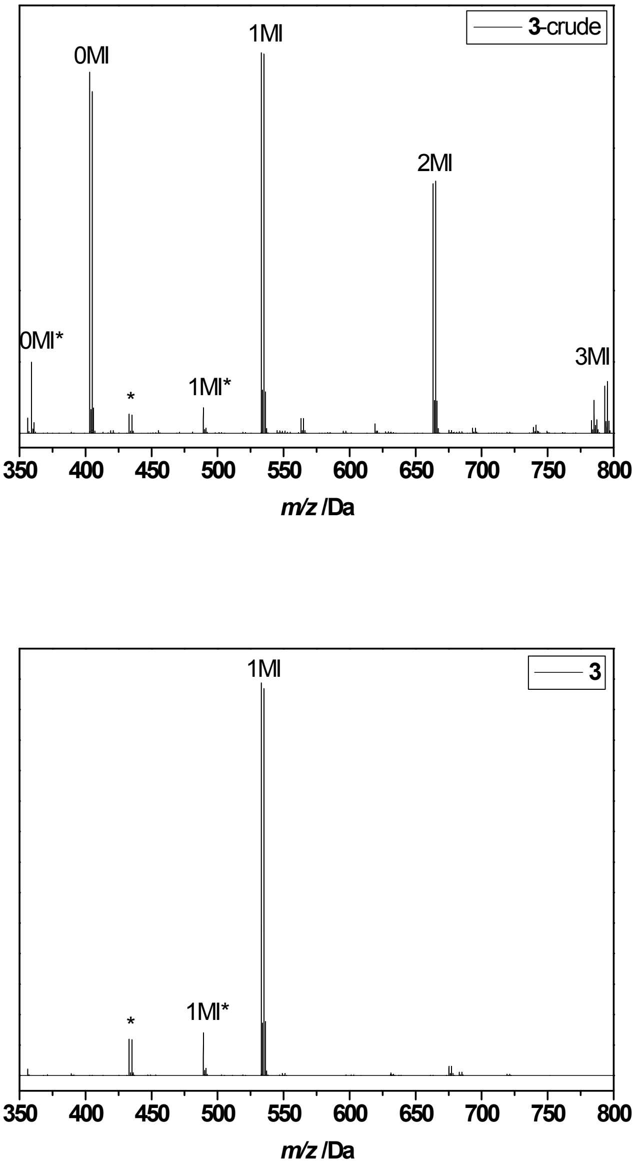

Figure S23. ESI-MS spectra of **I-MA-EA-EGMEA-Br 3**, before and after purification. The MI* peaks correspond to SUMI-Cl products resulting from halogen exchange, while the peak with an asterisk corresponds to MA-EGMEA-Br.

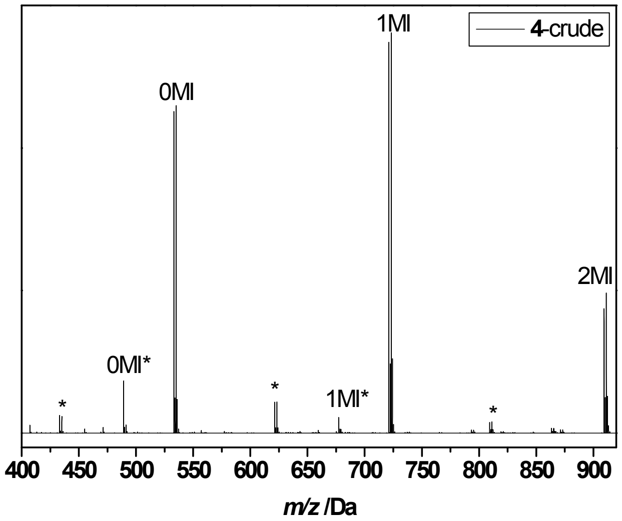

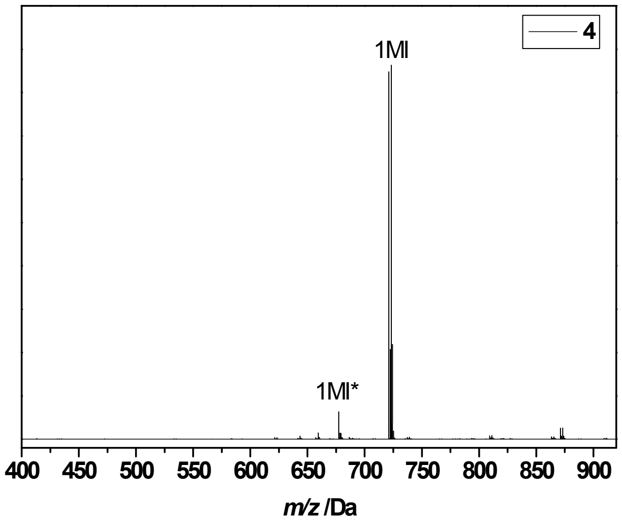

Figure S24. ESI-MS spectra of **I-MA-EA-EGMEA-DEGEEA-Br 4**, before and after purification. The MI* peaks correspond to SUMI-Cl products resulting from halogen exchange, while the peaks with an asterisk corresponds to MA-EGMEA-Br and MA-EGMEA-DEGEEA-Br oligomers.

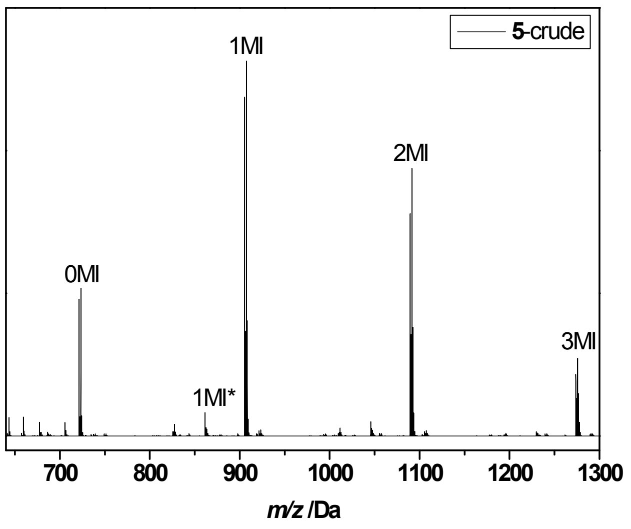

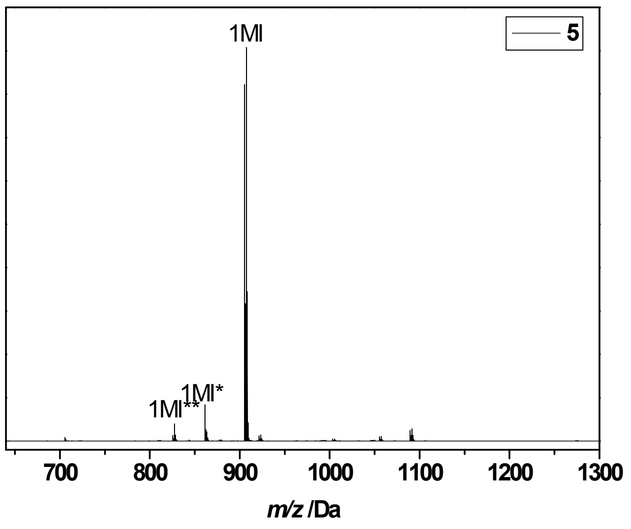

Figure S25. ESI-MS spectra of **I-MA-EA-EGMEA-DEGEEA-EHA-Br 5**, before and after purification. The MI* peak correspond to a SUMI-Cl product resulting from halogen exchange, and the MI** peak results from disproportionation (dead chain).

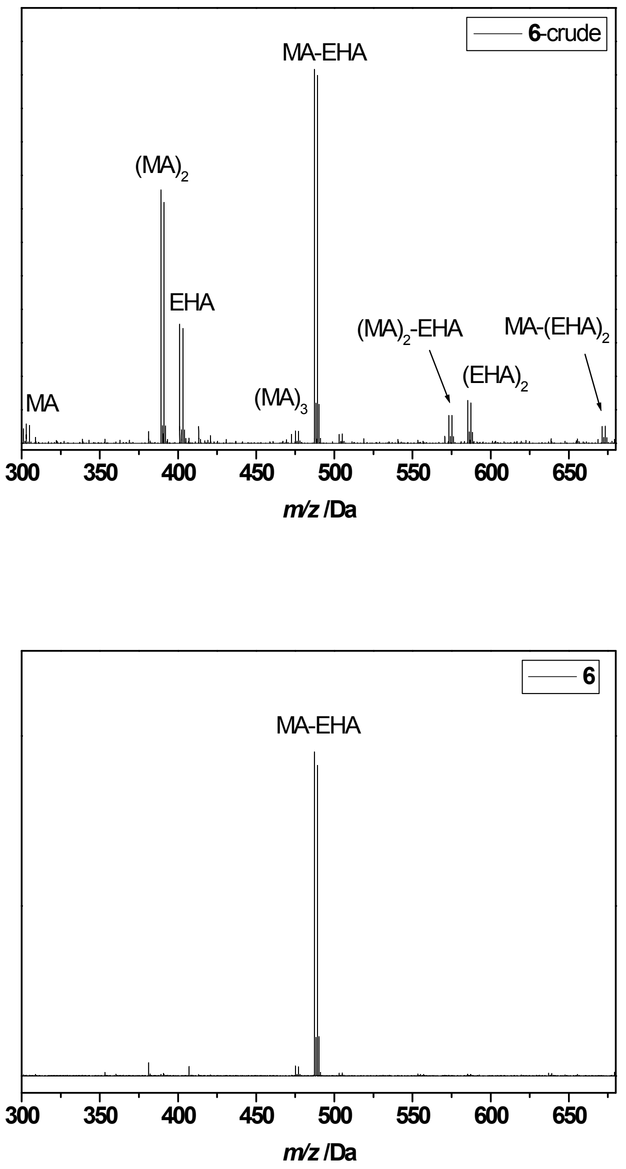

Figure S26. ESI-MS spectra of **I-MA-EHA-Br 6**, before and after purification. For synthesis of **6**, MA and EHA were inserted subsequently, without intermediate purification, so both homo-oligomers as well as MA-EHA block-co-oligomers are observed in the crude product.

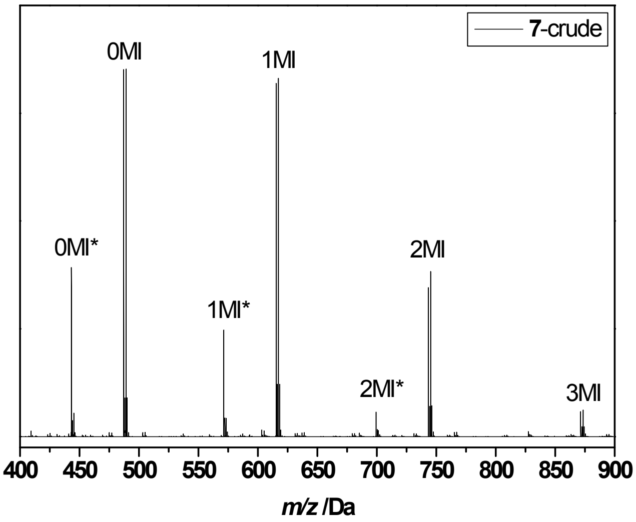

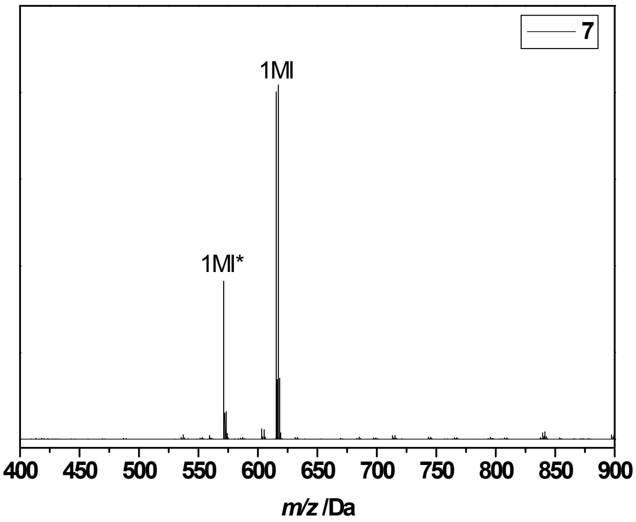

Figure S27. ESI-MS spectra of **I-MA-EHA-BuA-Br 7**, before and after purification. The MI* peaks correspond to SUMI-Cl products resulting from halogen exchange.

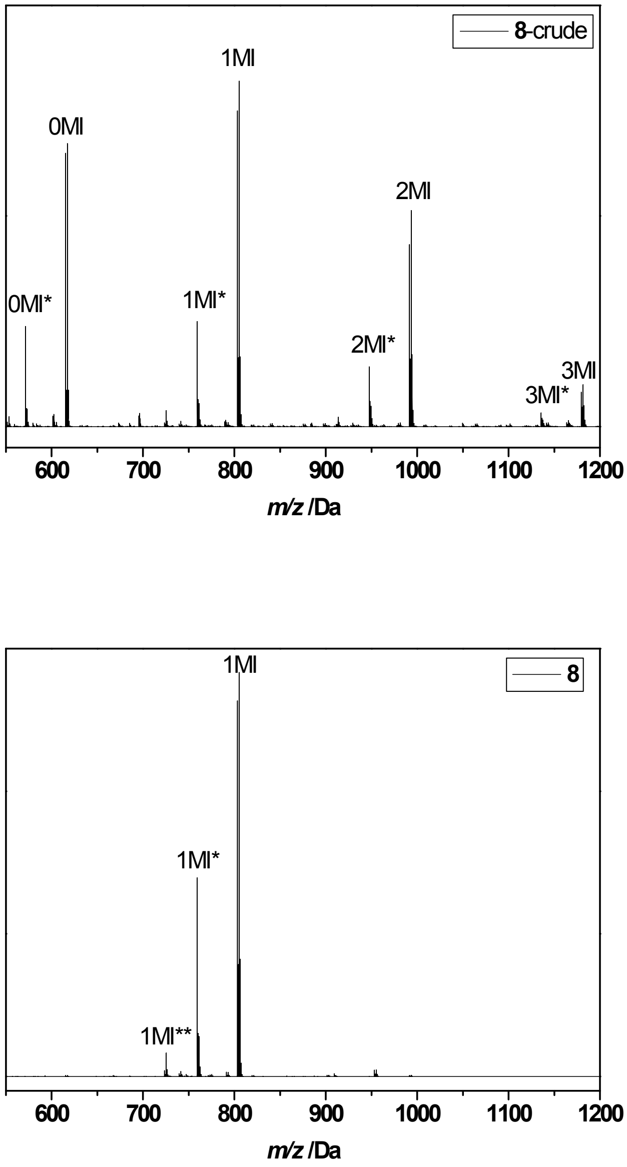

Figure S28. ESI-MS spectra of **I-MA-EHA-BuA-DEGEEA-Br 8**, before and after purification. The MI* peaks correspond to SUMI-Cl products resulting from halogen exchange, and the MI** peak results from disproportionation (dead chain).

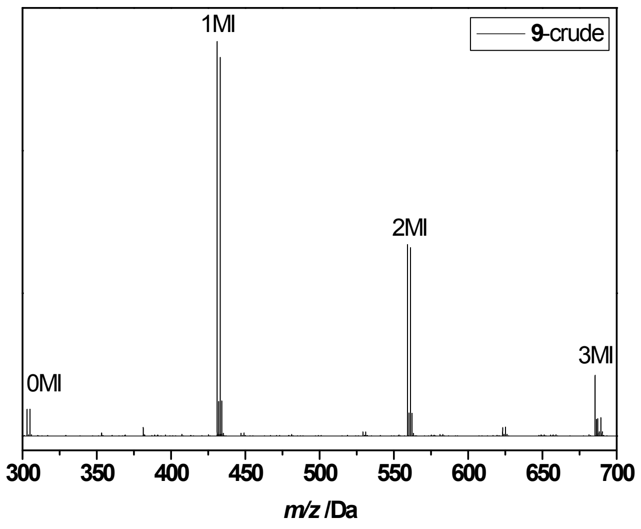

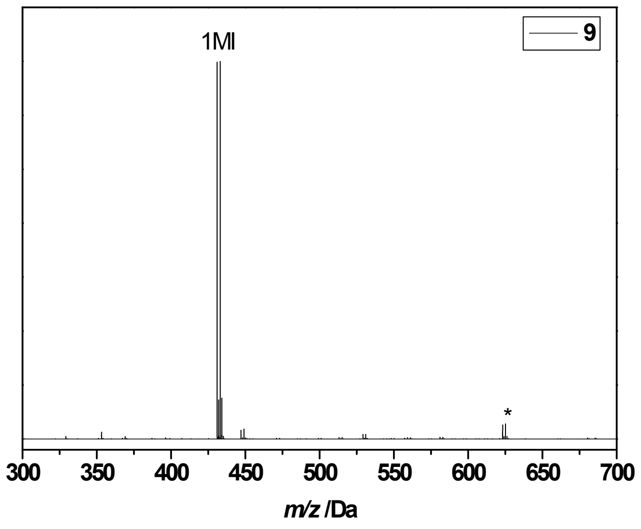

Figure S29. ESI-MS spectra of **I-MA-BuA-Br 9**, before and after purification. The peak marked with an asterisk results from a background signal.

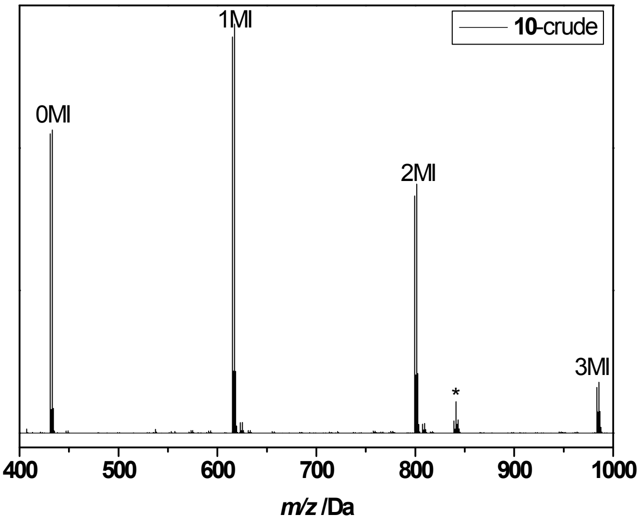

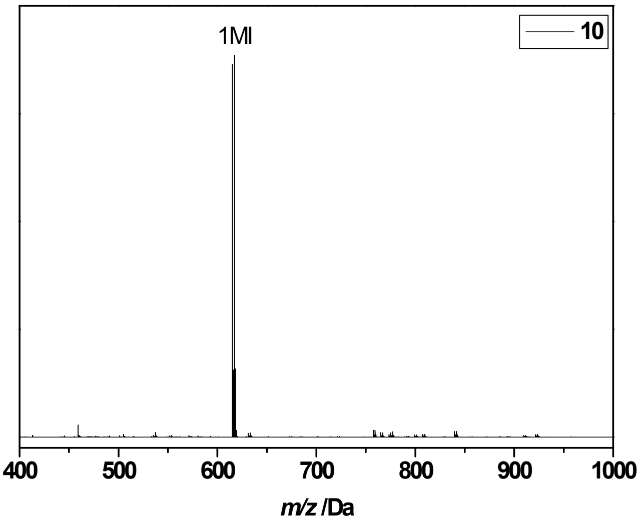

Figure S30. ESI-MS spectra of **I-MA-BuA-EHA-Br 10**, before and after purification. The peak marked with an asterisk corresponds to a complex of two 0MI molecules (charged with 1 Na^+), in-situ formed during the analysis.

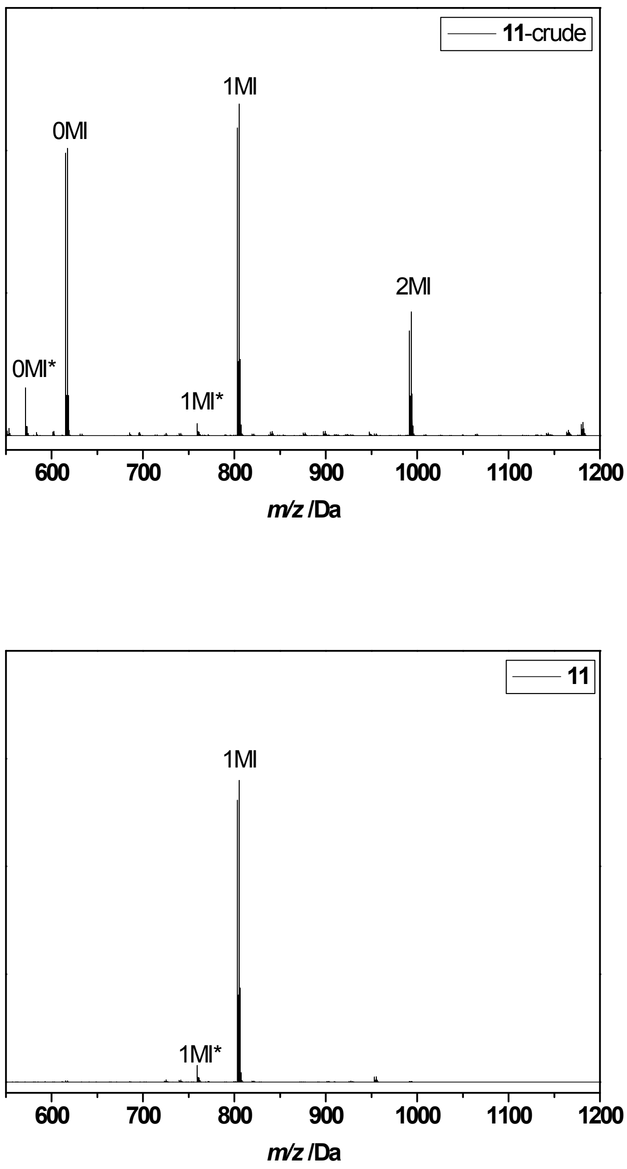

Figure S31. ESI-MS spectra of **I-MA-BuA-EHA-DEGEEA-Br 11**, before and after purification. The MI* peaks correspond to SUMI-Cl products resulting from halogen exchange.

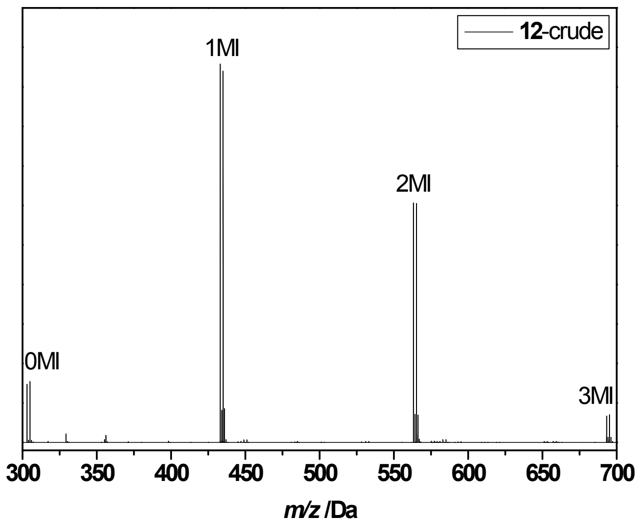

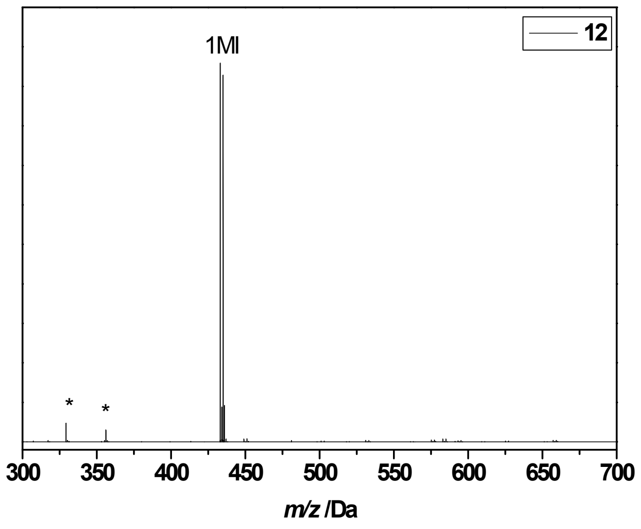

Figure S32. ESI-MS spectra of **I-MA-EGMEA-Br 12**, before and after purification. The peaks marked with an asterisk results from background signals.

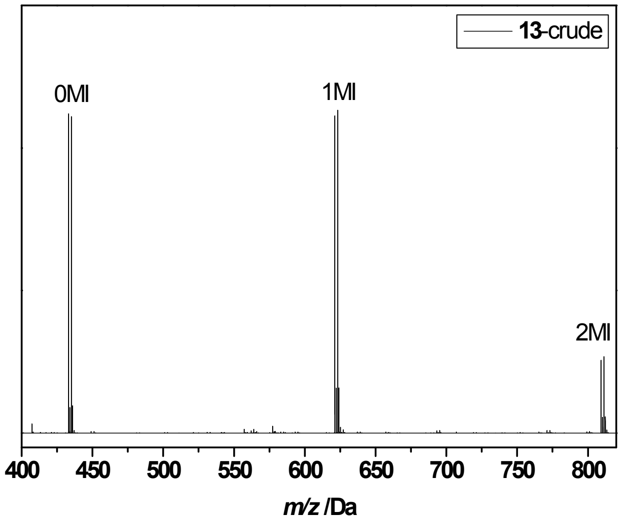

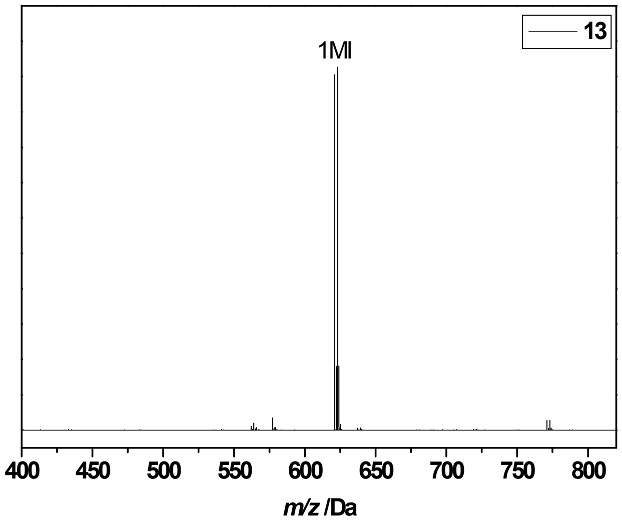

Figure S33. ESI-MS spectra of **I-MA-EGMEA-DEGEEA-Br 13**, before and after purification.

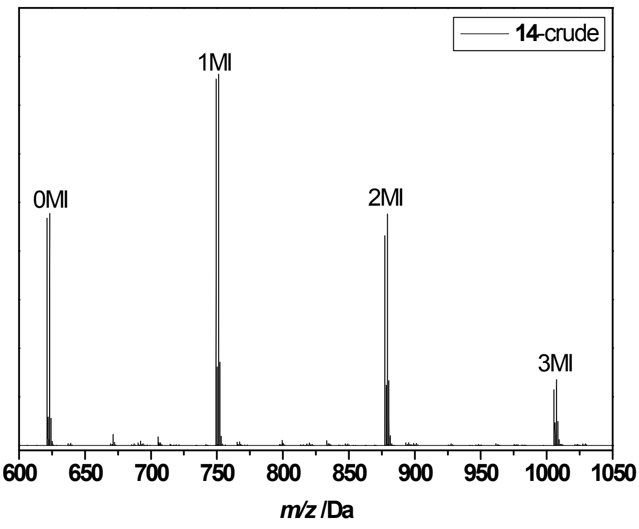

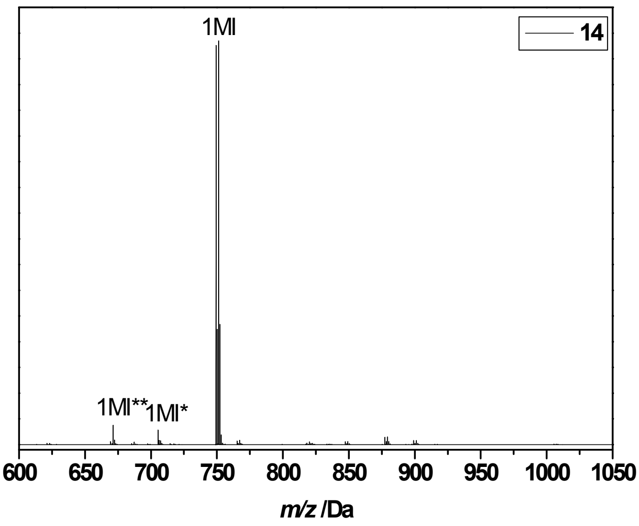

Figure S34. ESI-MS spectra of **I-MA-EGMEA-DEGEEA-BuA-Br 14**, before and after purification. The MI* peak corresponds to a SUMI-Cl product resulting from halogen exchange, and the MI** peak results from disproportionation (dead chain).

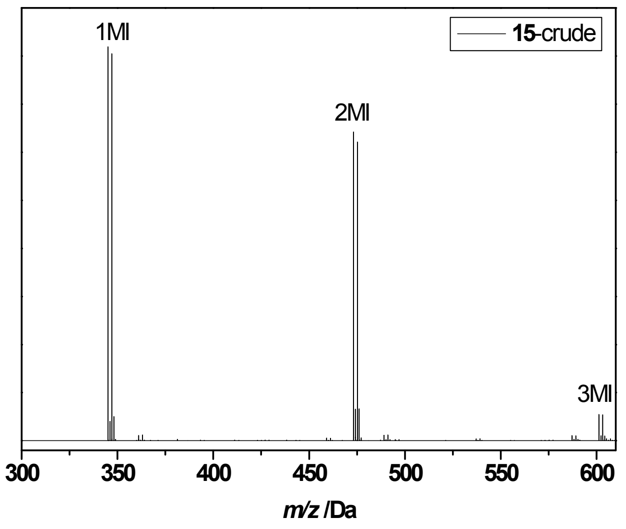

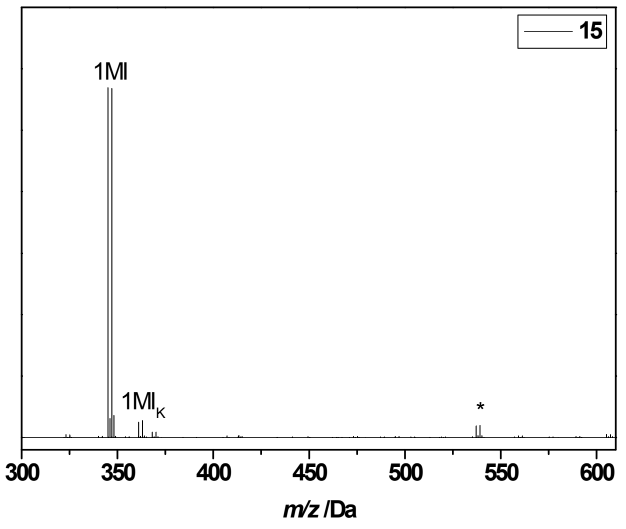

Figure S35. ESI-MS spectra of **I-BuA-Br 15**, before and after purification. The peak marked with $1MI_K$ corresponds to the 1MI product charged with K^+ and the peak marked with an asterisk corresponds to an in-situ formed complex of an 1MI and 0MI molecule, charged with Na^+.

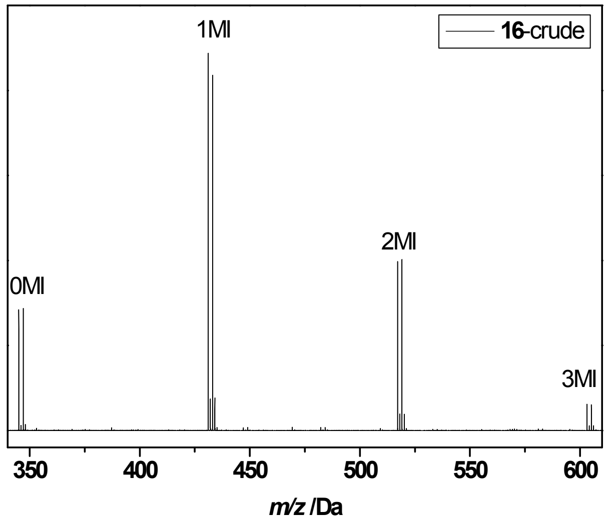

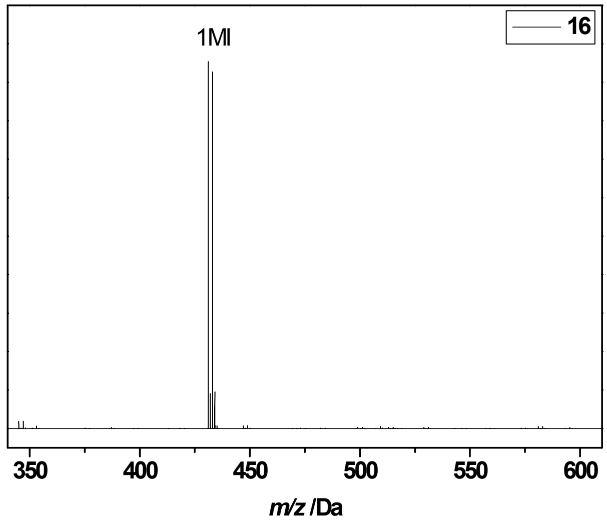

Figure S36. ESI-MS spectra of **I-BuA-MA-Br 16**, before and after purification.

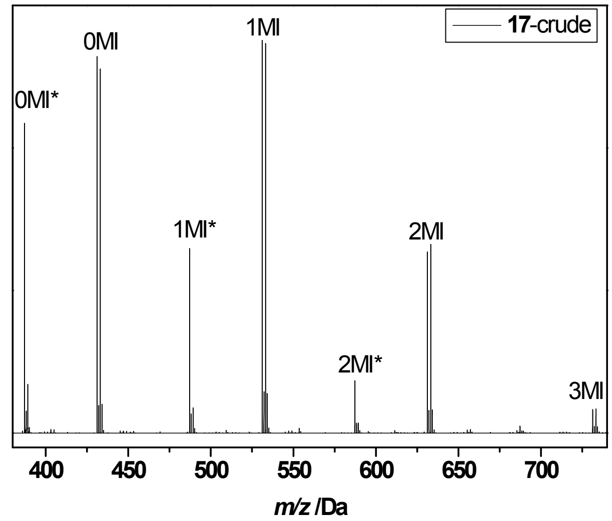

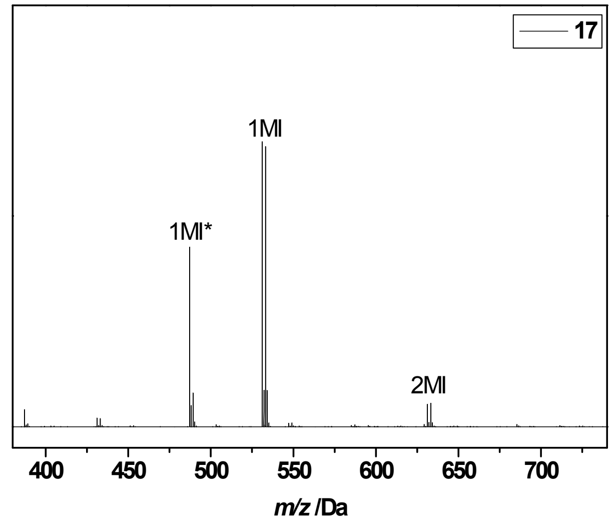

Figure S37. ESI-MS spectra of **I-BuA-MA-EA-Br 17**, before and after purification. The MI* peaks correspond to SUMI-Cl products resulting from halogen exchange.

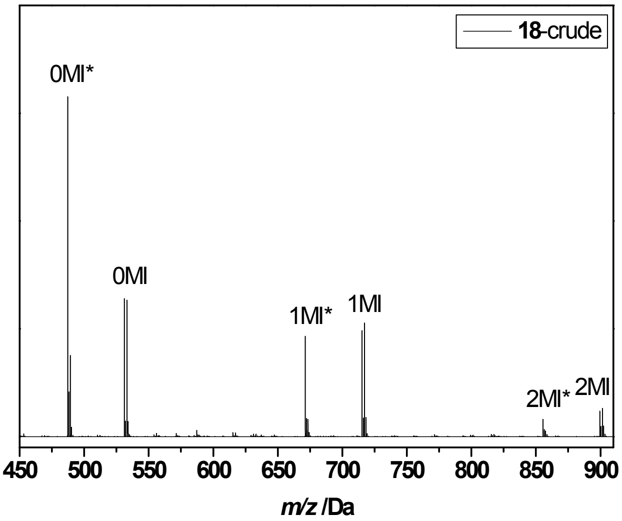

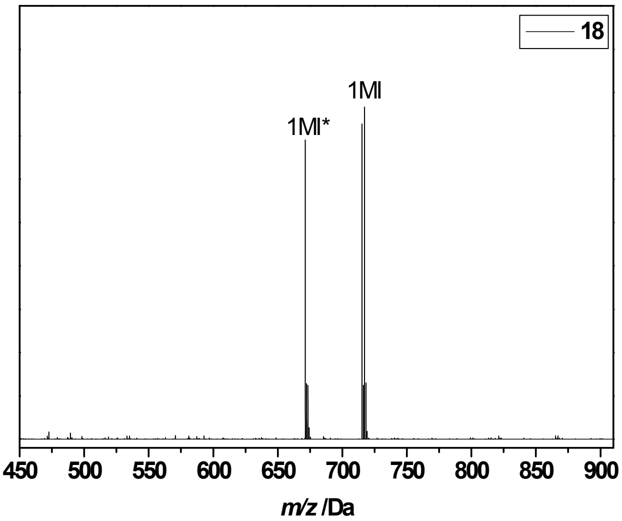

Figure S38. ESI-MS spectra of **I-BuA-MA-EA-EHA-Br 18**, before and after purification. The MI* peaks correspond to SUMI-Cl products resulting from halogen exchange.

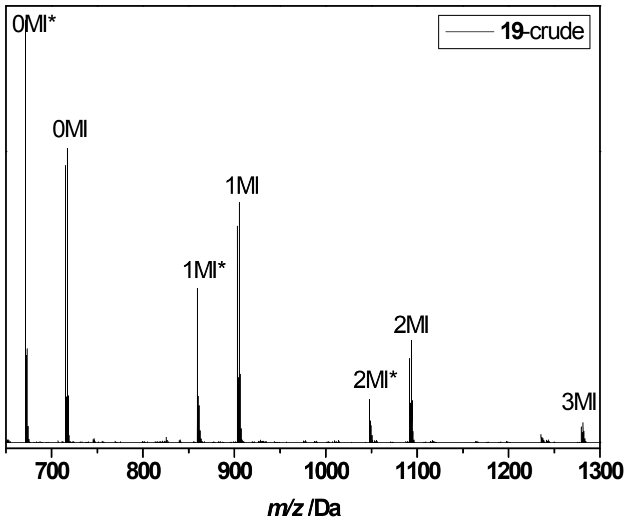

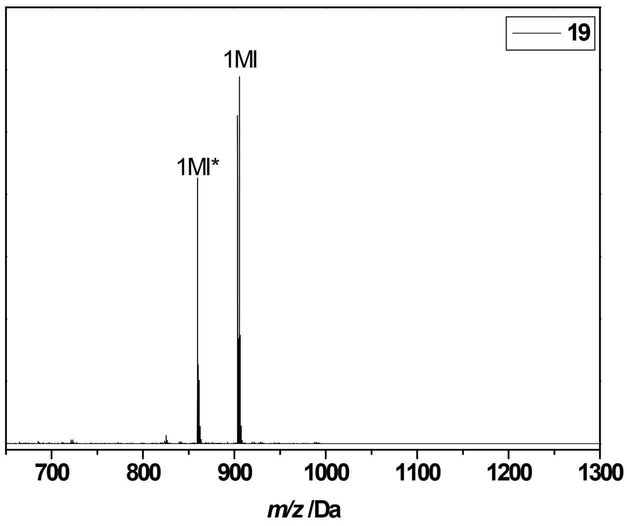

Figure S39. ESI-MS spectra of **I-BuA-MA-EA-EHA-DEGEEA-Br 19**, before and after purification. The MI* peaks correspond to SUMI-Cl products resulting from halogen exchange.

[1] Feng, L.; Hu, J.; Liu, Z.; Zhao, F.; Liu, G. *Polymer* **2007**, *48*, 3616-3623.
